# Effectiveness of iNTS vaccination in sub-Saharan Africa

**DOI:** 10.1038/s41598-025-87659-4

**Published:** 2025-01-30

**Authors:** Daniele Cassese, Nicola Dimitri, Gianluca Breghi, Tiziana Spadafina

**Affiliations:** 1 Department of Economics, University of Cambridge, Sidgwick Avenue, Cambridge, CB3 9DD Cambridgeshire UK; 2 Department of Economics, Universitá degli Studi di Siena, Piazza San Francesco, Siena, 53100 Italy; 3 Fondazione Achille Sclavo ONLUS, Via Fiorentina, Siena, 53100 Italy; 4 Sclavo Vaccines Association E.T.S., Via Fiorentina, Siena, 53100 Italy

**Keywords:** iNTS, Model, Vaccination, Effectiveness, Sub-Saharan Africa, Infectious diseases, Population dynamics

## Abstract

Invasive non-Typhoidal Salmonella (iNTS) is one of the leading causes of blood stream infections in Sub-Saharan Africa, especially among children. iNTS can be difficult to diagnose, particularly in areas where malaria is endemic, and difficult to treat, partly because of the emergence of antibiotic resistance. We developed a mathematical model to evaluate the impact of a vaccine for iNTS in 49 countries of sub-Saharan Africa. Without vaccination we estimate 9.2 million new iNTS cases among children below 5 years old in these 49 countries from 2022 to 2038, 6.2 million of which between 2028 and 2038. The introduction of a $85\%$ ($95\%$) efficacy vaccine in 2028 would prevent 2.6 (2.9) million of these new infections. We provide the country-specific impact of a iNTS vaccine considering the different age structures and vaccine coverage levels.

## Introduction

Non-typhoidal *Salmonella* invasive (iNTS) disease is an emerging neglected infectious disease that causes a serious global burden of morbidity and mortality. The first global estimates of iNTS disease, produced as part of the Global Burden of Disease (GBD) 2017, showed that iNTS disease affects more than half a million people [535,000 (409,000–705,000) cases] with an average Case Fatality Rates (CFR) of about $15\%$^1,2^ and 4.26 million (2.38–7.38) DALYs. Most recently, iNTS disease has been globally associated to 87,100 [53,800–131,000] deaths due to bloodstream infection, with an age mortality rate of 1.2 years^3^. iNTS is among the 10 top pathogens in the estimation of the years of life lost (YLL) burden^3^. Data on iNTS incidence and the prevalence of complications and case-fatality ratio (CFR) of iNTS^2^ has been systematically reviewed on behalf of the Vacc-iNTS Consortium^4^. They have calculated a global incidence ($95\%$ CI) of 44.8 (31.5–60.5) per 100,000 persons per year, with Africa significantly higher [51.0 (36.3–68.0)] and estimated that approximately $15\%$ of patients with iNTS disease die, similarly to^1^. Approximately $70\%$ of currently reported iNTS cases are observed in sub-Saharan Africa (sSA)^1,4^, where it is among the leading cause of community-acquired bloodstream infections and is associated with increasing antibiotic resistance^5–9^. In sSA, iNTS disease spreads mostly among children under 5 years of age, with comordibities as malaria, anaemia, malnutrition, and HIV infection being prominent risk factors^1,4^ together with young age. In adults, HIV infection is by far the most important risk factor. These infections usually present as a febrile illness, frequently without gastrointestinal symptoms in both young adults and children, leading to severe, extra-intestinal, invasive bacteremia. iNTS disease has been reported across Africa, demonstrating it is a widespread threat throughout the continent^3,4,10,11^ with indication that the disease is endemic in much of the Region.

Over the past years, it has emerged that the serovars *Enteritidis* and *Typhimurium* are the ones most commonly associated with iNTS in Africa, causing more than $90\%$ of cases^4,10,12^ characterized by genome degradation and appear to be adapting to an invasive lifestyle. Recent estimates have been showing the increasing emergence of multidrug-resistant (MDR) *S. Typhimurium* and *S. Enteritidis* strains especially in sSA^5,13–15^, which compromises the clinical treatment of iNTS disease in settings where diagnosis, surveillance programmes and affordable medicines are often scarce^16^. *Salmonella* spp. have been included in the World Health Organization (WHO) antibiotic-resistant high priority pathogens list, and show a concerning increase in MDR^13,17^. iNTS causes major disease and socioeconomic burden in resource-poor communities, particularly in children, elderly people, and people with HIV infection in sSA, and no vaccine is currently available^3,18–20^. Medical need, difficult diagnosis and increasing AMR make the investigation of the sources and transmission pathways of iNTS disease a crucial point to implement effective preventive and control measures and definitely strongly advocate for rapid development of an effective vaccine.

Several iNTS vaccines are currently under development, some of which are bivalent and targeting the two serovars leading causes of iNTS, and include live attenuated, protein-polysaccharide vaccines, multiple antigen presenting system complexes and Outer Membrane Vesicles-based vaccines. The ideal iNTS vaccine should be poor reactogenic, cross-protective against multiple serovars and at affordable production and delivery costs. Among them the generalised modules for membrane antigens (GMMA) of *S.* enterica serovars *Enteritidis* and *Typhimurium* expressed on outer membrane vesicles. The development of this candidate has been carried out also in the context of the S-AFRIVAC project (supported by the Tuscany Region) and is advancing under the H2020 Vacc-iNTS and EDCTP2 PEDVAC- iNTS projects.

To evaluate the impact of the introduction of a vaccine against iNTS on the African burden we developed a mathematical model for the transmission of iNTS disease. We simulate the transmission dynamic for each country separately, using country-specific population pyramids and comorbidity data, as well as vaccine coverage rates. The effect of the introduction of the iNTS vaccine has been evaluated comparing two different scenarios: the status quo, without any intervention, and a vaccination scenario where we consider a catch-up campaign followed by a routine campaign. The catch-up campaign starts in 2028 and lasts for one year, during which children between 9 months and 5 years of age are vaccinated, while the routine vaccination campaign lasts 9 years, during which children are vaccinated upon reaching 9 months of age. The epidemiological trajectories are projected up to 2038, and we consider two levels of vaccine efficacy, $85\%$ and $95\%$.

## Results

**Fig. 1:**
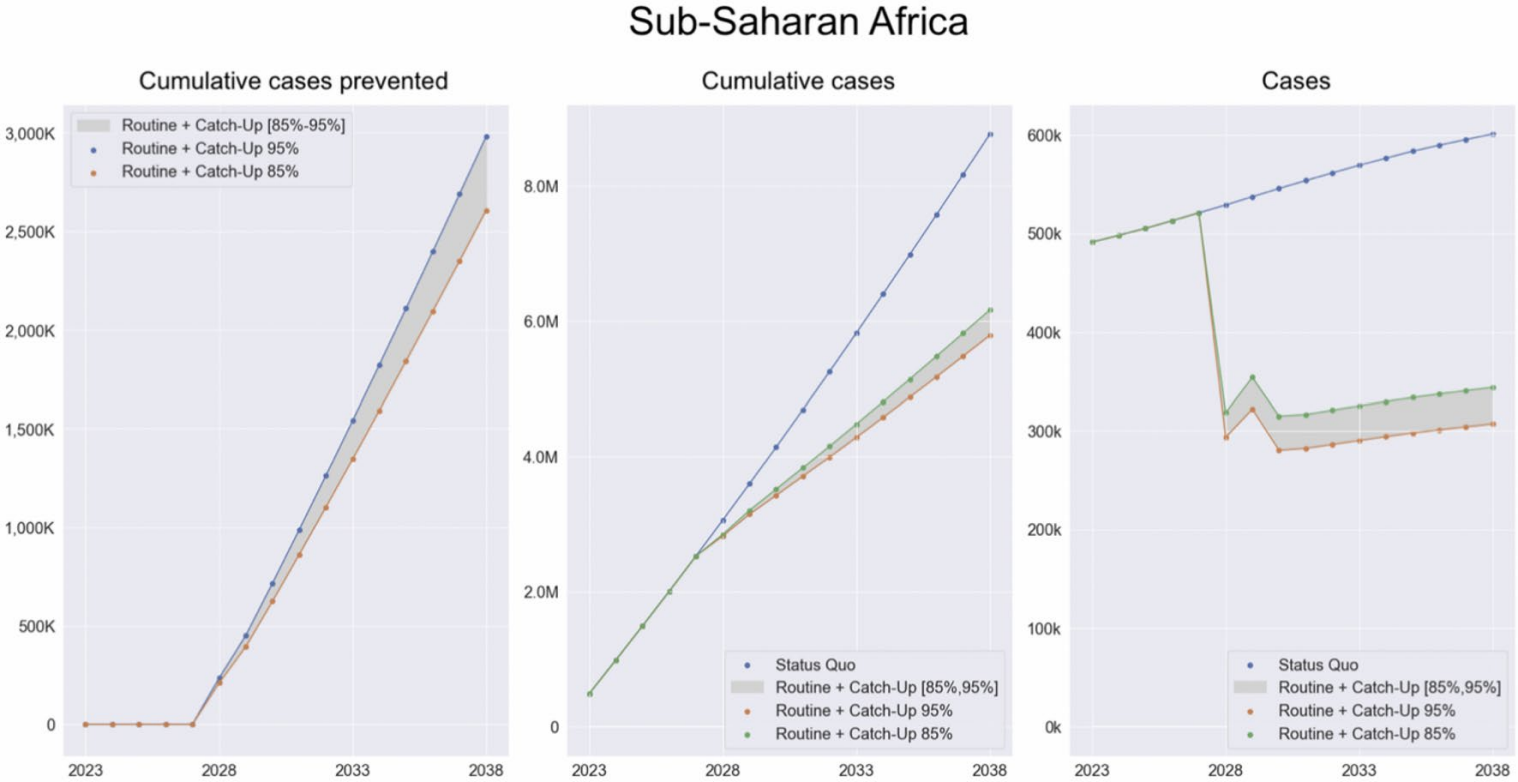
Cumulative cases prevented, cumulative cases and yearly cases for sSA. Cumulative number of cases prevented with a vaccination campaign over 10 years, starting with a one year catch-up followed by a 9-years routine vaccination over the period 2028–2038 (left). Cumulative number of cases in the status quo scenario, without vaccination and in the vaccination scenario (centre). Yearly number of cases for the status quo and vaccination scenario (right). Vaccine efficacy is between $85\%$ and $95\%$. Cases aggregated across countries in sSA.

**Fig. 2:**
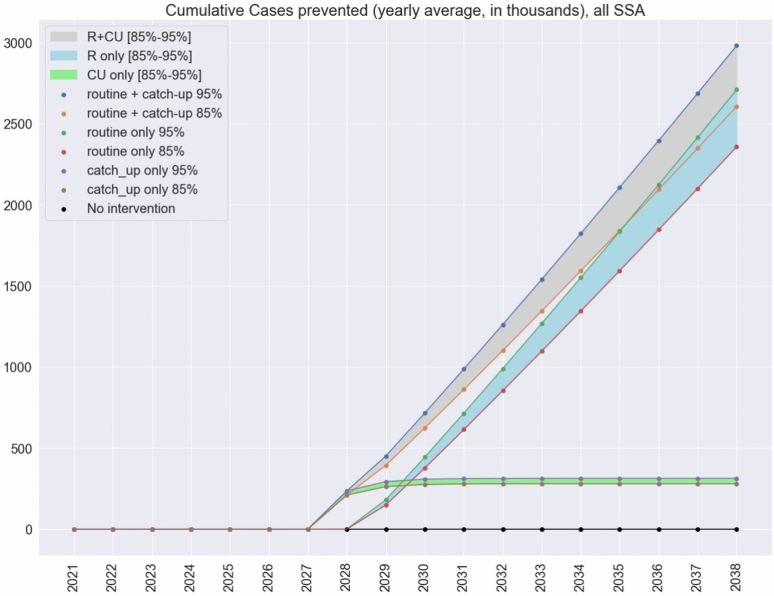
Cumulative cases prevented in the three vaccination scenarios. The three vaccination scenarios are Routine only, Routine + Catch-Up and Catch-Up only. Vaccine efficacy between $85\%$ and $95\%$. Values in thousands.

Our model estimates 478,000 iNTS cases for the year 2021 in all sSA, and under the status quo scenario the cumulated number of cases estimated from 2021 to 2038 is 9,733,000 (6,242,000 of which in the period 2028-2038). The 10 years Routine + Catch-Up (RCU) campaign between 2028 and 2038 could prevent between 2,605,000 and 2,981,000 cases when the vaccine efficacy is between 85$\%$ and 95$\%$ (Fig. 1 Cumulative cases prevented, cumulative cases and yearly cases for sSA. and Fig. 2 Cumulative cases prevented in the three vaccination scenarios). This means that the model estimates a reduction of iNTS cases between 41.7$\%$ and 47.8$\%$ among children below 5 years old in all sSA. If we assume a CFR of $15\%$ the vaccine would avert between 391,000 and 447,000 deaths over the 10 years considered. Based on the model projections, the 10-year vaccination campaign would reduce the burden of the disease by 34,187,000–39,117,000 DALYs for all sSA (Table 1 Per-country cumulative cases prevented, deaths and DALYs averted (RCU)).

**Fig. 3:**
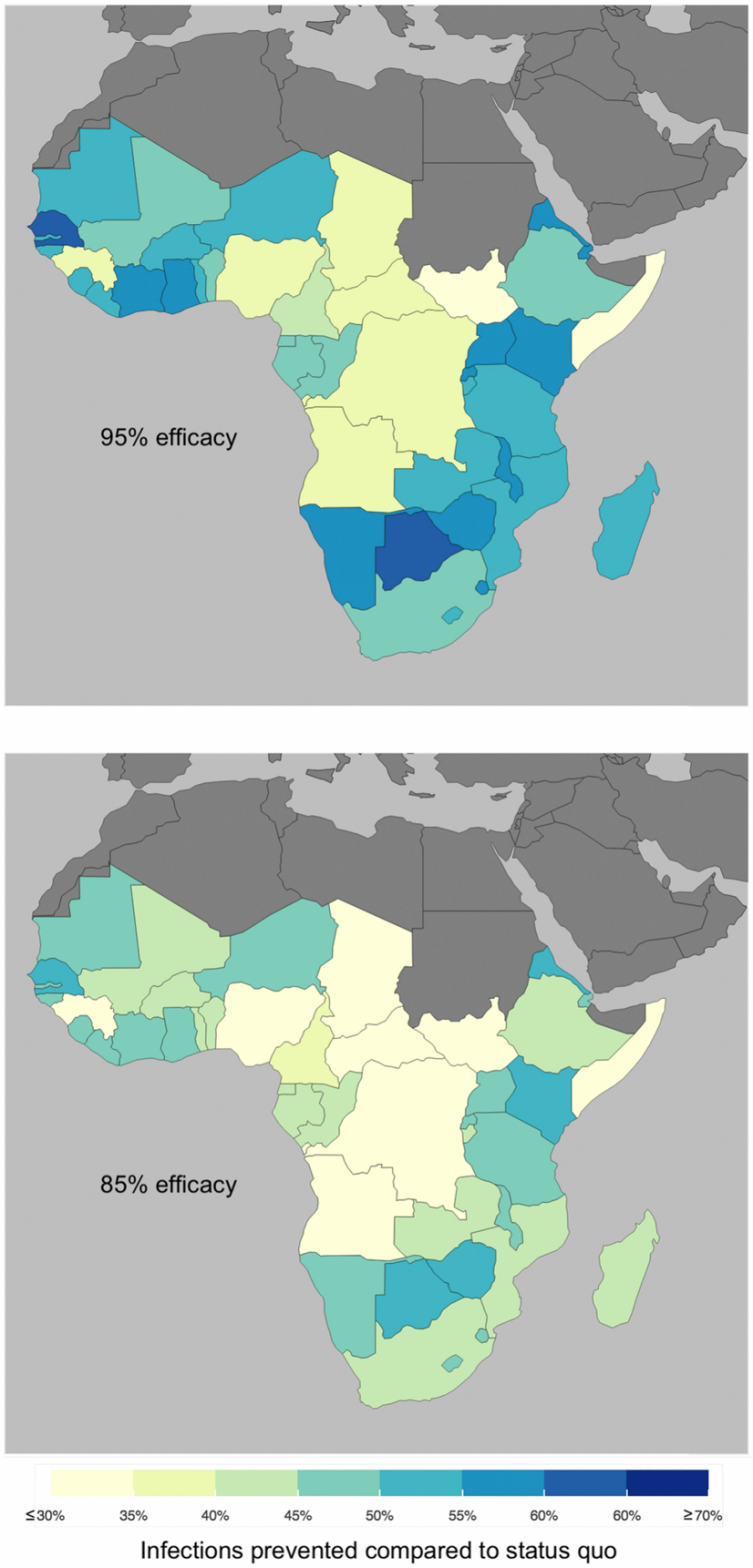
Vaccine efficacy as percentage of prevented infections per country. Infections prevented between 2028 and 2038, one year Catch-Up followed by 9 years Routine. Vaccine assumed with 95$\%$ efficacy (*Top*) and 85$\%$ efficacy (*Bottom*). Differences across countries mostly due to routine vaccination coverage and prevalence of comorbidities.

**Fig. 4:**
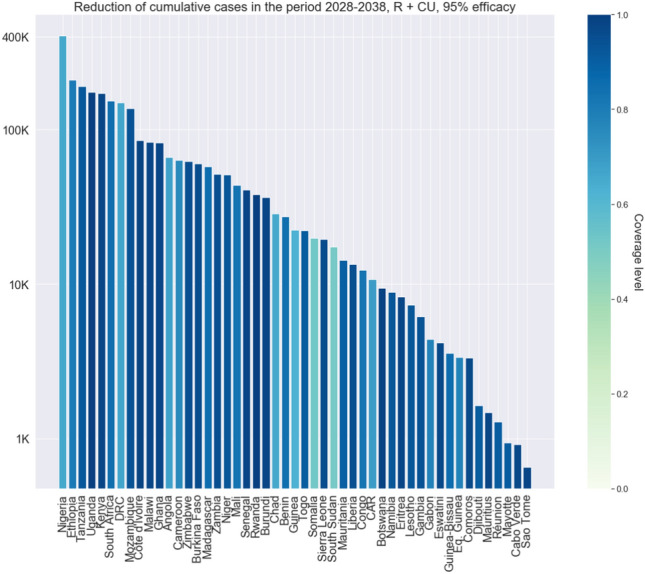
Cumulative number of cases prevented per country. Bars show the number of cases prevented considering a one year catch-up followed by a routine vaccination over the period 2028–2038. Vaccine efficacy is $95\%$. The *y*-axis is in logarithmic scale. The colour of each bar corresponds to the routine coverage level in the country, as captured by the colormap on the righthand side of the graph.

There are considerable differences in cases prevented across countries, due to their differences in the prevalence of comorbidities among children below 5 years old and EPI vaccination coverage levels (Fig. 3 Vaccine efficacy as percentage of prevented infections per country and Supplementary Figs. 4–8). For countries where EPI coverage levels are below $70\%$, the reduction in iNTS cases reaches at most $34$–$38\%$ ($85\%$ and $95\%$ efficacy respectively). For countries that have a coverage at least of $95\%$, the model predicts that the reduction in cases can go above $50$–$60\%$ ($85\%$ and $95\%$ efficacy respectively) (Fig. 4 Cumulative number of cases prevented per country). Countries where the prevalence of comorbidities is higher (especially Malaria among children) present smaller reduction in cases given their coverage levels (Supplementary Fig. 8).

Nigeria and Ethiopia account for the highest number of cases prevented among children below 5 years old over the considered period ([362,000–404,000] (35–38$\%$) and [186,000–210,000] (42–48$\%$) respectively), together with the Democratic Republic of the Congo (DRC) which accounts for [133,000–150,000] (32–36%) cases prevented. Both Nigeria and DRC present low levels of coverage (65$\%$ and 66$\%$ respectively, against 80$\%$ for Ethiopia) and this explains the relatively lower reduction in cases following vaccination. Assuming a coverage of 90$\%$ for the routine vaccination the number of prevented cases would substantially increase: our model predicts [487,000–556,000] (47–53$\%$) cases prevented for Nigeria, [179,000–206,000] (44–50$\%$) for DRC and [209,000-238,000] (47–54$\%$) for Ethiopia. Tanzania and Uganda are less populous than the three countries mentioned, but thanks to higher coverage levels (91$\%$ and 99$\%$ respectively) they count for a large share of infections prevented, at [167,000–191,000] (47–54$\%$) and [147,000–174,000] (49–58$\%$). Our results show that increasing EPI vaccination coverages across countries would induce a considerable reduction in the burden of iNTS: by assuming an EPI coverage of at least 90$\%$ in each country the aggregate number of cases prevented over the period goes up to 2,996,000–3,373,000, (47–54$\%$).

We focused on a Routine + Catch-Up campaign as the number of cases prevented is the highest under this scenario, but we also analysed other two scenarios, Routine (R) only and Catch-Up only. As can be seen in Fig. 2 Cumulative cases prevented in the three vaccination scenarios and Supplementary Table 4, the difference between R and RCU in terms of cases prevented is in the order of [248,000–272,000] cases over 10 years under the two efficacy scenarios. Catch-up campaigns are costly and we are simulating a best-case scenario in terms of catch-up coverage, as discussed in “Data and calibration” section, so cost-benefit analysis should be conducted to evaluate which scenario is better. For completeness we include the estimation of DALYs averted with Routine only in Supplementary Table 5.

**Table 1:** Per-country cumulative cases prevented, deaths and DALYs averted (RCU). All number are in thousands. The lower bound of cases prevented, deaths averted and DALYs averted correspond to 85$\%$ vaccine efficacy, the upper bound to 95$\%$ vaccine efficacy.

Country	Status quo			Vaccination - catch-up + routine		
	Cases	Deaths	DALYs	Cases prevented	Deaths averted	DALYs averted
Angola	193.409	43.847	2538	(65.795, 73.659)	(9.869, 11.049)	(863, 967)
Benin	64.274	14.879	844	(26.886, 30.499)	(4.033, 4.575)	(353, 401)
Botswana	17.168	4.194	225	(8.924, 10.42)	(1.339, 1.563)	(117, 137)
Burkina Faso	133.042	30.748	1746	(56.911, 67.085)	(8.537, 10.063)	(747, 880)
Burundi	78.375	18.243	1028	(34.077, 40.63)	(5.112, 6.094)	(447, 533)
Cabo Verde	1.671	0.426	22	(0.872, 1.005)	(0.131, 0.151)	(11, 13)
Cameroon	161.300	37.731	2117	(62.883, 70.574)	(9.432, 10.586)	(825, 926)
CAR	30.435	7.133	399	(10.566, 11.844)	(1.585, 1.777)	(139, 155)
Chad	83.618	19.419	1097	(28.265, 31.601)	(4.24, 4.74)	(371, 415)
Comoros	6.872	1.634	90	(3.158, 3.706)	(0.474, 0.556)	(41, 49)
Congo	29.459	6.806	386	(12.098, 13.68)	(1.815, 2.052)	(158, 179)
Côte d’Ivoire	164.364	38.204	2157	(80.289, 94.726)	(12.043, 14.209)	(1053, 1243)
DRC	453.291	104.500	5948	(147.597, 165.409)	(22.14, 24.811)	(1937, 2170)
Djibouti	3.215	0.808	42	(1.585, 1.797)	(0.238, 0.27)	(21, 24)
Equatorial Guinea	8.241	1.912	108	(3.306, 3.715)	(0.496, 0.557)	(43, 48)
Eritrea	15.418	3.636	202	(7.884, 9.183)	(1.183, 1.377)	(103, 120)
Eswatini	8.058	1.960	105	(3.979, 4.628)	(0.597, 0.694)	(52, 60)
Ethiopia	486.369	116.275	6383	(206.646, 232.389)	(30.997, 34.858)	(2712, 3050)
Gabon	10.591	2.570	139	(4.327, 4.857)	(0.649, 0.729)	(57, 64)
Gambia	12.756	2.985	167	(5.919, 6.834)	(0.888, 1.025)	(78, 90)
Ghana	158.806	37.742	2084	(77.991, 91.232)	(11.699, 13.685)	(1024, 1197)
Guinea	68.041	15.883	893	(22.118, 24.703)	(3.318, 3.705)	(291, 325)
Guinea-Bissau	7.643	1.811	100	(3.508, 3.958)	(0.526, 0.594)	(46, 52)
Kenya	318.218	75.191	4176	(164.178, 190.72)	(24.627, 28.608)	(2154, 2503)
Lesotho	15.160	3.753	199	(7.024, 8.083)	(1.054, 1.212)	(92, 106)
Liberia	28.154	6.582	370	(12.829, 14.912)	(1.924, 2.237)	(169, 196)
Madagascar	127.544	29.717	1674	(56.444, 63.903)	(8.467, 9.585)	(741, 839)
Malawi	166.239	38.545	2181	(78.392, 92.302)	(11.759, 13.845)	(1029, 1211)
Mali	106.669	24.532	1400	(42.962, 48.675)	(6.444, 7.301)	(564, 639)
Mauritania	29.569	6.893	388	(13.923, 15.83)	(2.088, 2.375)	(183, 208)
Mauritius	2.706	0.676	35	(1.395, 1.62)	(0.209, 0.243)	(18, 21)
Mayotte	2.152	0.506	28	(0.908, 1.046)	(0.136, 0.157)	(12, 13)
Mozambique	300.967	69.591	3949	(130.585, 151.98)	(19.588, 22.797)	(1713, 1994)
Namibia	17.691	4.287	232	(8.581, 9.816)	(1.287, 1.472)	(113, 129)
Niger	109.446	24.464	1436	(49.729, 57.335)	(7.459, 8.6)	(652, 752)
Nigeria	1,159.397	269.397	15,215	(400.67, 447.48)	(60.101, 67.122)	(5258, 5873)
Rwanda	74.075	17.711	972	(35.425, 42.297)	(5.314, 6.345)	(464, 555)
Réunion	2.570	0.633	33	(1.25, 1.419)	(0.188, 0.213)	(16, 18)
Sao Tome	1.206	0.281	16	(0.626, 0.727)	(0.094, 0.109)	(9, 10)
Senegal	74.910	17.475	983	(39.103, 45.341)	(5.865, 6.801)	(513, 595)
Seychelles	0.272	0.069	4	(0.146, 0.171)	(0.022, 0.026)	(2, 2)
Sierra Leone	40.570	9.763	532	(18.513, 21.626)	(2.777, 3.244)	(243, 284)
Somalia	68.344	15.595	897	(19.689, 21.934)	(2.953, 3.29)	(258, 287)
South Africa	338.967	83.555	4448	(149.607, 168.932)	(22.441, 25.34)	(1963, 2217)
South Sudan	63.586	14.992	835	(17.184, 19.16)	(2.578, 2.874)	(226, 252)
Tanzania	390.524	89.390	5124	(186.192, 212.806)	(27.929, 31.921)	(2443, 2792)
Togo	48.580	11.313	638	(21.538, 24.703)	(3.231, 3.705)	(283, 324)
Uganda	330.125	78.227	4332	(162.81, 193.292)	(24.421, 28.994)	(2137, 2537)
Zambia	110.407	25.320	1449	(49.302, 57.45)	(7.395, 8.618)	(647, 754)
Zimbabwe	117.331	28.117	1540	(60.613, 69.218)	(9.092, 10.383)	(796, 908)

## Methods

### Mathematical model

**Fig. 5:**
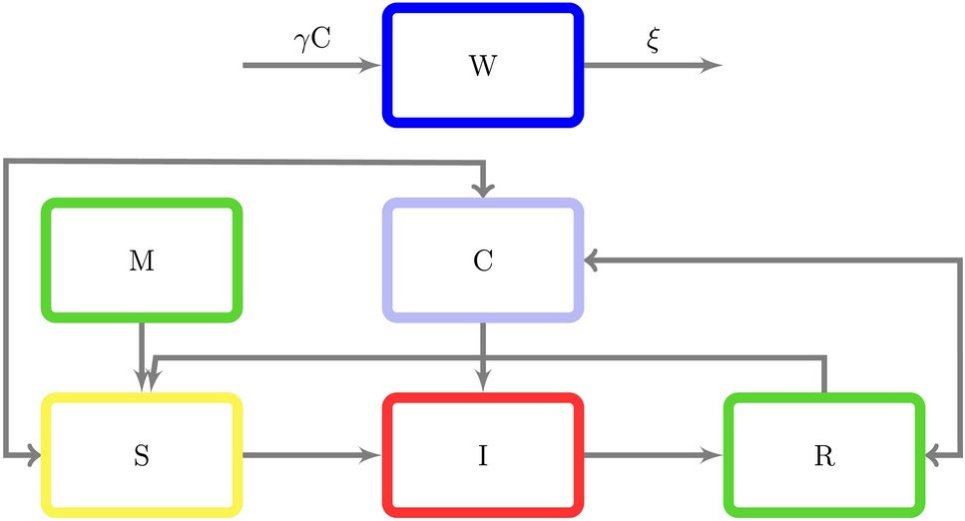
Diagram of the compartmental model. Age and comorbidity groups not shown. Individuals are born into the maternal immunity compartment *M*, then transition to the susceptible compartment *S* where they can become infected *I* if they are below 5 y or above 5 and with HIV. After the infection they will transition to the recovered compartment *R*, where they are immune, and upon waning of immunity they transition back to *S*. Healthy adult can become carriers *C*, who do not acquire immunity upon recovery and transition back to *S*. As immunity from the invasive disease does not confer immunity from becoming a carrier, recovered adults can transition to *C*. Carriers spread bacteria in the water source *W* at a rate $\gamma$ and they decay at rate $\xi$.

We developed an age and comorbidity structured model for the transmission of iNTS in sSA. Our model uses a susceptible-infected-recovered framework^21^, where the population is divided in compartments depending on their age and health status, and transitions between compartments over time (Fig. 5 Diagram of the compartmental model). As iNTS transmission is age-dependent^1,22–26^, we divide the population in 4 age groups: 0-6 mos, 7-9 mos, 10-59 mos, $>59$ mos. Individuals in the first age group are assumed to be maternal immune^27–29^. Consistent with the evidence that iNTS transmission depends on the presence of comorbidities^2,28,30–35^ each age group is stratified according to their comorbidity status. For children up to 59 mos we consider both Malaria and HIV, while after 59 mos we only consider immunocompromised as at high risk of developing iNTS disease^28,36^. Carriers have an important role in the transmission of iNTS as they contribute to spread the bacteria despite not being affected by the invasive disease^37–40^. We assume that only healthy individuals above 59 mos can become carriers, while children 7-59 mos (irrespective of their comorbidity status), and those older than 59 mos with HIV can get the invasive disease upon exposure^31,41–43^. Model equations and parameters can be found in Supplementary Methods 1 and Supplementary Table 2.

### Data and calibration

Population pyramids and population growth rates are taken from the United Nations Population Division^44^. We consider the medium fertility variant (median prediction interval) projections for the population growth. As population pyramids are structured in one year intervals, we use Sprague multipliers to estimate population pyramids structured in four age groups. Malaria reported confirmed cases are obtained from WHO Global Health Observatory Data Repository^45^, and the number of children 0-14 y.o. and adults 15-49 y.o. living with HIV is based on UNAIDS estimates^46^. Comorbidity data refer to 2016, except a few cases where 2016 data was not available, for which we took the closest year available. For countries where data was not available we assumed they had the same comorbidity level of a neighbouring country (for Eswatini we assumed the same comorbidity as South Africa and for Djibouti the same as Ethiopia).

UN estimates reports the number of children 0-14 y.o. living with HIV, to compute the percentages of children living with HIV in age group 0-6 months, 7-9 months and 10-59 months, we assumed that the number of children living with HIV is uniformly distributed over age groups in the interval 0-14 years of age. Similarly we assume that Malaria cases are uniformly distributed among different age groups. There is evidence that the distribution of clinical malaria cases is relatively evenly distributed among the first 10 years of life^47^, but this likely underestimates malaria cases among children younger than 5 years. This implies that we are likely underestimating the cases of iNTS in our model among children up to 59 months. For the routine vaccination scenario we use the WHO-UNICEF estimation of DTP1 vaccine coverage for each country, choosing as reference year 2019^48^. For the catch-up vaccination scenario we use the same DTP1 coverage if this is greater than $90\%$, otherwise we assume a $90\%$ coverage for the one-year campaign. We interpret this as a reference best case scenario, as mass campaigns are likely to be below this threshold for countries that do not reach 90% coverage for DTP1. Coverage data is summarised in Supplementary Table 1.

We calibrate the age and comorbidity specific transmission parameters to reach a yearly prevalence level of 0.3$\%$ among children age 0-5 years for the aggregate sub-Saharan Africa, using maximum likelihood estimation with normally distributed error. We then simulate the transmission model for each country, using country specific population pyramid, comorbidities and vaccine coverage. We assume that transmission rate in presence of comorbidities is 3.5 times the transmission rate without comorbidities for that age group. We estimate the rate of progression among age groups using mortality rates and annual population growth rate.

### Intervention simulations

We simulate a one-year catch-up vaccination campaign among children between 9 months old and 5 years old, followed by a routine vaccination campaign at 9 months of age. We assume that routine iNTS vaccination follows the EPI schedule set by WHO, with the first vaccination either at week 6, 10 or 14 and the second vaccination at 9 months. For simplicity we assume that the first vaccination does not induce any immunity, so we can model only the second at 9 months. We simulate vaccine efficacy between 85$\%$ and 95$\%$. We assume no waning of vaccine induced immunity as well as no protection against carriage, as currently there is no evidence regarding these two aspects. Furthermore, we assume that vaccine efficacy is not affected by Malaria or HIV status of the recipient.

## Discussion

Without any health intervention in sSA our model calculated that the annual number of cases in children below 5 years of age will grow, resulting in an estimate cumulative number of cases in children below 5 years of age of 9.7 million by 2038. This huge number is partly due to the expected growth of the population below 5 years in sSA, this age group being the most affected by the disease. Vaccines are one of the most successful public health initiatives in eliminating or reducing the impact of infectious diseases^49^, having a great impact on human health and contributing to increase life expectancy and quality^50,51^. Our model computed the impact of different immunization strategies considering different levels of vaccine efficacy. We also show that in evaluating the impact of a large scale vaccination campaign against iNTS is of key importance to focus on a country-level analysis (Supplementary Figs. 9–57). Our analyses indicate that vaccination of children below 5 years of age could effectively and efficiently reduce iNTS burden in sSA. Different levels of vaccine coverage and difference in comorbidities are the main factors behind differences in the reduction of cases following vaccination, as these are the two main factors of variation in the model (together with demographic structure). For example South Sudan and Somalia, which have the lowest coverage rate for routine vaccination (51$\%$ and 52$\%$ respectively), show the lowest reduction in cumulate infections (27–30$\%$ and 29–32$\%$ depending on vaccine efficacy). Increasing EPI coverage at 90$\%$ for both countries would reduce iNTS cases by (45–51$\%$) and (47–53$\%$) respectively. Differences in coverage are not the only factor determining reduction in cases following vaccination: consider Burundi and Senegal, they both have a coverage level of 97$\%$ (both for routine and catch-up) but the reduction in cumulate cases significantly less for Burundi [43–52$\%$] than for Senegal [52–60$\%$], difference that can be explained by the much higher number of Malaria cases among children in Burundi. This stresses the role of comorbidities in the diffusion of iNTS and consequently on the impact of vaccination (Supplemetary Table 3). A few caveats must be raised in interpreting the results of our model. First, by choosing to calibrate our model for the entire sub-Saharan Africa instead of calibrating it for each country, we are losing some of the country-specific characteristics in the transmission dynamics for the sake of reducing the variance of our estimation. We are letting only comorbidities and the age pyramid to vary across country, and we are not taking into account several country-specific factors that are likely to affect transmission. For example we are not considering differences in the interaction patterns among difference age groups, nor we are taking into account differences in water sanitation levels which can have a significant impact on the diffusion of the disease as better water sanitation leads to reduced bacteria circulation. A further caveat to raise is the fact that our assumption on the distribution of malaria cases likely leads to underestimation of malaria cases among children, as discussed previously. This needs to be kept in mind when interpreting our results, as the number of iNTS cases among children below 5 years of age is likely to be higher than our estimate, especially in countries where malaria prevalence is higher, and consequently the reduction in cases in these countries after the vaccination campaign is very likely higher than our estimate. A related issue is that the data on malaria cases we used does not take into account the fact that limited access to care leads to fewer individuals being tested and diagnosed for malaria, so we are likely underestimating malaria cases, especially for countries were access to care is more limited. Moreover our assumption on the magnitude of the scaling factor for transmission in presence of comorbidities should ideally be tested empirically when more data become available. Furthermore the model does not take into account the effects that the COVID-19 pandemic might have had on the diffusion of iNTS in Africa, reducing personal contacts and improving life style, quality of sanitation and hygienic conditions. Although multidrug resistance is becoming a major concern for the epidemiology and treatment of *Salmonella*, possible modifications in the iNTS disease transmission due to the *Salmonella* serovar antibiotic resistant were not taken in consideration in the model, most likely leading to a smaller estimate of the number of cases and deaths related to iNTS. Similarly, we also assumed no vaccine waning and no protection against carriage: currently information on the human responses to the vaccination are limited by availability, since the vaccine under study has been just launched in the clinical phases and study are currently in progress. In conclusion we evaluate the iNTS diffusion in sSA, by country and age class, considering endemic comorbidities, and highlight that the transmission of the iNTS disease among the most fragile age classes below 5 years of age in sSA will be increasing over the next 20 years without the introduction of a iNTS vaccine. By the simulation of different vaccination scenarios we identified the combination of immunization strategies needed to reduce as early as possible the burden of the iNTS disease reducing permanently its incidence. Strategies to address the iNTS burden in sSA include infection prevention, optimised use of antibiotics, improved capacity for microbiological analysis, and vaccine development. Our estimates can be used to help set priorities for vaccine need, demand and development. Overall, our findings bring to the conclusion that until safer sources of water and sanitization will not be widespread distributed and available for all population of sSA, number of cases and deaths will be increasing without the introduction of a vaccine. Vaccination against iNTS, being the faster prevention method, will be highly beneficial and should be prioritized as primary health intervention. Moreover, from a broader perspective our model offers a very useful tool to monitor the development of iNTS disease, to select the immunization strategies and to identify priority countries for vaccine introduction to adequately address iNTS disease. It also provides estimates on the impact of vaccine introduction at the country level and help us prioritize countries with high expected return on introduction of vaccines given the present prevalence of the disease and current vaccination coverage.

## Supplementary Information

Supplementary Information.

## Data Availability

Country specific data are publicly available and their sources are specified in 3.2. Moreover the population pyramids and the age-group specific comorbidity data extracted from these sources are also available at https://github.com/danielecassese/Effectiveness-of-iNTS-vaccination.git. Python codes for simulating iNTS dynamics and vaccine impact are available at https://github.com/danielecassese/Effectiveness-of-iNTS-vaccination.git.

## References

[1] Stanaway, J. D. et al. The global burden of non-typhoidal salmonella invasive disease: a systematic analysis for the global burden of disease study 2017. *Lancet. Infect. Dis* **19**(12), 1312–1324. 10.1016/S1473-3099(19)30418-9 (2019). 31562022 10.1016/S1473-3099(19)30418-9 PMC6892270

[2] Marchello, C. S. et al. Complications and mortality of non-typhoidal salmonella invasive disease: a global systematic review and meta-analysis. *Lancet Infect. Dis.* **22**(5), 692–705. 10.1016/S1473-3099(21)00615-0 (2022). 35114140 10.1016/S1473-3099(21)00615-0 PMC9021030

[3] Ikuta, K. S. et al. Global mortality associated with 33 bacterial pathogens in 2019: a systematic analysis for the global burden of disease study 2019. *Lancet*10.1016/S0140-6736(22)02185-7 (2022). 36423648 10.1016/S0140-6736(22)02185-7 PMC9763654

[4] Marchello, C. S. et al. Incidence of non-typhoidal salmonella invasive disease: A systematic review and meta-analysis. *J. Infect.* **83**(5), 523–532. 10.1016/j.jinf.2021.06.029 (2021). 34260964 10.1016/j.jinf.2021.06.029 PMC8627500

[5] Van Puyvelde, S. et al. An African salmonella typhimurium st313 sublineage with extensive drug-resistance and signatures of host adaptation. *Nat. Commun.* **10**(1), 4280. 10.1038/s41467-019-11844-z (2019). 31537784 10.1038/s41467-019-11844-z PMC6753159

[6] Kingsley, R. A. et al. Epidemic multiple drug resistant salmonella typhimurium causing invasive disease in sub-Saharan Africa have a distinct genotype. *Genome Res.* **19**(12), 2279–87. 10.1101/gr.091017.109 (2009). 19901036 10.1101/gr.091017.109 PMC2792184

[7] Park, S. E. et al. The genomic epidemiology of multi-drug resistant invasive non-typhoidal salmonella in selected sub-Saharan African countries. *BMJ Glob. Health*10.1136/bmjgh-2021-005659 (2021). 34341020 10.1136/bmjgh-2021-005659 PMC8330565

[8] Akullian, A. et al. Multi-drug resistant non-typhoidal salmonella associated with invasive disease in western Kenya. *PLoS Negl. Trop. Dis.* **12**(1), 1–16. 10.1371/journal.pntd.0006156 (2018). 10.1371/journal.pntd.0006156 PMC5785031 29329299

[9] Gordon, M. A. et al. Epidemics of invasive salmonella enterica serovar enteritidis and S. enterica serovar typhimurium infection associated with multidrug resistance among adults and children in Malawi. *Clin. Infect. Dis.* **46**(7), 963–69. 10.1086/529146 (2008). 18444810 10.1086/529146

[10] Uche, V. I., MacLennan, C. A. & Saul, A. A systematic review of the incidence, risk factors and case fatality rates of invasive nontyphoidal salmonella (iNTS) disease in Africa (1966 to 2014). *PLoS Negl. Trop. Dis.* **11**(1), 1–28 (2017). 10.1371/journal.pntd.0005118 PMC5215826 28056035

[11] Marks, F. et al. Incidence of invasive Salmonella disease in sub-Saharan Africa: a multicentre population-based surveillance study. *Lancet Glob. Health* **5**(3), e310–e323 (2017). 28193398 10.1016/S2214-109X(17)30022-0 PMC5316558

[12] Crump, J. A. et al. A perspective on invasive salmonella disease in Africa. *Clin. Infect. Dis.* **61**(4), 235–40. 10.1093/cid/civ709 (2015). 10.1093/cid/civ709 PMC4596931 26449937

[13] Feasey, N. A. et al. Three epidemics of invasive multidrug-resistant salmonella bloodstream infection in Blantyre, Malawi, 1998–2014. *Clin. Infect. Dis.* **61**(4), 363–71 (2015). 10.1093/cid/civ691 PMC4596930 26449953

[14] Kalonji, L. M. et al. Invasive salmonella infections at multiple surveillance sites in the Democratic Republic of the Congo, 2011–2014. *Clin. Infect. Dis.* **61**(4), 363–71 (2015). 10.1093/cid/civ713 26449951

[15] Kariuki, S. et al. Multidrug-resistant nontyphoidal salmonella hotspots as targets for vaccine use in management of infections in endemic settings. *Clin. Infect. Dis.* **68**(1), 10–15 (2019). 10.1093/cid/ciy898 PMC6376148 30767004

[16] Kariuki, S. et al. Antimicrobial resistance and management of invasive salmonella disease. *Vaccine* **33**, 21–29. 10.1016/j.vaccine.2015.03.102 (2015). 10.1016/j.vaccine.2015.03.102 PMC4469558 25912288

[17] Tacconelli, E. et al. Discovery, research, and development of new antibiotics: the who priority list of antibiotic-resistant bacteria and tuberculosis. *Lancet. Infect. Dis* **18**(3), 318–327. 10.1016/S1473-3099(17)30753-3 (2018). 29276051 10.1016/S1473-3099(17)30753-3

[18] Tennant, S. M., MacLennan, C. A., Simon, R., Martin, L. B. & Khan, M. I. Nontyphoidal salmonella disease: Current status of vaccine research and development. *Vaccine* **34**(26), 2907–2910 (2016). 27032517 10.1016/j.vaccine.2016.03.072

[19] Baliban, S. M., Lu, Y.-J. & Malley, R. Overview of the nontyphoidal and paratyphoidal salmonella vaccine pipeline: current status and future prospects. *Clin. Infect. Dis.* **71**, 151–154 (2020). 10.1093/cid/ciaa514 PMC7388718 32725233

[20] Piccini, G. & Montomoli, E. Pathogenic signature of invasive non-typhoidal salmonella in Africa: implications for vaccine development. *Hum. Vaccines Immunother.* **16**(9), 2056–2071. 10.1080/21645515.2020.1785791 (2020). 10.1080/21645515.2020.1785791 PMC7553687 32692622

[21] Keeling, M. J. & Rohani, P. *Modeling Infectious Diseases in Humans and Animals* (Princeton University Press, 2011). 10.1515/9781400841035.

[22] Feasey, N. A. et al. Typhoid fever and invasive nontyphoid salmonellosis, Malawi and South Africa. *Emerg. Infect. Dis.* **16**(9), 1448–51. 10.3201/eid1609.100125 (2010). 20735930 10.3201/eid1609.100125 PMC3294972

[23] Ao, T. T. et al. Global burden of invasive nontyphoidal salmonella disease, 2010. *Emerg. Infect. Dis.* **21**(6), 941–9. 10.3201/eid2106.140999 (2015). 25860298 10.3201/eid2106.140999 PMC4451910

[24] Balasubramanian, R. et al. The global burden and epidemiology of invasive non-typhoidal salmonella infections. *Hum. Vaccin. Immunother.* **15**(6), 1421–1426. 10.1080/21645515.2018.1504717 (2019). 30081708 10.1080/21645515.2018.1504717 PMC6663144

[25] Keddy, K. H. et al. Clinical and microbiological features of invasive nontyphoidal salmonella associated with HIV-infected patients, Gauteng Province, South Africa. *Medicine*10.1097/MD.0000000000006448 (2017). 28353576 10.1097/MD.0000000000006448 PMC5380260

[26] Bornstein, K. et al. Modeling the potential for vaccination to diminish the burden of invasive non-typhoidal salmonella disease in young children in Mali, West Africa. *PLoS Negl. Trop. Dis.* **11**(2), 1–19. 10.1371/journal.pntd.0005283 (2017). 10.1371/journal.pntd.0005283 PMC5300129 28182657

[27] MacLennan, C. A. et al. The neglected role of antibody in protection against bacteremia caused by nontyphoidal strains of salmonella in African children. *J. Clin. Investig.* **118**(4), 1553–62. 10.1172/JCI33998 (2008). 18357343 10.1172/JCI33998 PMC2268878

[28] Feasey, N. A. et al. Invasive non-typhoidal salmonella disease: an emerging and neglected tropical disease in Africa. *Lancet* **379**(9835), 2489–2499. 10.1016/S0140-6736(11)61752-2 (2012). 22587967 10.1016/S0140-6736(11)61752-2 PMC3402672

[29] Lepage, P. et al. Severe multiresistant Salmonella typhimurium systemic infections in Central Africa ? clinical features and treatment in a paediatric department. *J. Antimicrob. Chemother.* **14**(supplB), 153–159. 10.1093/jac/14.supplB.153 (1984). 6094435 10.1093/jac/14.suppl_b.153

[30] Park, S. E. et al. The relationship between invasive nontyphoidal salmonella disease, other bacterial bloodstream infections, and malaria in sub-Saharan Africa. *Clin. Infect. Dis.* **62**(suppl1), 23–31. 10.1093/cid/civ893 (2016). 10.1093/cid/civ893 PMC4772835 26933016

[31] Muthumbi, E. et al. Invasive Salmonellosis in Kilifi, Kenya. *Clin. Infect. Dis.* **61**(suppl4), 290–301. 10.1093/cid/civ737 (2015). 10.1093/cid/civ737 PMC4596936 26449944

[32] Scott, J. A. G. et al. Relation between falciparum malaria and bacteraemia in Kenyan children: a population-based, case-control study and a longitudinal study. *Lancet* **378**(9799), 1316–1323. 10.1016/S0140-6736(11)60888-X (2011). 21903251 10.1016/S0140-6736(11)60888-X PMC3192903

[33] Gordon, M. A. Invasive nontyphoidal salmonella disease: epidemiology, pathogenesis and diagnosis. *Curr. Opin. Infect. Dis.* **24**(5), 484–489 (2011). 21844803 10.1097/QCO.0b013e32834a9980 PMC3277940

[34] Greenwood, B. M. et al. Immunosuppression in children with malaria. *Lancet* **299**(7743), 169–172. 10.1016/S0140-6736(72)90569-7 (1972). 10.1016/s0140-6736(72)90569-7 4109547

[35] MacLennan, C. A. et al. Dysregulated humoral immunity to nontyphoidal ‘salmonella’ in HIV-infected African adults. *Science* **328**(5977), 508–512. 10.1126/science.1180346 (2010). 20413503 10.1126/science.1180346 PMC3772309

[36] Crump, J. A. et al. Epidemiology, clinical presentation, laboratory diagnosis, antimicrobial resistance, and antimicrobial management of invasive salmonella infections. *Clin. Microbiol. Rev.* **28**(4), 901–937. 10.1128/CMR.00002-15 (2015). 26180063 10.1128/CMR.00002-15 PMC4503790

[37] Post, A. S. et al. Supporting evidence for a human reservoir of invasive non-typhoidal salmonella from household samples in Burkina Faso. *PLoS Negl. Trop. Dis.* **13**(10), 1–18. 10.1371/journal.pntd.0007782 (2019). 10.1371/journal.pntd.0007782 PMC6812844 31609964

[38] Kariuki, S. et al. Invasive multidrug-resistant non-typhoidal salmonella infections in Africa: zoonotic or anthroponotic transmission?. *J. Med. Microbiol.* **55**(5), 585–591. 10.1099/jmm.0.46375-0 (2006). 16585646 10.1099/jmm.0.46375-0

[39] Koolman, L. et al. Case-control investigation of invasive salmonella disease in africa reveals no evidence of environmental or animal reservoirs of invasive strains. *medRxiv*. 10.1101/2022.01.31.22270114 (2022). 10.1371/journal.pntd.0010982 PMC9779717 36508466

[40] Parsons, B. N. et al. Invasive non-typhoidal salmonella typhimurium st313 are not host-restricted and have an invasive phenotype in experimentally infected chickens. *PLoS Negl. Trop. Dis.* **7**(10), 1–8. 10.1371/journal.pntd.0002487 (2013). 10.1371/journal.pntd.0002487 PMC3794976 24130915

[41] Levine, W. C. et al. Epidemiology of nontyphoidal salmonella bacteremia during the human immunodeficiency virus epidemic. *J. Infect. Dis.* **164**(1), 81–87. 10.1093/infdis/164.1.81 (1991). 2056220 10.1093/infdis/164.1.81

[42] Gordon, M. A. et al. Bacteraemia and mortality among adult medical admissions in Malawi - predominance of non-typhi salmonellae and streptococcus pneumoniae. *J. Infect.* **42**(1), 44–49. 10.1053/jinf.2000.0779 (2001). 11243753 10.1053/jinf.2000.0779

[43] Gordon, M. A. et al. Non-typhoidal salmonella bacteraemia among HIV-infected Malawian adults: high mortality and frequent recrudescence. *AIDS* **16**(12), 1633–1641 (2002). 12172085 10.1097/00002030-200208160-00009

[44] Nations, U.: World Population Prospects 2019. Accessed October 2021 (2020). https://population.un.org/wpp/Download/Standard/Population/

[45] WHO: Global Health Observatory. Accessed June 2020 (2016). https://apps.who.int/gho/data/node.main.A1364?lang=en

[46] UNAIDS: UNAIDS Countries. Accessed January 2018 (2016). https://www.unaids.org/en/regionscountries/countries

[47] Carneiro, I. et al. Age-patterns of malaria vary with severity, transmission intensity and seasonality in sub-Saharan Africa: A systematic review and pooled analysis. *PLoS ONE* **5**(2), 1–10. 10.1371/journal.pone.0008988 (2010). 10.1371/journal.pone.0008988 PMC2813874 20126547

[48] WHO-UNICEF: WHO-UNICEF estimates of DPT1 coverage. Accessed October 2021 (2019). https://apps.who.int/immunization_monitoring/globalsummary/timeseries/tswucoveragedtp1.html

[49] Timmis, J. K., Rigat, F. & Rappuoli, R. Core values for vaccine evaluation. *Vaccine* **35**(1), 57–62. 10.1016/j.vaccine.2016.11.034 (2017). 10.1016/j.vaccine.2016.11.034 28017445

[50] WHO: Global vaccine action plan. (World Health Organization, 2013).

[51] Black, S. The role of health economic analyses in vaccine decision making. *Vaccine* **31**(51), 6046–6049. 10.1016/j.vaccine.2013.08.008 (2013). 23968768 10.1016/j.vaccine.2013.08.008

