# Supplementary material for: Effectiveness of iNTS vaccination in sub-Saharan Africa
Source: Sci Rep. 2025 Jan 30;15:3765. doi: 10.1038/s41598-025-87659-4 (PMC11782671; doi:10.1038/s41598-025-87659-4)
Supplement: Supplementary file 1 — Supplementary Information. [file 41598_2025_87659_MOESM1_ESM.pdf]

Supporting Information
Effectiveness of iNTS vaccination in sub-Saharan Africa

Daniele Cassese[a,*], Nicola Dimitri[b], Gianluca Breghi[c], and Tiziana Spadafina[c,d,*]

[a]University of Cambridge, Cambridge, CB2 3AP
[b]Department of Economics, University of Siena, Siena, 53100
[c]Fondazione Achille Sclavo ONLUS, Siena 53100
[d]Sclavo Vaccines Association E.T.S., Siena 53100
[*]To whom correspondence should be addressed. E-mails: dc554@cam.ac.uk,spadafina@sclavo.org

This file contains:

- Supplementary Methods 1-2
- Supplementary Results 1-2
- Supplementary Figs. 1-57
- Supplementary Tables 1-5

Supplementary Methods 1. Model specification

The model stratifies the population of each country in 4 age groups: (1) 0-6 months, (2) 7-9 months, (3) 10-59 months, (4) $>$ 59 months and 5 compartments according to the disease

status: maternal immune (M), susceptible (S), infected by the invasive disease (I), carrier of the disease (C) and recovered (R). All compartments except carriers are further stratified according to comorbidity status. Carriers are individuals who can get infected upon exposure and contribute to spread the bacteria but who will not get the invasive disease [1, 2, 7]. We assume that only individuals older than 59 months without comorbidities can be carriers, while individuals in the first three age groups, both with and without comorbidities, and individuals older than 59 months with HIV can get the invasive disease upon exposure. This is consistent with evidence that children are the most at risk to get iNTS.

We do not model the transmission dynamics of comorbidities (Malaria and HIV), instead we estimate the fraction of people with comorbidities in each age group and we assume it constant. Individuals are born maternally immune, so they enter the model in compartment M where they stay for their first 6 months of life, then transition into the susceptible compartment. The susceptible compartment $S_{a,m}$ is stratified in age groups $a = \{2, 3, 4\}$ (first age group not included because of maternal immunity) and in comorbidity status $m = \{n, c\}$, where n stands for *no comorbidity* and c for *with comorbidity*. Susceptible individuals below 5 years old (both with or without comorbidities) and above 5 years old with comorbidities can become infected with force of infection $\lambda_{a,m}$ depending on their age group and comorbidity status. Once infected, individuals remain in the corresponding infected compartment $I_{a,m}$ for 14 days, then transition into the recovered compartment $R_{a,m}$. Following natural immunity waning, individuals will transition from $R_{a,m}$ to the susceptible compartment $S_{a,m}$ at a rate ϕ. Susceptible individuals older than 5 years of age and without comorbidities will transition to the carrier compartment $C_{4,n}$ at a rate $\lambda_{4,n}$. We assume that infected individuals do not transmit the infection while carrier individuals both transmit the infection and shed the bacteria at a rate γ in the water supply. We allow both recovered and vaccinated individuals, with and without comorbidities, to become carriers upon exposure, which corresponds to assuming that both natural immunity and vaccine induced immunity protects from the invasive disease, but do not prevent becoming carrier. The demographic of the population is modelled letting individual transition within their compartment from age group i to age group $i + 1$ at rate $K_{i,m}$ and die at rate $d_{i,m}$ where m is comorbidity status, such that total population, age structure and comorbidity status remain constant [4]. The transition between compartments is captured by a system of differential equations, that in the case without vaccination read as follows (where $\dot{x} = \frac{dx}{dt}$):

$$\begin{aligned}
\dot{M}_{1,m} &= q_m - (d_{1,m} + K_{1,m})M_{1,m} \\
\dot{S}_{2,m} &= K_{1,m}M_{1,m} + \phi R_{2,m} - (\lambda_{2,m} + d_{2,m} + K_{2,m})S_{2,m} \\
\dot{S}_{3,m} &= K_{2,m}S_{2,m} + \phi R_{3,m} - (\lambda_{3,m} + d_{3,m} + K_{3,m})S_{3,m} \\
\dot{S}_{4,n} &= K_{3,n}S_{3,n} + \phi R_{4,n} + \psi CS_{4,n} - (\lambda_{4,n} + d_{4,n})S_{4,n} \\
\dot{S}_{4,c} &= K_{3,c}S_{3,c} + \phi R_{4,c} - (\lambda_{4,c} + d_{4,c})S_{4,c} \\
\dot{I}_{2,m} &= \lambda_{2,m}S_{2,m} - (\rho + d_{2,m} + K_{2,m})I_{2,m} \\
\dot{I}_{3,m} &= K_{2,m}I_{2,m} + \lambda_{3,m}S_{3,m} - (\rho + d_{3,m} + K_{3,m})I_{3,m} \\
\dot{I}_{4,n} &= K_{3,n}I_{3,n} - (\rho + d_{4,n})I_{4,n} \\
\dot{I}_{4,c} &= K_{3,c}I_{3,c} + \lambda_{4,c}S_{4,c} - (\rho + d_{4,c})I_{4,c} \\
\dot{R}_{2,m} &= \rho I_{2,m} - (\phi + d_{2,m} + K_{2,m})R_{2,m} \\
\dot{R}_{3,m} &= K_{2,m}R_{2,m} + \rho I_{2,m} - (\phi + d_{2,m} + K_{2,m})R_{2,m} \\
\dot{R}_{4,m} &= K_{3,m}R_{3,m} + \rho I_{4,m} + \psi CR_{4,m} - (\phi + d_{4,m} + \lambda_{4,m})R_{4,m} \\
\dot{CS}_{4,n} &= \lambda_{4,n}S_{4,n} - (d_{4,n} + \psi)CS_{4,n} \\
\dot{CR}_{4,m} &= \lambda_{4,m}R_{4,m} - (a_{4,m} + d_{4,m} + \psi)CR_{4,m} \\
\dot{W} &= \gamma(CS_{4,n} + \sum_m CR_{4,m}) - \xi W
\end{aligned} \tag{1}$$

W represents the contamination of the water supply due to the shedding of carriers, q_m is birth rate for comorbidity status (we are assuming that individuals are born with or without comorbidities, as we do not explicitly model the dynamics of Malaria and HIV), $d_{a,m}$ are age and comorbidity specific death rates, $K_{a,m}$ transition rates between age classes, $\rho_{a,m}$ the natural recovery rate, ϕ rate of natural immunity loss. Finally ξ is the rate at which bacteria are eliminated from the water supply.

The force of infection for iNTS depends on seasonality, age and comorbidity status according to the equation:

$$\lambda_{a,m} = \beta_{a,m}\Big[CS_{4,n} + \sum_m CR_{4,m} + \Big(1 + f\cos\Big(\frac{g\pi}{365}(t-p)\Big)\Big)W\Big] \tag{2}$$

Where CS and CR are adult susceptible and recovered individuals respectively who became carriers. The transmission rates $\beta_{a,m}$ capture that susceptibility differs because of age and comorbidities, and the term in brackets before W captures seasonal variation: g is a cosine scaling, p is a seasonal offset and f the seasonal amplitude. Note that we assume homogeneous mixing between age groups.

In the model with vaccination the population is further stratified to include vaccine related compartments. In our simulation scenario we vaccinate either at 9 months (in the routine scenario), or between 9 months and 5 years (in the catch-up scenario), hence both the maternally immune M and carrier C do not have their corresponding vaccinated compartment, even if we do keep track of vaccinated susceptibles and vaccinated recovered that become carriers. **Supplementary Fig. 1 Model Compartments** shows the compartments and the transition between them.

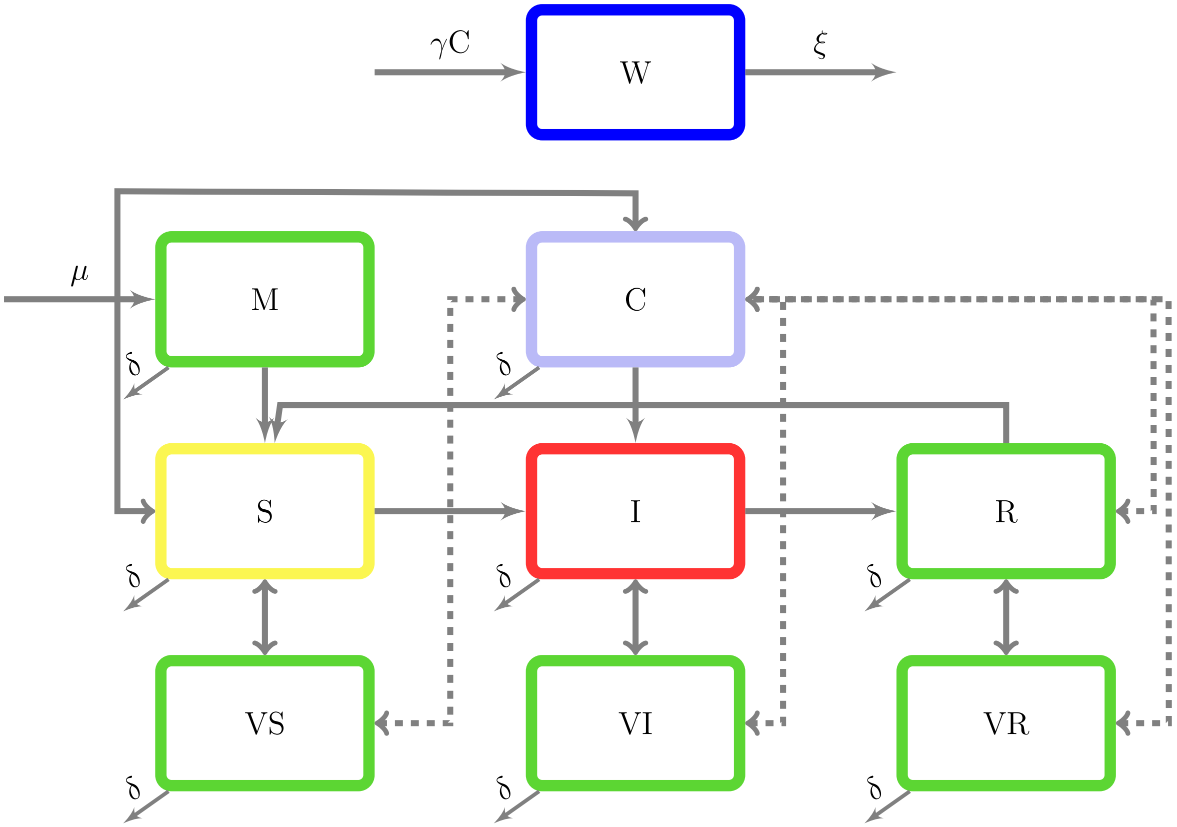

Supplementary Fig. 1 Model Compartments: Scheme of the compartmental model (age structure and comorbidities not shown) with vaccination. The green boxes (Maternal Immune, Recovered and Vaccinated) identify groups that are immune to the invasive disease. Notice that we assume that neither the vaccine nor immunity after recovering prevent from becoming carrier if exposed to the bacteria. (The lines are dashed as only adults can become carriers).

When we introduce vaccination, the transmission model is governed by the following set of differential equations:

$$
\begin{aligned}
\dot{M}_{1,m} &= q_m - (d_{1,m} + K_{1,m})M_{1,m} \\
\dot{S}_{2,m} &= K_{1,m}M_{1,m} + \phi R_{2,m} - (\lambda_{2,m} + d_{2,m} + K_{2,m})S_{2,m} \\
\dot{S}_{3,m} &= K_{2,m}S_{2,m} + \phi R_{3,m} - (\lambda_{3,m} + d_{3,m} + K_{3,m} - v)S_{3,m} \\
\dot{S}_{4,n} &= K_{3,n}S_{3,n} + \phi R_{4,n} + \psi CS_{4,n} - (\lambda_{4,n} + d_{4,n})S_{4,n} \\
\dot{S}_{4,c} &= K_{3,c}S_{3,c} + \phi R_{4,c} - (\lambda_{4,c} + d_{4,c})S_{4,c} \\
\dot{I}_{2,m} &= \lambda_{2,m}S_{2,m} - (\rho + d_{2,m} + K_{2,m})I_{2,m} \\
\dot{I}_{3,m} &= K_{2,m}I_{2,m} + \lambda_{3,m}S_{3,m} - (\rho + d_{3,m} + K_{3,m} - v)I_{3,m} \\
\dot{I}_{4,n} &= K_{3,n}I_{3,n} - (\rho + d_{4,n})I_{4,n} \\
\dot{I}_{4,c} &= K_{3,c}I_{3,c} + \lambda_{4,c}S_{4,c} - (\rho + d_{4,c})I_{4,c} \\
\dot{R}_{2,m} &= \rho I_{2,m} - (\phi + d_{2,m} + K_{2,m})R_{2,m} \\
\dot{R}_{3,m} &= K_{2,m}R_{2,m} + \rho I_{2,m} - (\phi + d_{2,m} + K_{2,m} - v)R_{2,m} \\
\dot{R}_{4,m} &= K_{3,m}R_{3,m} + \rho I_{4,m} + \psi CR_{4,m} - (\phi + d_{4,m} + \lambda_{4,m})R_{4,m} \\
\dot{CS}_{4,n} &= \lambda_{4,n}S_{4,n} - (d_{4,n} + \psi)CS_{4,n} \\
\dot{CR}_{4,m} &= \lambda_{4,m}R_{4,m} - (a_{4,m} + d_{4,m} + \psi)CR_{4,m} \\
\dot{CVS}_{4,m} &= \lambda_{4,n}VS_{4,m} - (d_{4,m} + \psi)CVS_{4,m} \\
\dot{CVR}_{4,m} &= \lambda_{4,m}VR_{4,m} - (a_{4,m} + d_{4,m} + \psi)CVR_{4,m} \\
\dot{VS}_{3,m} &= vS_{3,m} - (d_{3,m} + \lambda_{3,m})VS_{3,m} \\
\dot{VS}_{4,m} &= K_{3,m}VS_{3,m} - (d_{4,m} + \lambda_{4,m})VS_{4,m} \\
\dot{VR}_{3,m} &= vR_{3,m} - (d_{3,m} + \lambda_{3,m})VR_{3,m} \\
\dot{VR}_{4,m} &= K_{3,m}VR_{3,m} - (d_{4,m} + \lambda_{4,m})VR_{4,m} \\
\dot{W} &= \gamma(CS_{4,n} + \sum_m CR_{4,m} + \sum_m CVS_{4,m} + \sum_m CVR_{4,m}) - \xi W
\end{aligned} \tag{3}
$$

where v captures country-specific coverage rates. CVS and CVR are adult vaccinated susceptible (recovered) individuals who become carriers: they are immune to the invasive disease but not to carriage. VS and VR are vaccinated susceptible and recovered individuals respectively. We model two vaccination scenarios, routine and catch-up. In the routine scenario we assume that iNTS vaccination follows the EPI schedule set by WHO, with the first vaccination either at week 6, 10 or 14 and the second vaccination at 9 months. For

simplicity we assume that the first vaccination does not induce any immunity, so it is only the second dose at 9 months gives coverage: as soon as individuals enter age group 3 they are vaccinated, and so removed from the susceptible compartment. For the catch-up scenario, we assumed one dose only, vaccinating children below 5 years of age.

Supplementary Table 1 Vaccine Coverage (DTP1): Country specific vaccine coverage for routine (DTP1) and catch-up.

Country	Coverage DTP1	Coverage catch-up	Country	Coverage DTP1	Coverage catch-up
Angola	67	90	Madagascar	85	90
Benin	84	90	Malawi	97	97
Botswana	98	98	Mali	82	90
Burkina Faso	95	95	Mauritania	89	90
Burundi	97	97	Mauritius	97	97
Cabo Verde	96	96	Mayotte	90	90
Cameroon	75	90	Mozambique	93	93
CAR	69	90	Namibia	92	92
Chad	65	90	Niger	92	92
Comoros	96	96	Réunion	90	90
Congo	82	90	Nigeria	65	90
Côte d'Ivoire	98	98	Rwanda	99	99
DRC	66	90	Sao Tome	97	97
Djibouti	90	90	Senegal	97	97
Equatorial Guinea	77	90	Seychelles	99	99
Eritrea	97	97	Sierra Leone	95	95
Eswatini	96	96	Somalia	52	90
Ethiopia	80	90	South Africa	84	90
Gabon	77	90	South Sudan	51	90
Gambia	93	93	Togo	90	90
Ghana	97	97	Uganda	99	99
Guinea	62	90	UR of Tanzania	91	91
Guinea-Bissau	85	90	Zambia	94	94
Kenya	97	97	Zimbabwe	94	94
Lesotho	92	92			
Liberia	94	94	SUB-SAHARAN AFRICA	92	92

Supplementary Table 2 Model Parameters: Parameters choice and their justification.

**Description**	**Value**	**Justification**
Transmission rate $0-6$ months, no comorbidity	$\beta_{1,n} = 0$	Assumption
Transmission rate $0-6$ months, comorbidity	$\beta_{1,c} = 0$	Assumption
Transmission rate $7-9$ months, no comorbidity	$\beta_{2,n} = 0.00022$	Calibrated
Transmission rate $7-9$ months, comorbidity	$\beta_{2,c} = 0.00077$	Assumption
Transmission rate $10-59$ months, no comorbidity	$\beta_{3,n} = 0.00022$	Calibrated
Transmission rate $10-59$ months, comorbidity	$\beta_{3,c} = 0.00077$	Assumption
Transmission rate > 59 months, no comorbidity	$\beta_{4,n} = 0.0082$	Calibrated
Transmission rate > 59 months, comorbidity	$\beta_{4,c} = 0.0290$	Assumption
Duration of infection for Carrier	$\psi = 20$ days	Assumption
Duration of invasive infection, all ages	$1/\rho = 14$ days	Assumption
Duration of natural immunity	$1/\phi = 7$ years	Assumption
Waning rate of vaccine induced immunity	No waning	Assumption
Survival time of bacteria in water	$1/\xi = 50$ days	[6]
Rate of shedding of bacteria into water supply	$\gamma = 1$ infectious unit p.w.	Assumption
Birth rate	$q = 0.0363$ yearly	Data [9]
Peak of seasonal forcing	$1/p = 34$ weeks	Assumption
Seasonal forcing	$f = 0.1$	Assumption
Cos scaling	$g = 2.1$	Assumption
Rate of progression between age groups	Age-specific	Calibrated

Supplementary Methods 2. Sensitivity Analysis

To assess the impact of the main parameters on the model output (the yearly number of infected children below 5 y.o.) we conducted global sensitivity analysis using two methods: the extended Fourier amplitude sensitivity test (eFAST) [8], which decomposes the variance of the output into the contribution of each parameter, and the partial rank correlation coefficient (PRCC) [5], a sampling method that measures the independent effect of each parameter on the number of cases. We implement eFAST sensitivity analysis using SALib library [3], generating parameters' samples using a revised Saltelli sampling [8]. We run 16.366 experiments for the aggregate sub-Saharan Africa and compute the first order index (S1), which captures the contribution to the variance of the output of each parameter averaged over variation of the other parameters and the total order index (ST), which captures the contribution of a parameter and of its interactions with other parameters to the variance of the output. We choose to perform sensitivity analysis on 7 parameters: the transmission rates of children and adult without comorbidities, seasonal forcing, the carrier and infected recovery rates, the waning rate of bacteria from water and the waning rate of natural immunity. Samples are drawn from the uniform distributions over (0.00020,0.00024) for $\beta_{2,n}$, (0.0080,0.00084) for $\beta_{4,n}$, (0.05,0.1) for ρ, (0.0485, 0.055) for ψ, (0.018,0.022) for ξ, (0.00027, 0.00054) for ϕ and (0.088,0.12) for f.

The parameters showing highest first order index are the time it takes for bacteria to decay in the water supply ξ and the below 5 y.o. transmission parameter $\beta_{2,n}$, followed by forcing f and over 5 y.o. transmission parameter $\beta_{4,n}$, with the latter showing slightly larger confidence bounds. Each parameter, when considered together with all others, contributes significantly to the output variance as can be seen by the high total order indices and the narrow confidence bounds.

PRCC helps giving a clearer picture of the role of parameters in the model. The graph in **Supplementary Fig. 2 eFAST sensitivity analysis** shows that the most important parameter in determining the number of infected is $\beta_{2,n}$, while forcing is less important than the eFAST analysis would suggest: this tells us that the high impact of f on the output is explained mostly through its role in directly increasing children infections, as it acts as a rescaling factor for $\beta_{2,n}$, more than through its impact on the number of carriers. This is confirmed by a high interaction between f and $\beta_{2,n}$, and a weak interaction between f and $\beta_{4,n}$ as captured by second order Sobol indices (not reported here). The parameters that follows in order of importance are ϕ and ρ: a shorter duration of natural immunity (higher ϕ) increases the number of paediatric infections and a shorter duration of the infection period

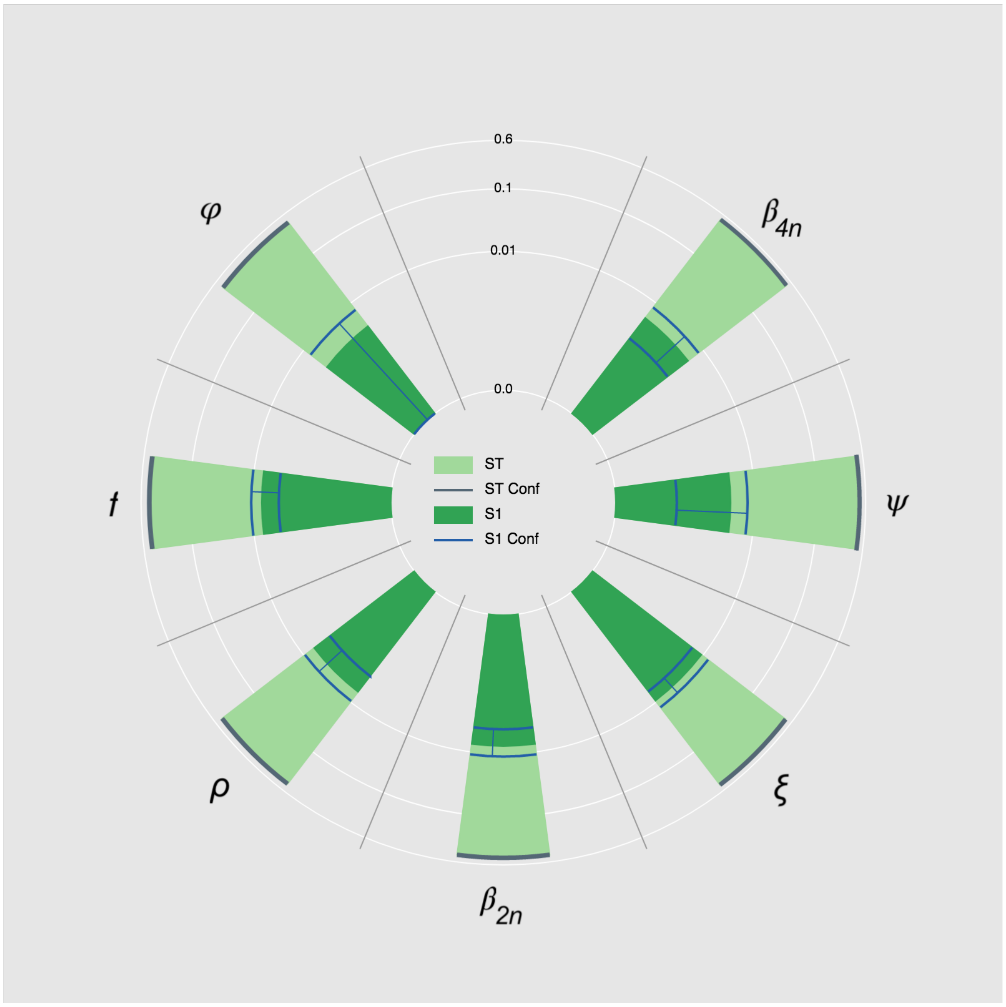

Supplementary Fig. 2 eFAST sensitivity analysis: eFAST sensitivity analysis: S1 captures the contribution of each parameter alone averaged over variation of other input parameters, ST captures the contribution of each parameter including the effect that its interaction has with all the other parameters. Confidence intervals at 95%

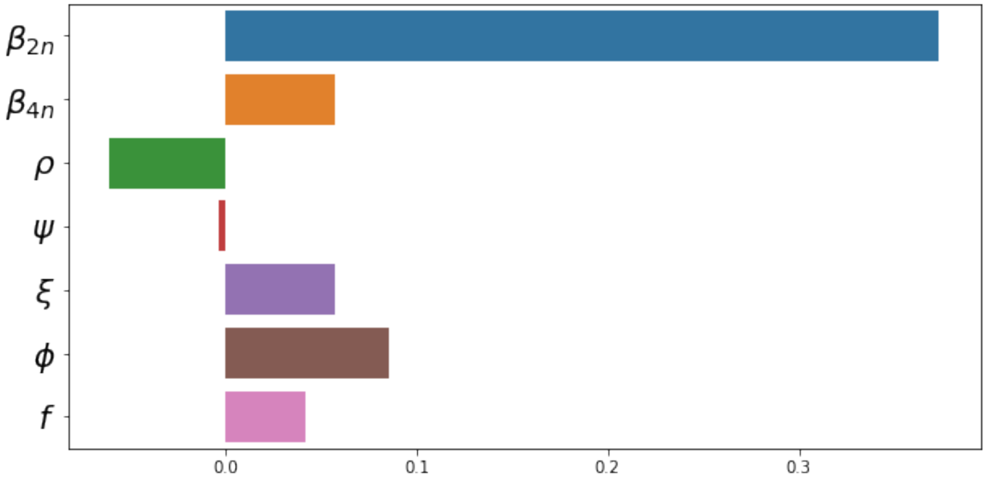

Supplementary Fig. 3 PRCC sensitivity analysis: Sensitivity of iNTS to model parameters, measured by partial rank correlation coefficients.

(higher ρ) decreases them. Finally $\beta_{4,n}$ and ξ show roughly the same effect on the output.

Supplementary Results 1. Different vaccination scenarios and factors driving cases reduction

Supplementary Fig. 6 Cumulative cases prevented 2028-2038 and **Supplementary Fig. 7 Cumulative deaths averted 2028-2038** illustrate the reduction in cases and deaths respectively for each country, under the scenario of a routine and catch-up campaign with 95% vaccine efficacy. There are considerable differences across countries, mostly attributable to their different EPI vaccine coverage levels. This is better captured in **Supplementary Fig. 8 Importance of vaccine coverage**, which reports the reduction in cumulative iNTS cases in the period 2028-2038 in percentage terms, to make it independent of the population size. The colour of each bar corresponds to a coverage level in that country, as captured by the colormap on the righthand side of the graph. There is a clear correspondence between low EPI coverage (below 60%) and lower reduction in cases prevented. To clarify this point even further, we simulate what would happen if the routine coverage level would be at least 90% in any country. The bottom graph in **Supplementary Fig. 8 Importance of vaccine coverage** shows how in almost all countries there is a reduction in cases of at least 50%. Differences among countries in this scenario are then due to differences in population pyramids and comorbidities, and marginally to residual difference in catch-up coverage levels. **Supplementary Table 3 Vaccine efficacy causes** reports the dependence (simple OLS regression) of cumulate percentual reduction in cases (status quo versus routine + catch up vaccination with 95% efficacy) over malaria incidence, percentage of children below 5 years old, percentages of children with HIV and routine coverage: once controlling for coverage, a younger population and higher levels of comorbidities explain lower percentual reduction in cases post vaccination.

We also simulated a vaccination scenario with only a catch-up campaign, and one with only a routine campaign, and both prove to be less effective than a catch-up plus routine campaign, as shown in **??** and **Supplementary Table 4 Yearly cases for all sSA**, which report the cumulative cases prevented for the entire sSA under each scenarios, for vaccine efficacy between 85% and 95%. A catch-up only campaign is clearly the least effective, confirming that in absence of other interventions, like water sanitation, the only way to keep paediatric cases and deaths under control is through sustained routine vaccination.

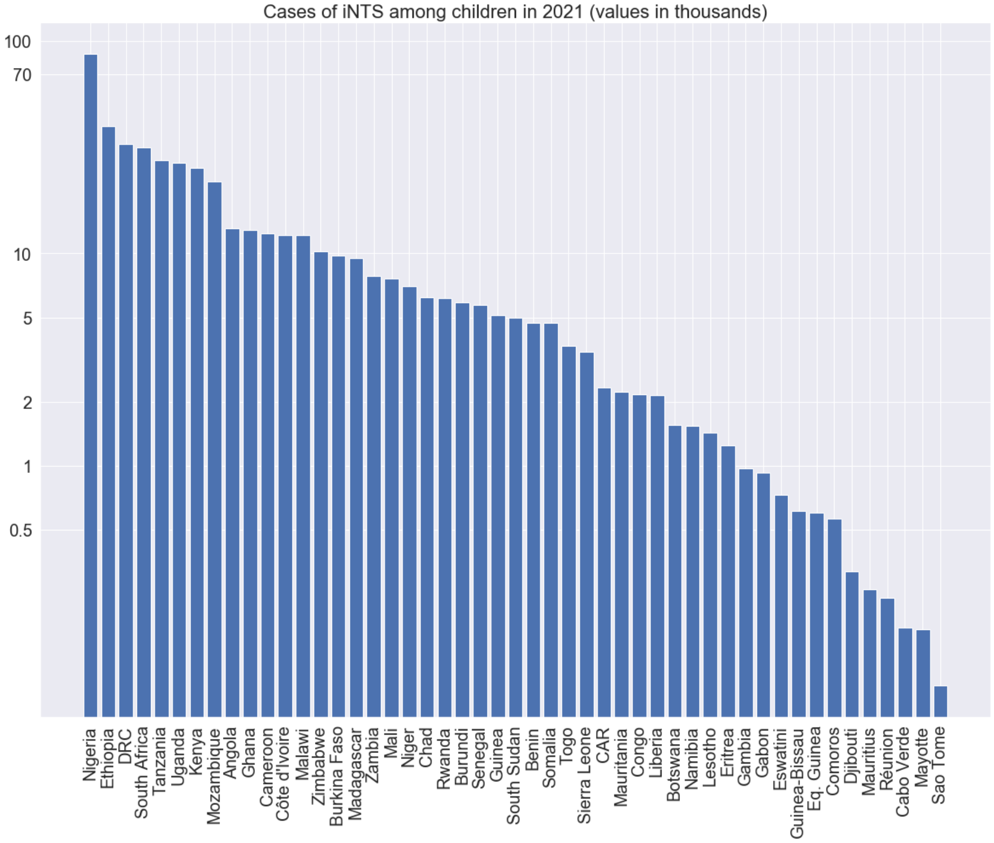

Supplementary Fig. 4 Cases in 2021: Yearly number of cases in 2021 for each SSA Country, where the y-axis is in logarithmic scale.

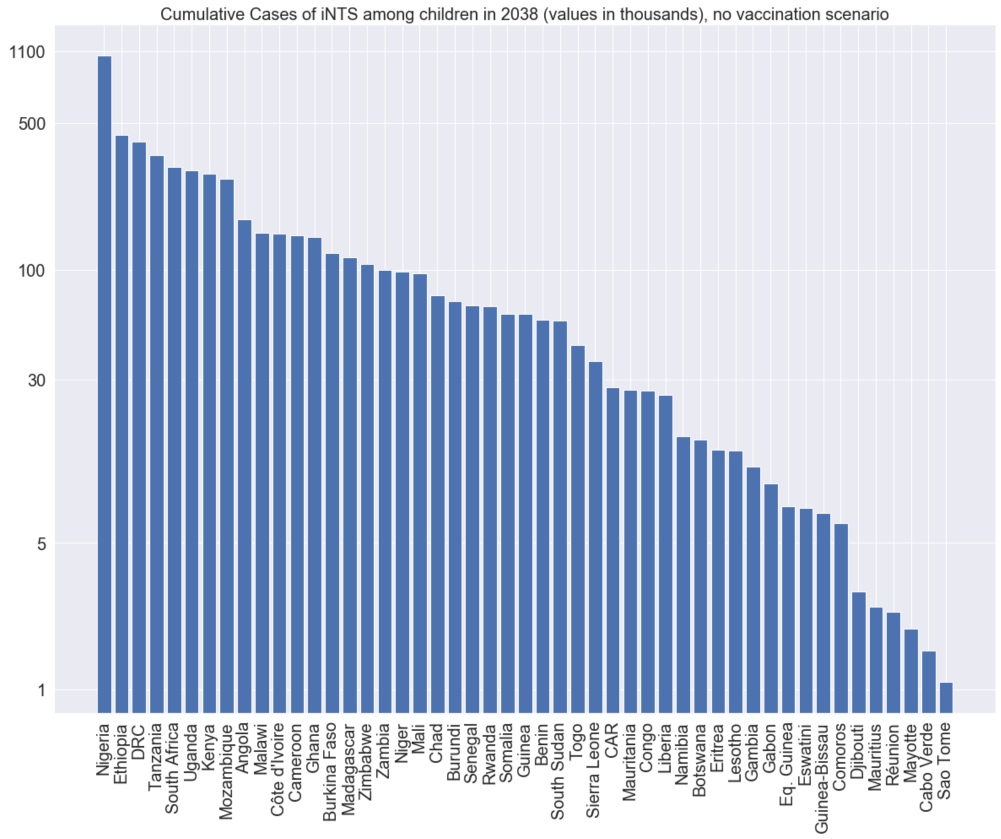

Supplementary Fig. 5 Cumulative cases up to 2038: Cumulative iNTS cases among children below 5 years old up to 2038 for each country, where the y-axis is in logarithmic scale.

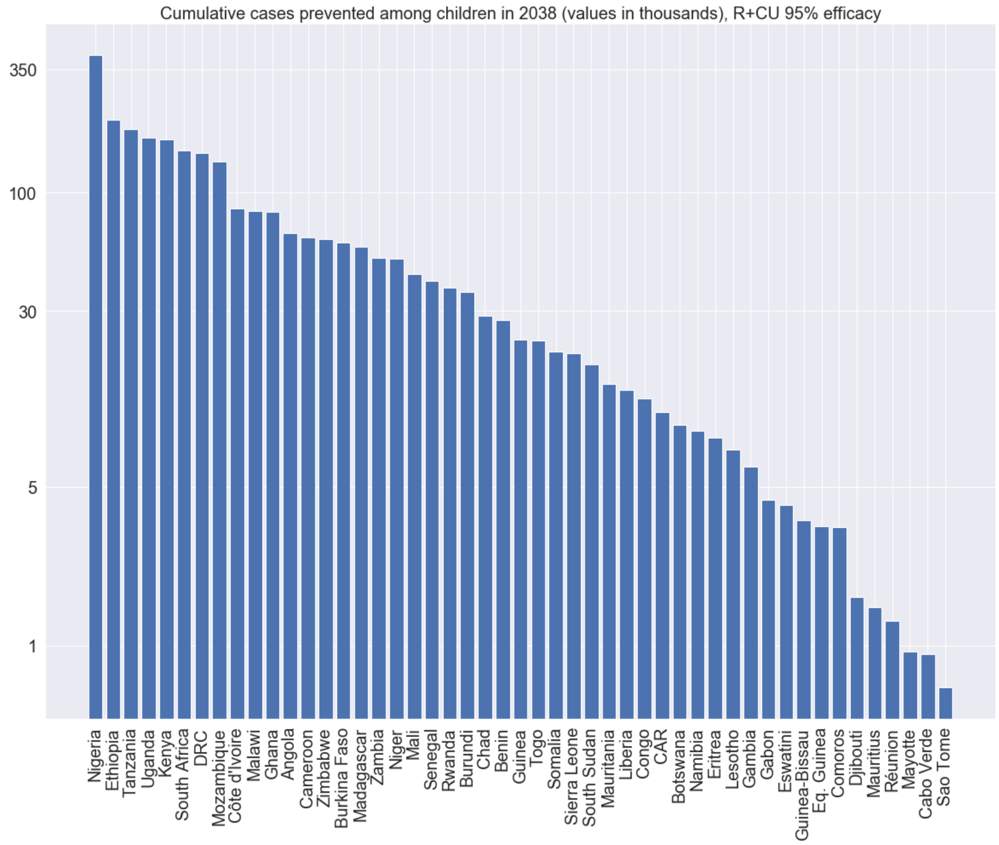

Supplementary Fig. 6 Cumulative cases prevented 2028-2038: Cumulative cases prevented for each country during the period 2028-2038. Routine + catch-up campaign with 95% vaccine efficacy. y-axis is on a logarithmic scale.

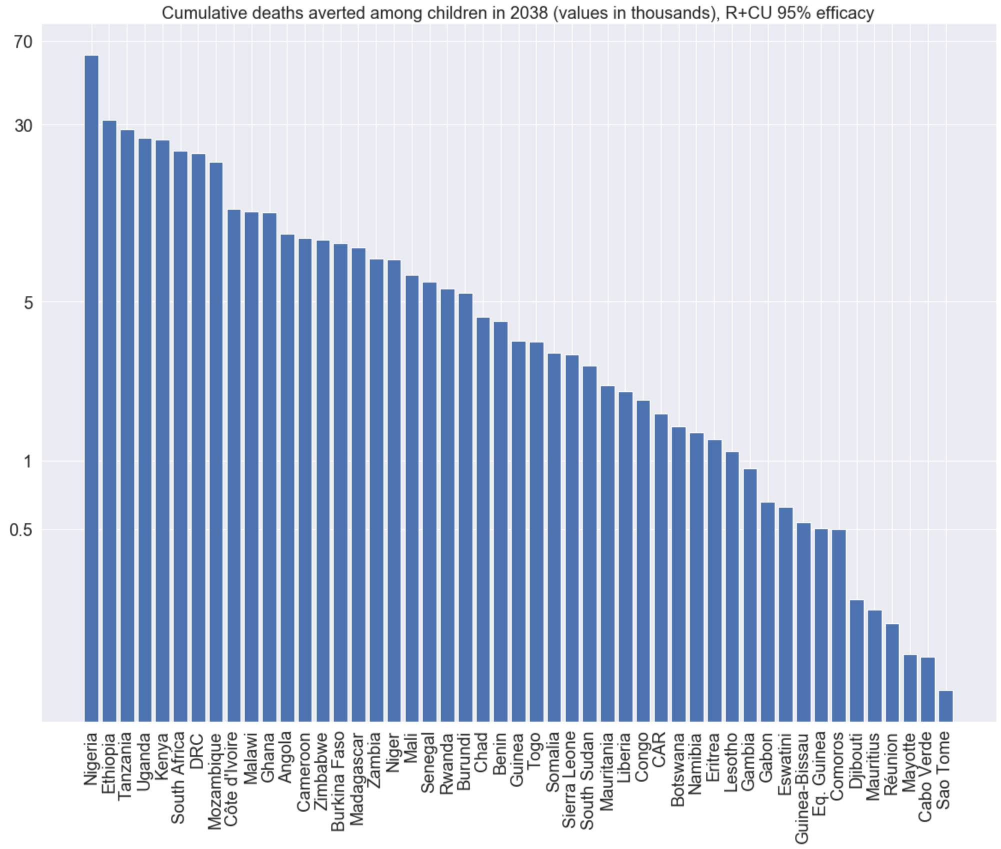

Supplementary Fig. 7 Cumulative deaths averted 2028-2038: Cumulative deaths averted for each country during the period 2028-2038. Routine + catch-up campaign with 95% vaccine efficacy and 20% mortality. y-axis is on a logarithmic scale.

Supplementary Table 3 Vaccine efficacy causes: Vaccine efficacy causes

	Percentage reduction in cases	
Intercept	0.0048	(0.018)
Malaria	−0.1935***	(0.017)
Children below 5 y.o.	−0.1348*	(0.067)
Children with HIV	−0.3854**	(0.155)
Coverage DTP1	0.6274***	(0.014)
Observations	49	
R-squared	0.983	
Joint significance (p-value F-statistics)	0.00	

standard error in parentheses

$^{*}\ p < 0.10$, $^{**}\ p < 0.05$, $^{***}\ p < 0.01$

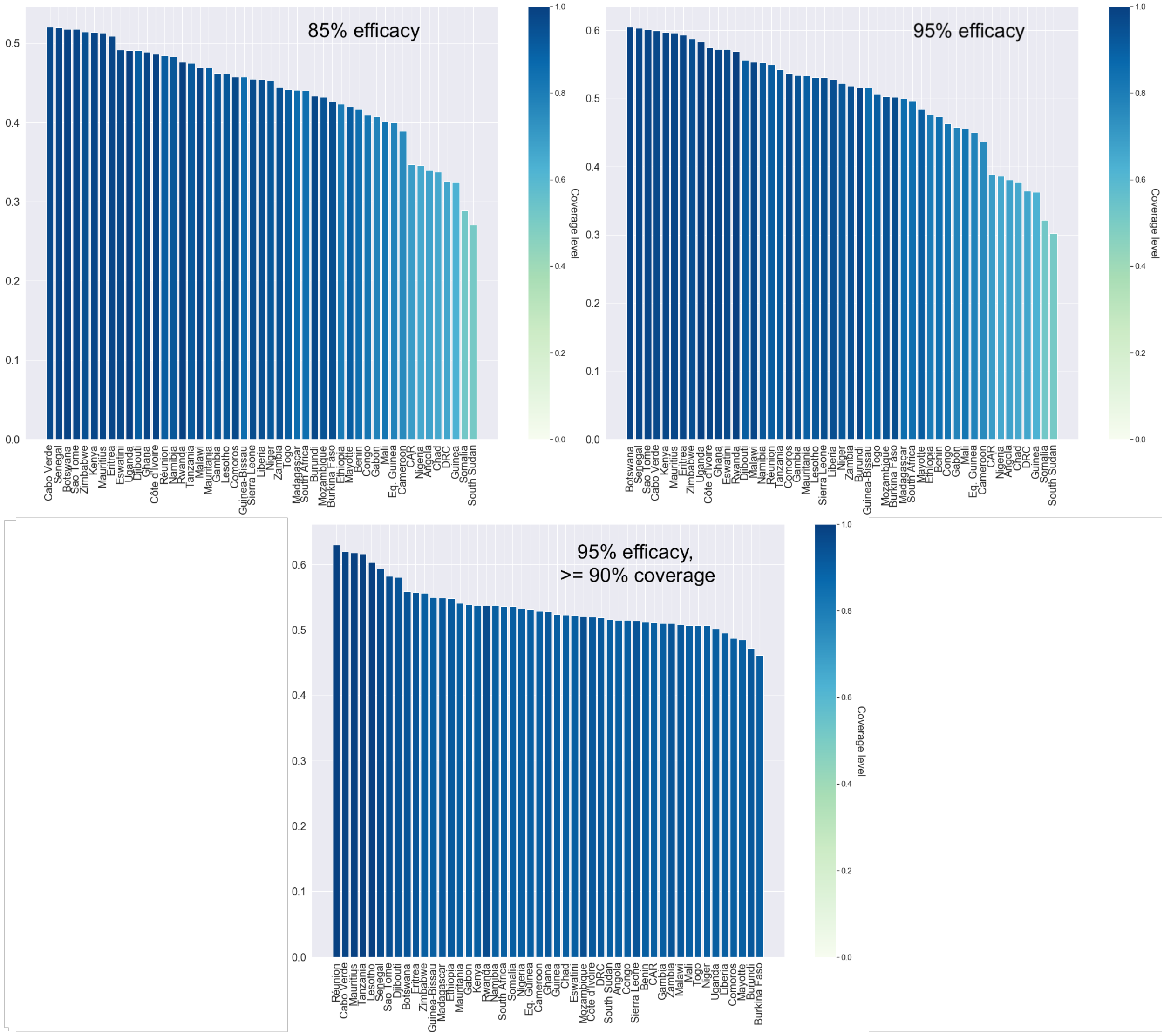

Supplementary Fig. 8 Importance of vaccine coverage: Percentage reduction of iNTS cases compared to status quo following a routine + catch-up campaign between 2028 and 2038 at actual coverage levels (top) with 85% (left), 95% (right) vaccine efficacy and at coverage levels at or above 90% (bottom) with 95% vaccine efficacy. Bar colors identify coverage levels.

Supplementary Table 4 Yearly cases for all sSA: Cases (thousands) per year among children below 5 y.o., for the aggregate sSA, all scenarios.

Year	No vac	CU + R	R only	CU only
2021	478.152	-	-	-
2022	484.596	-	-	-
2023	491.225	-	-	-
2024	498.071	-	-	-
2025	505.150	-	-	-
2026	512.928	-	-	-
2027	520.900	-	-	-
2028	528.813	[318.455, 293.699]	[528.813, 528.813]	[318.455, 293.699]
2029	537.225	[354.151, 322.101]	[388.426, 356.546]	[485.705, 479.643]
2030	545.568	[315.018, 280.579]	[317.84, 282.759]	[532.167, 530.589]
2031	553.562	[316.668, 282.525]	[316.918, 282.687]	[549.99, 549.568]
2032	561.419	[320.999, 286.475]	[321.008, 286.476]	[560.462, 560.353]
2033	569.080	[325.438, 290.464]	[325.424, 290.456]	[568.833, 568.802]
2034	576.480	[329.737, 294.325]	[329.726, 294.317]	[576.419, 576.413]
2035	583.579	[333.863, 298.034]	[333.855, 298.026]	[583.572, 583.569]
2036	589.632	[337.409, 301.218]	[337.4, 301.207]	[589.638, 589.637]
2037	595.429	[340.79, 304.263]	[340.783, 304.255]	[595.436, 595.438]
2038	601.016	[344.061, 307.202]	[344.057, 307.201]	[601.024, 601.025]
Total 2021-2038	9732.825			
Total 2028-2038	6241.803	[3636.589, 3260.885]	[3884.25, 3532.743]	[5928.736, 5961.701]

Supplementary Result 2. Per-country vaccine effectiveness

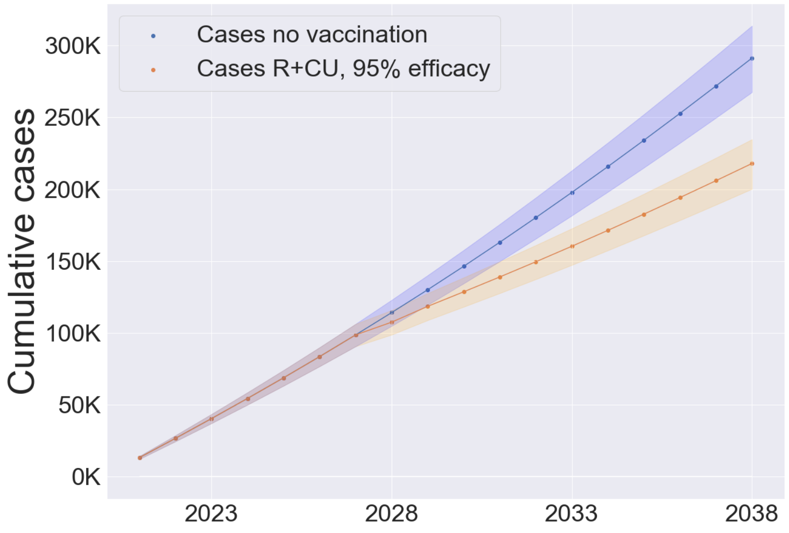

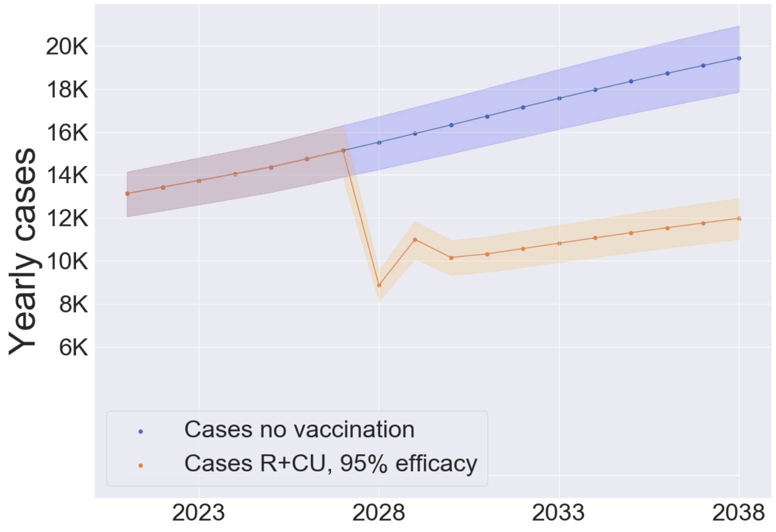

Supplementary Fig. 9 Angola cumulative and yearly iNTS cases: Angola cumulative (top) and yearly (bottom) iNTS cases under the status quo and routine + catch-up vaccination (95% efficacy) scenarios. Shaded areas show the 25th and 75th percentiles, line shows the median over 1000 experiments, samples drawn from uniform distributions over (0.00020,0.00024) for $\beta_{2,n}$ and (0.0080,0.0084) for $\beta_{4,n}$.

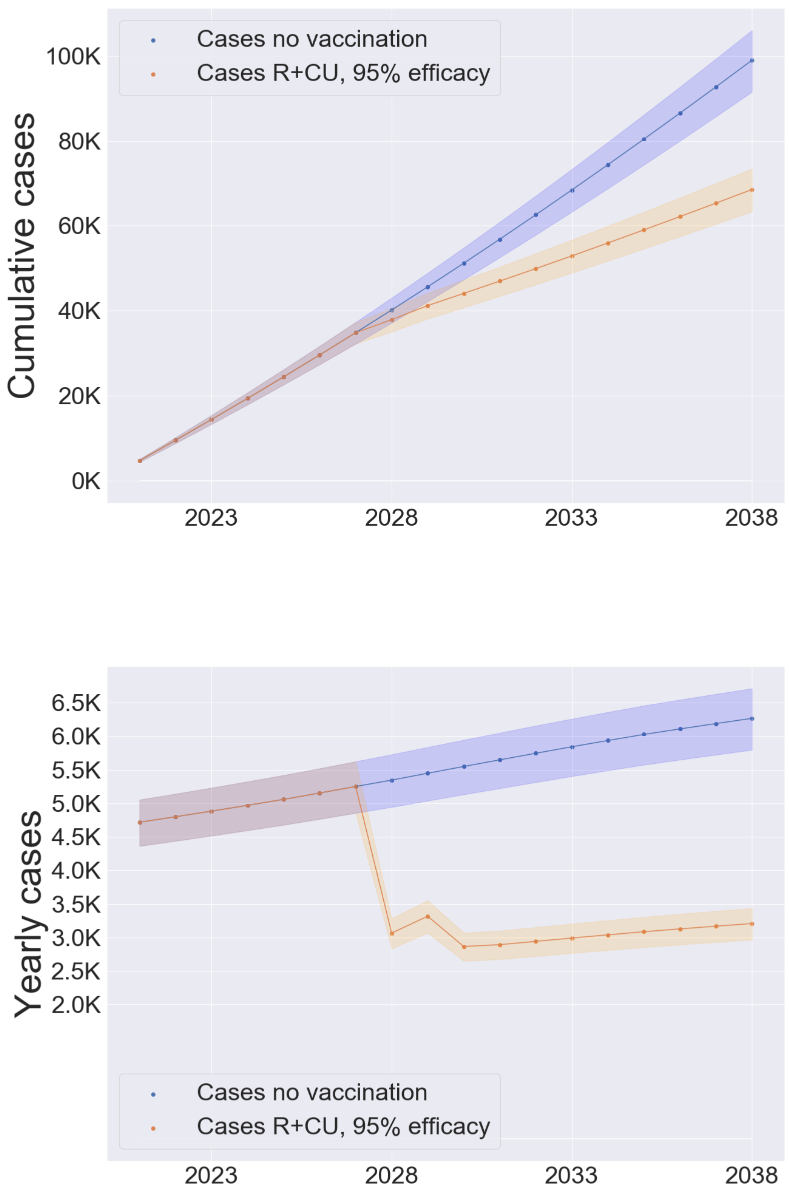

Supplementary Fig. 10 Benin cumulative and yearly iNTS cases: Benin cumulative (top) and yearly (bottom) iNTS cases under the status quo and routine + catch-up vaccination (95% efficacy) scenarios. Shaded areas show the 25th and 75th percentiles, line shows the median over 1000 experiments, samples drawn from uniform distributions over (0.00020,0.00024) for $\beta_{2,n}$ and (0.0080,0.0084) for $\beta_{4,n}$.

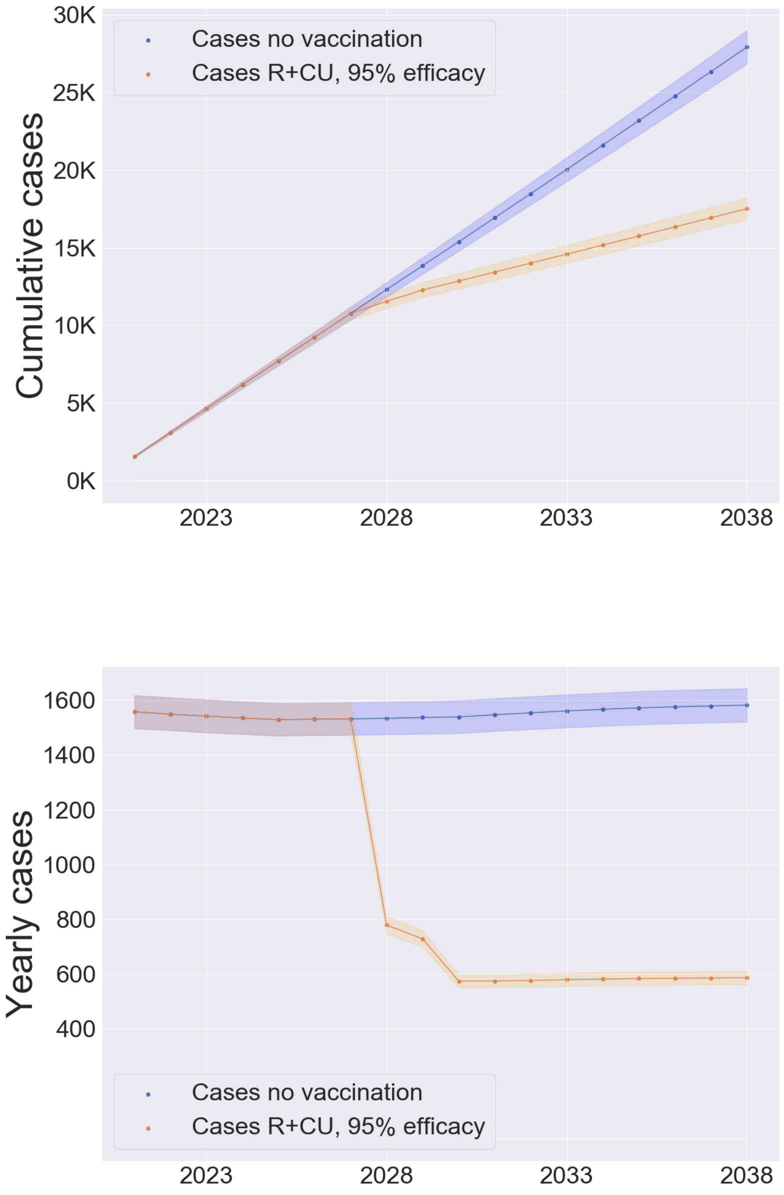

Supplementary Fig. 11 Botswana cumulative and yearly iNTS cases: Botswana cumulative (top) and yearly (bottom) iNTS cases under the status quo and routine + catch-up vaccination (95% efficacy) scenarios. Shaded areas show the 25th and 75th percentiles, line shows the median over 1000 experiments, samples drawn from uniform distributions over (0.00020,0.00024) for $\beta_{2,n}$ and (0.0080,0.0084) for $\beta_{4,n}$.

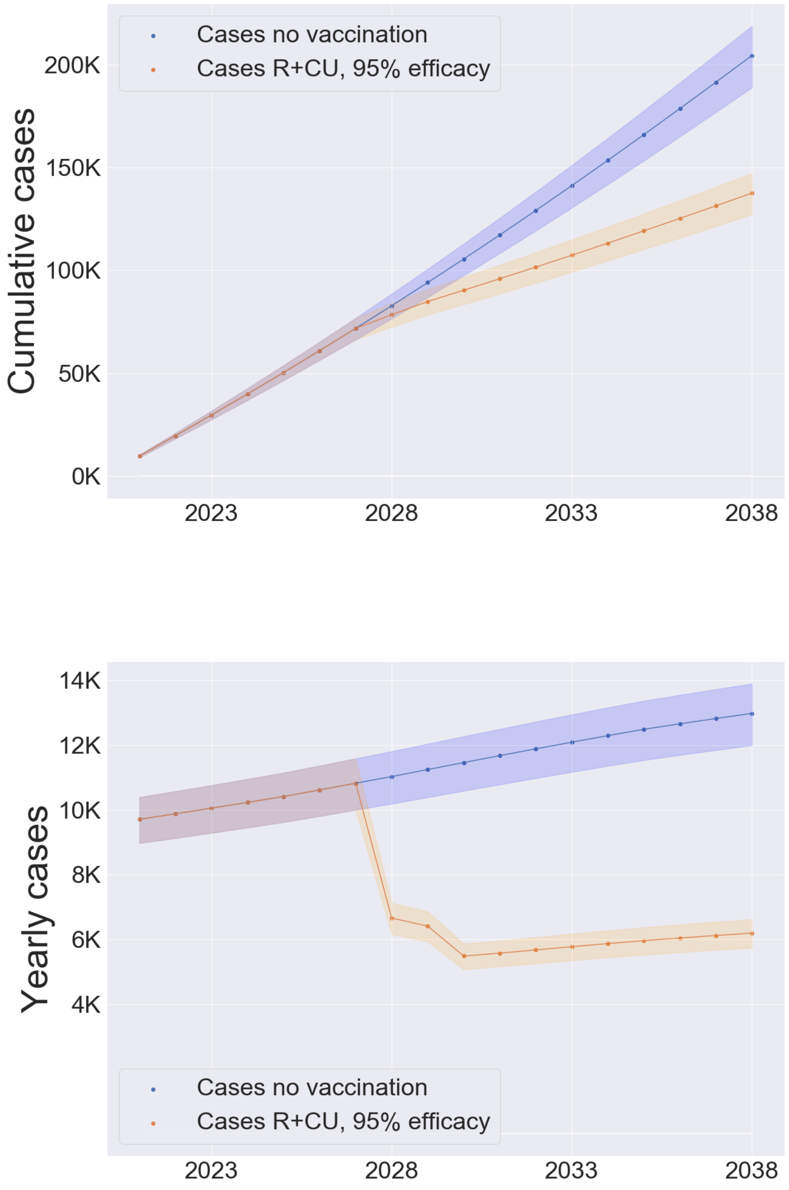

Supplementary Fig. 12 Burkina Faso cumulative and yearly iNTS cases: Burkina Faso cumulative (top) and yearly (bottom) iNTS cases under the status quo and routine + catch-up vaccination (95% efficacy) scenarios. Shaded areas show the 25th and 75th percentiles, line shows the median over 1000 experiments, samples drawn from uniform distributions over (0.00020,0.00024) for $\beta_{2,n}$ and (0.0080,0.0084) for $\beta_{4,n}$.

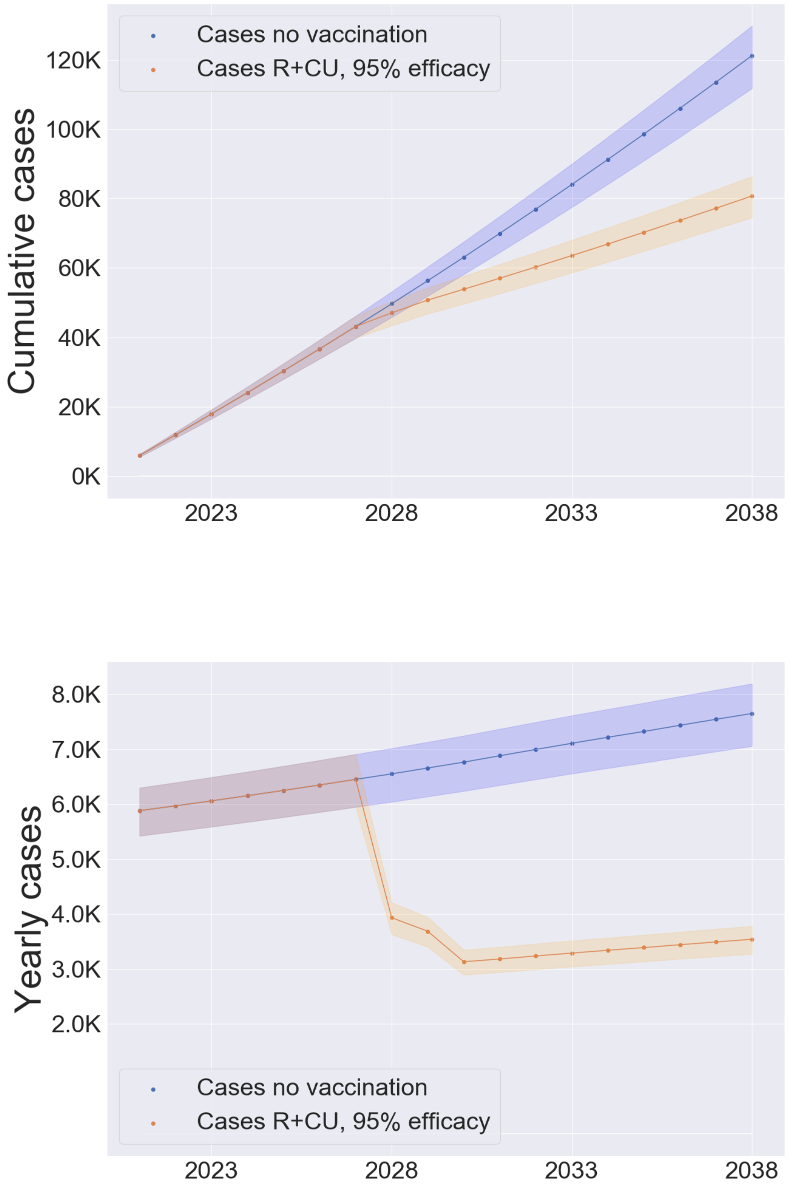

Supplementary Fig. 13 Burundi cumulative and yearly iNTS cases: Burundi cumulative (top) and yearly (bottom) iNTS cases under the status quo and routine + catch-up vaccination (95% efficacy) scenarios. Shaded areas show the 25th and 75th percentiles, line shows the median over 1000 experiments, samples drawn from uniform distributions over (0.00020,0.00024) for $\beta_{2,n}$ and (0.0080,0.0084) for $\beta_{4,n}$.

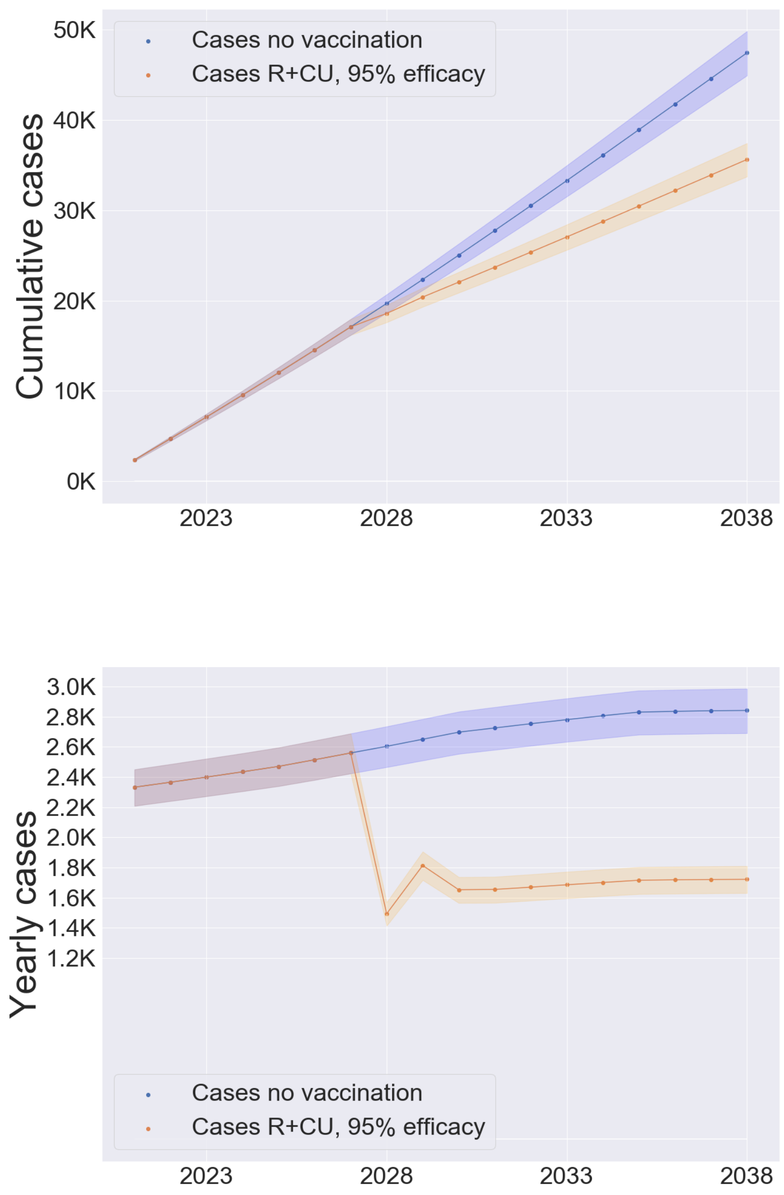

Supplementary Fig. 14 Central African Republic cumulative and yearly iNTS cases: Central African Republic cumulative (top) and yearly (bottom) iNTS cases under the status quo and routine + catch-up vaccination (95% efficacy) scenarios. Shaded areas show the 25th and 75th percentiles, line shows the median over 1000 experiments, samples drawn from uniform distributions over (0.00020,0.00024) for $\beta_{2,n}$ and (0.0080,0.0084) for $\beta_{4,n}$.

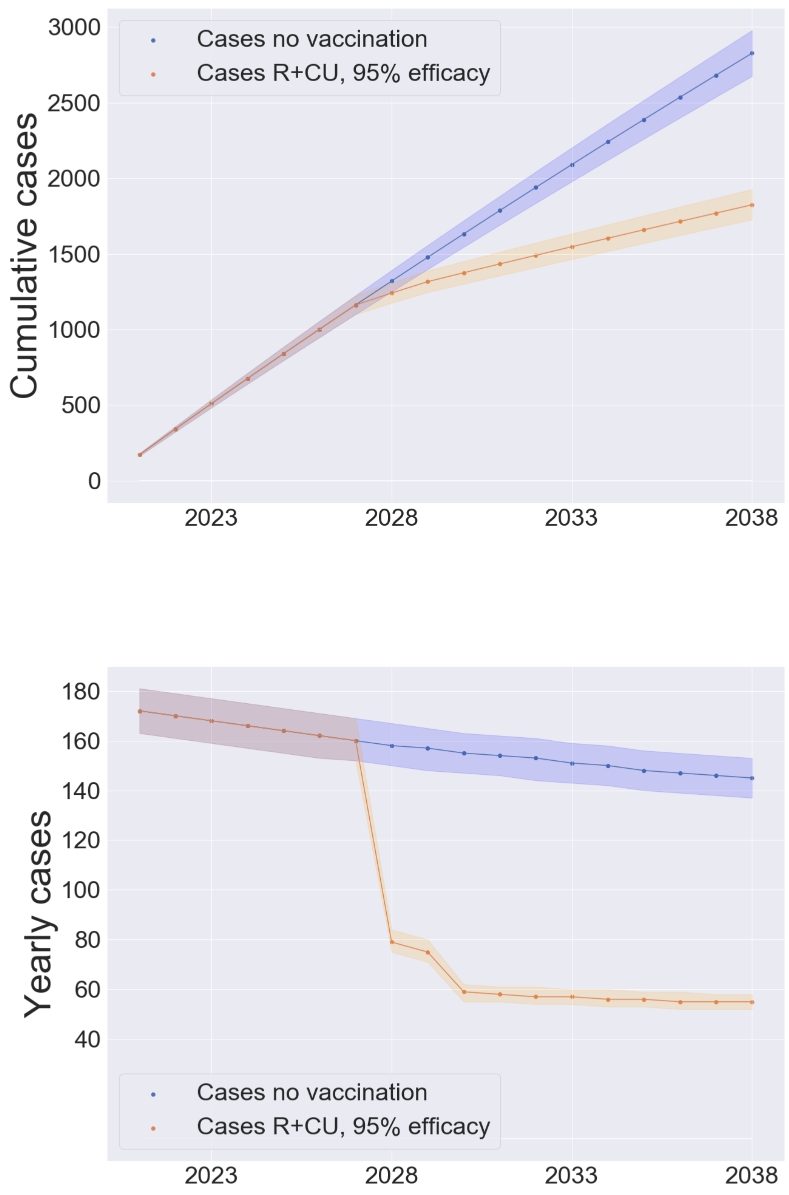

Supplementary Fig. 15 Cabo Verde cumulative and yearly iNTS cases: Cabo Verde cumulative (top) and yearly (bottom) iNTS cases under the status quo and routine + catch-up vaccination (95% efficacy) scenarios. Shaded areas show the 25th and 75th percentiles, line shows the median over 1000 experiments, samples drawn from uniform distributions over (0.00020,0.00024) for $\beta_{2,n}$ and (0.0080,0.0084) for $\beta_{4,n}$.

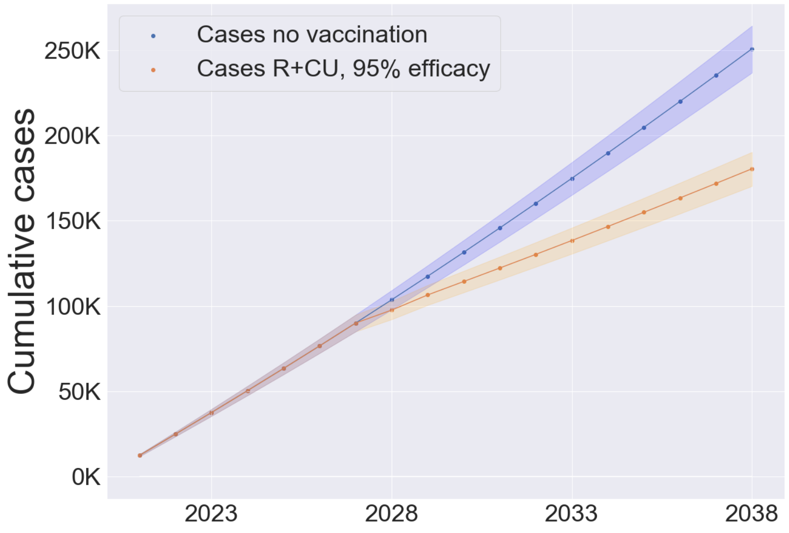

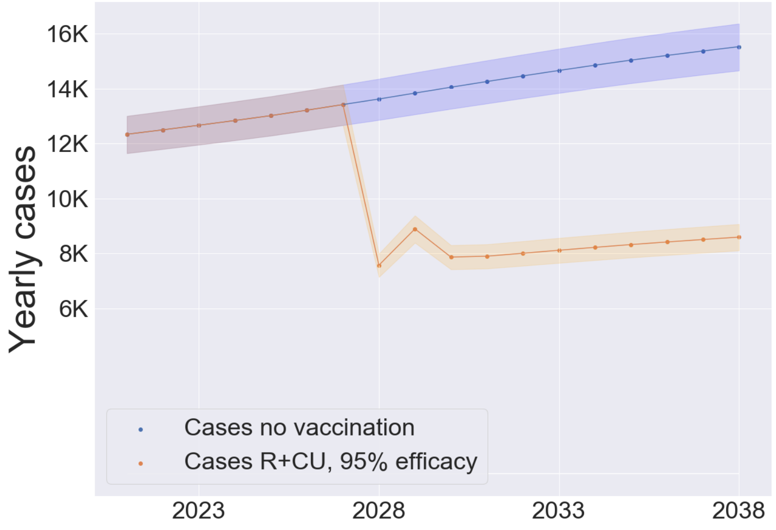

Supplementary Fig. 16 Cameroon cumulative and yearly iNTS cases: Cameroon cumulative (top) and yearly (bottom) iNTS cases under the status quo and routine + catch-up vaccination (95% efficacy) scenarios. Shaded areas show the 25th and 75th percentiles, line shows the median over 1000 experiments, samples drawn from uniform distributions over (0.00020,0.00024) for $\beta_{2,n}$ and (0.0080,0.0084) for $\beta_{4,n}$.

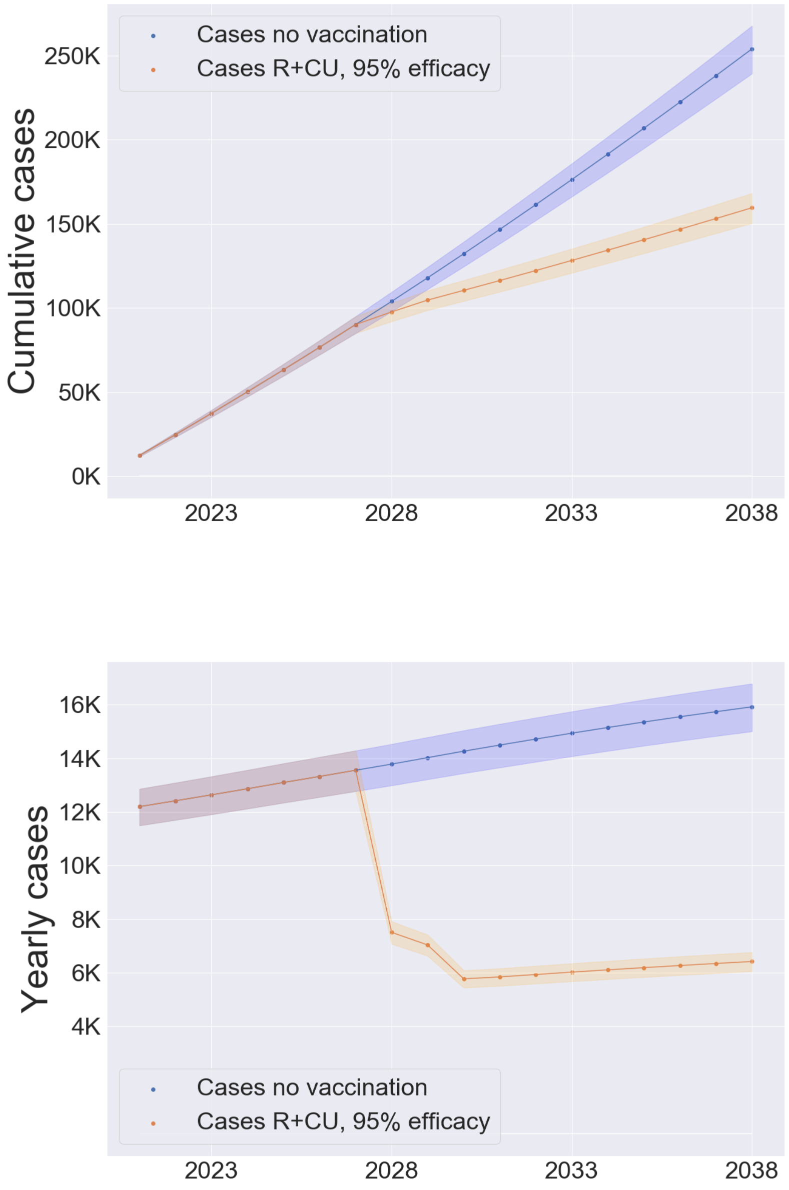

Supplementary Fig. 17 Côte d'Ivoire cumulative and yearly iNTS cases: Côte d'Ivoire cumulative (top) and yearly (bottom) iNTS cases under the status quo and routine + catch-up vaccination (95% efficacy) scenarios. Shaded areas show the 25th and 75th percentiles, line shows the median over 1000 experiments, samples drawn from uniform distributions over (0.00020,0.00024) for $\beta_{2,n}$ and (0.0080,0.0084) for $\beta_{4,n}$.

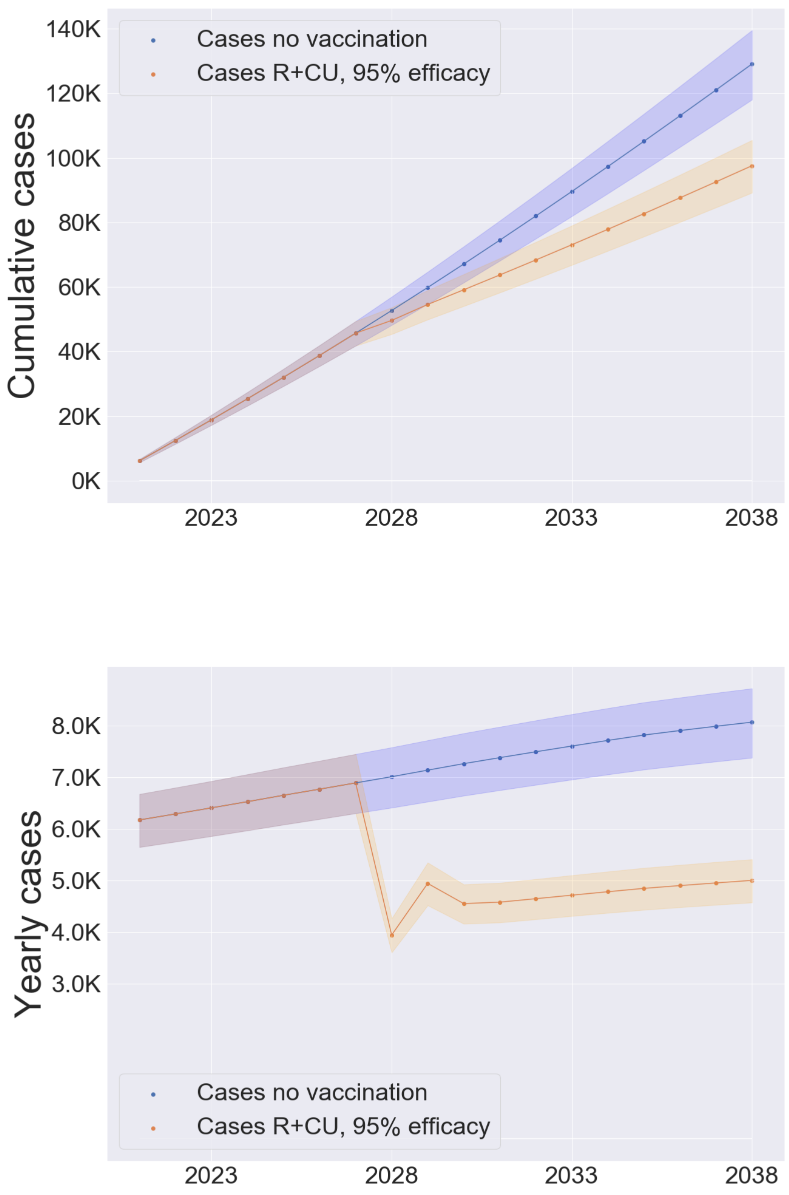

Supplementary Fig. 18 Chad cumulative and yearly iNTS cases: Chad cumulative (top) and yearly (bottom) iNTS cases under the status quo and routine + catch-up vaccination (95% efficacy) scenarios. Shaded areas show the 25th and 75th percentiles, line shows the median over 1000 experiments, samples drawn from uniform distributions over (0.00020,0.00024) for $\beta_{2,n}$ and (0.0080,0.0084) for $\beta_{4,n}$.

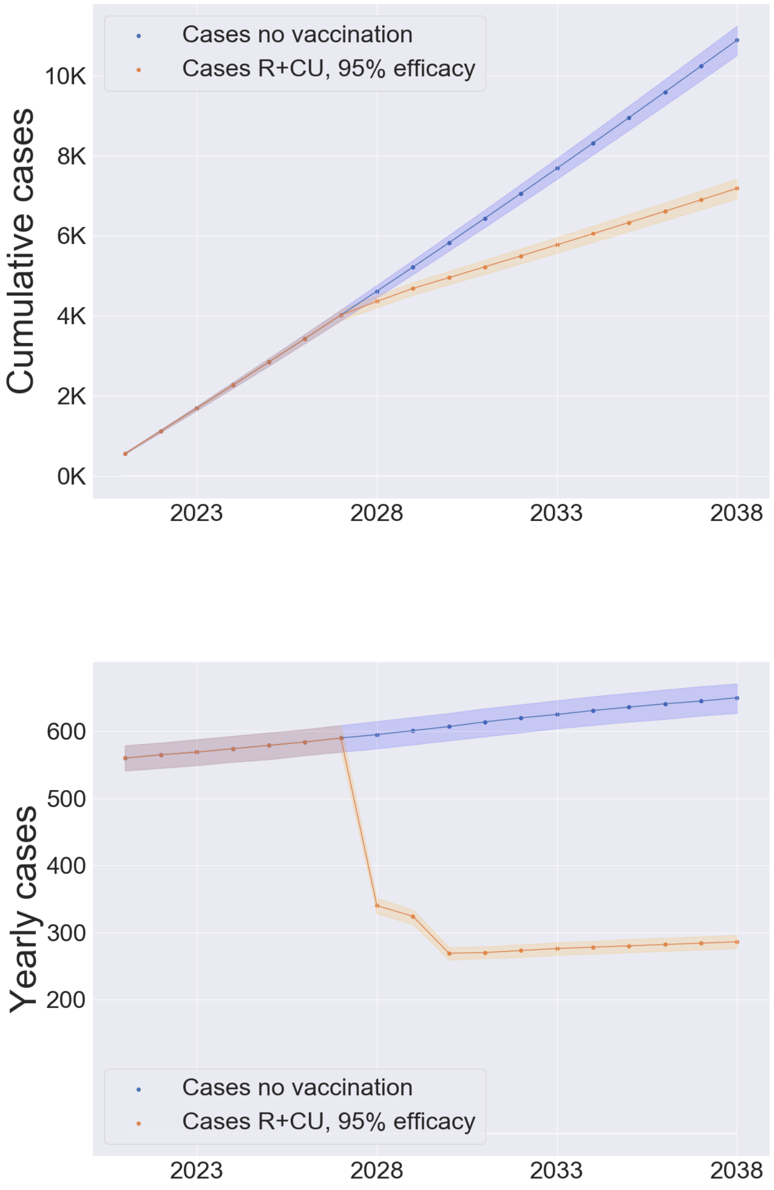

Supplementary Fig. 19 Comoros cumulative and yearly iNTS cases: Comoros cumulative (top) and yearly (bottom) iNTS cases under the status quo and routine + catch-up vaccination (95% efficacy) scenarios. Shaded areas show the 25th and 75th percentiles, line shows the median over 1000 experiments, samples drawn from uniform distributions over (0.00020,0.00024) for $\beta_{2,n}$ and (0.0080,0.0084) for $\beta_{4,n}$.

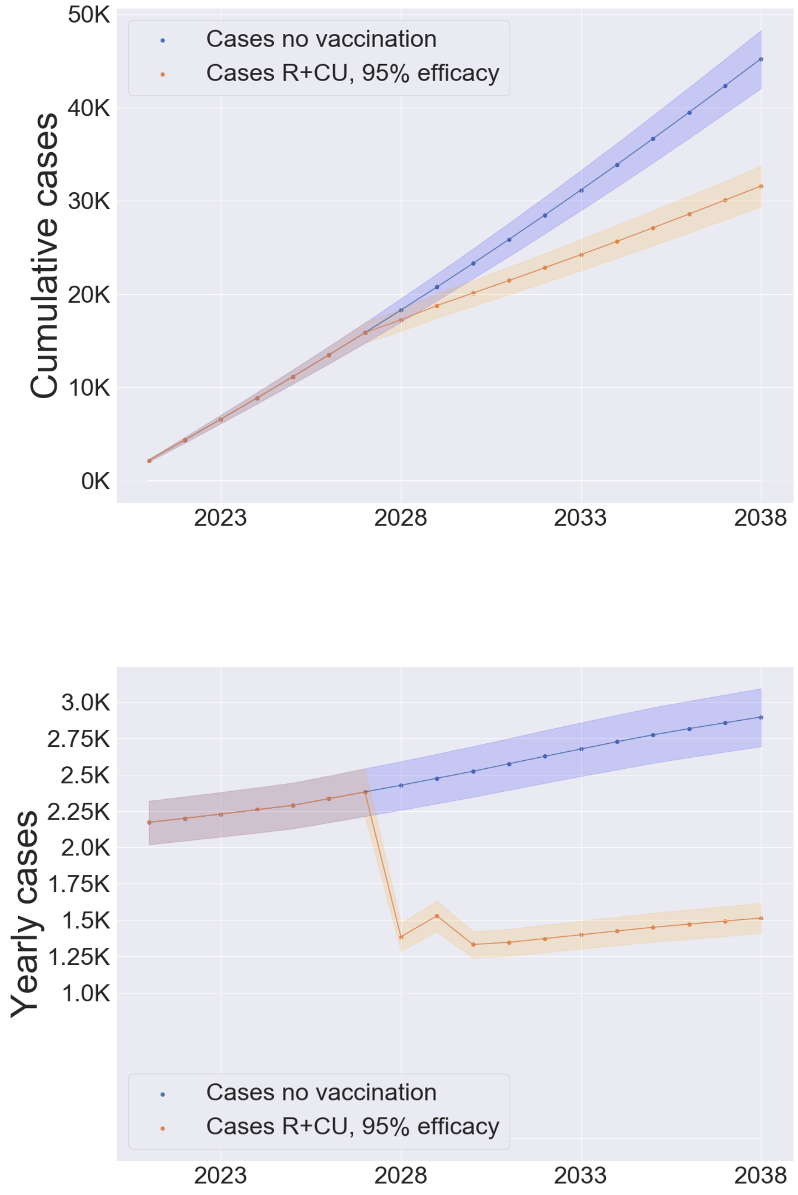

Supplementary Fig. 20 Congo cumulative and yearly iNTS cases: Congo cumulative (top) and yearly (bottom) iNTS cases under the status quo and routine + catch-up vaccination (95% efficacy) scenarios. Shaded areas show the 25th and 75th percentiles, line shows the median over 1000 experiments, samples drawn from uniform distributions over (0.00020,0.00024) for $\beta_{2,n}$ and (0.0080,0.0084) for $\beta_{4,n}$.

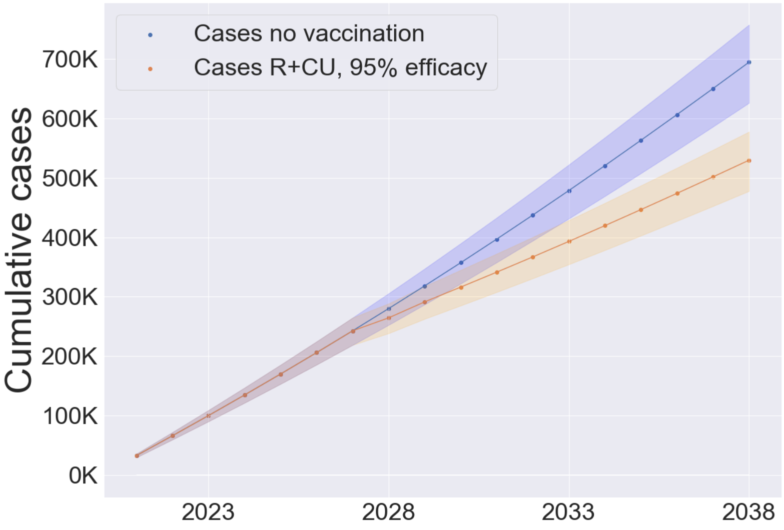

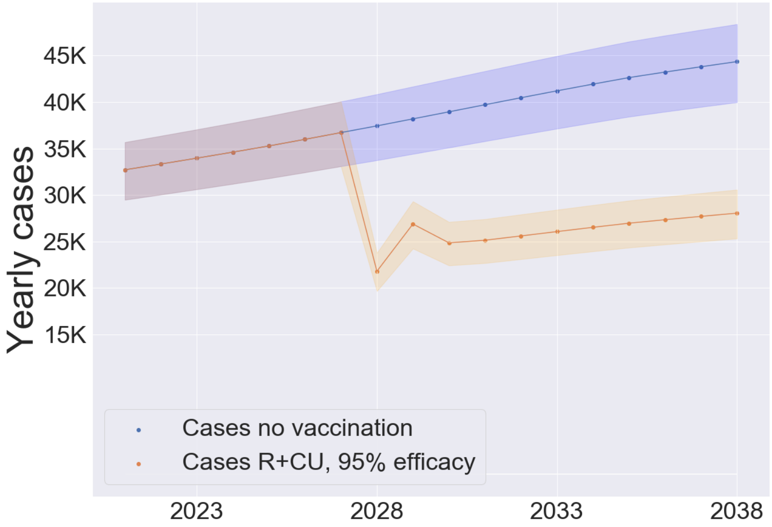

Supplementary Fig. 21 Democratic Republic of the Congo cumulative and yearly iNTS cases: Democratic Republic of the Congo cumulative (top) and yearly (bottom) iNTS cases under the status quo and routine + catch-up vaccination (95% efficacy) scenarios. Shaded areas show the 25th and 75th percentiles, line shows the median over 1000 experiments, samples drawn from uniform distributions over (0.00020,0.00024) for $\beta_{2,n}$ and (0.0080,0.0084) for $\beta_{4,n}$.

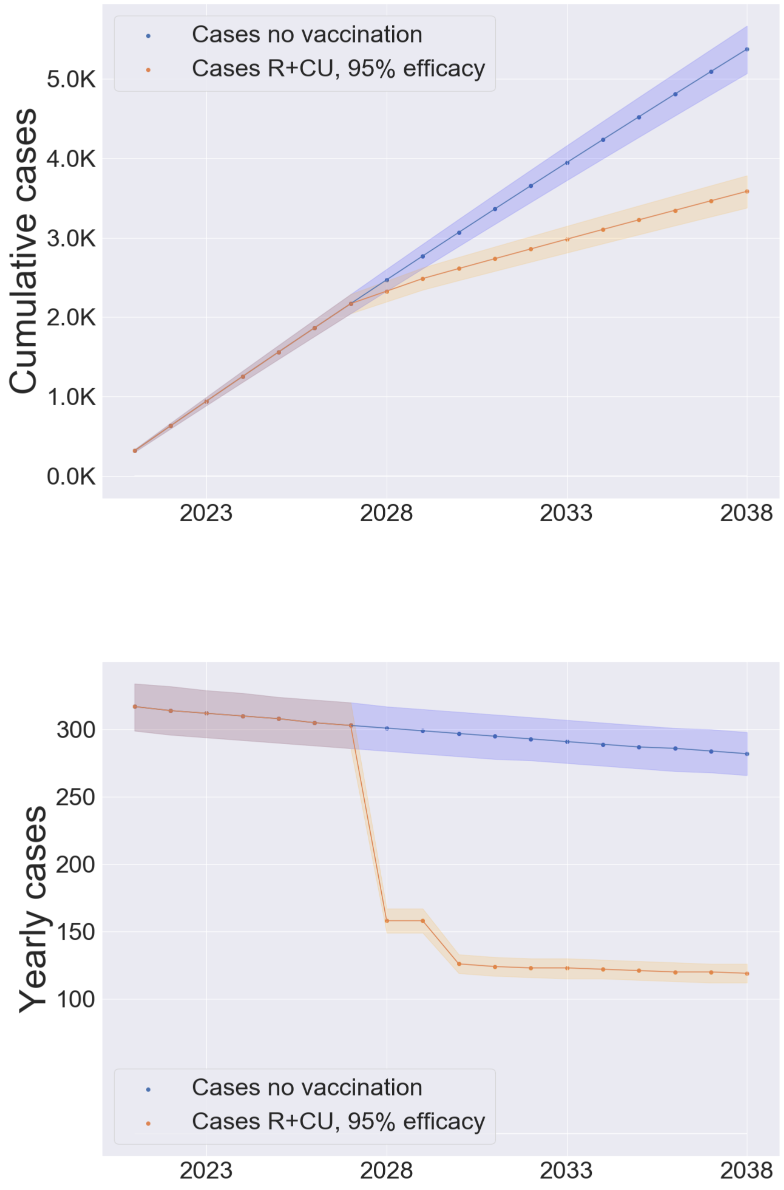

Supplementary Fig. 22 Djibouti cumulative and yearly iNTS cases: Djibouti cumulative (top) and yearly (bottom) iNTS cases under the status quo and routine + catch-up vaccination (95% efficacy) scenarios. Shaded areas show the 25th and 75th percentiles, line shows the median over 1000 experiments, samples drawn from uniform distributions over (0.00020,0.00024) for $\beta_{2,n}$ and (0.0080,0.0084) for $\beta_{4,n}$.

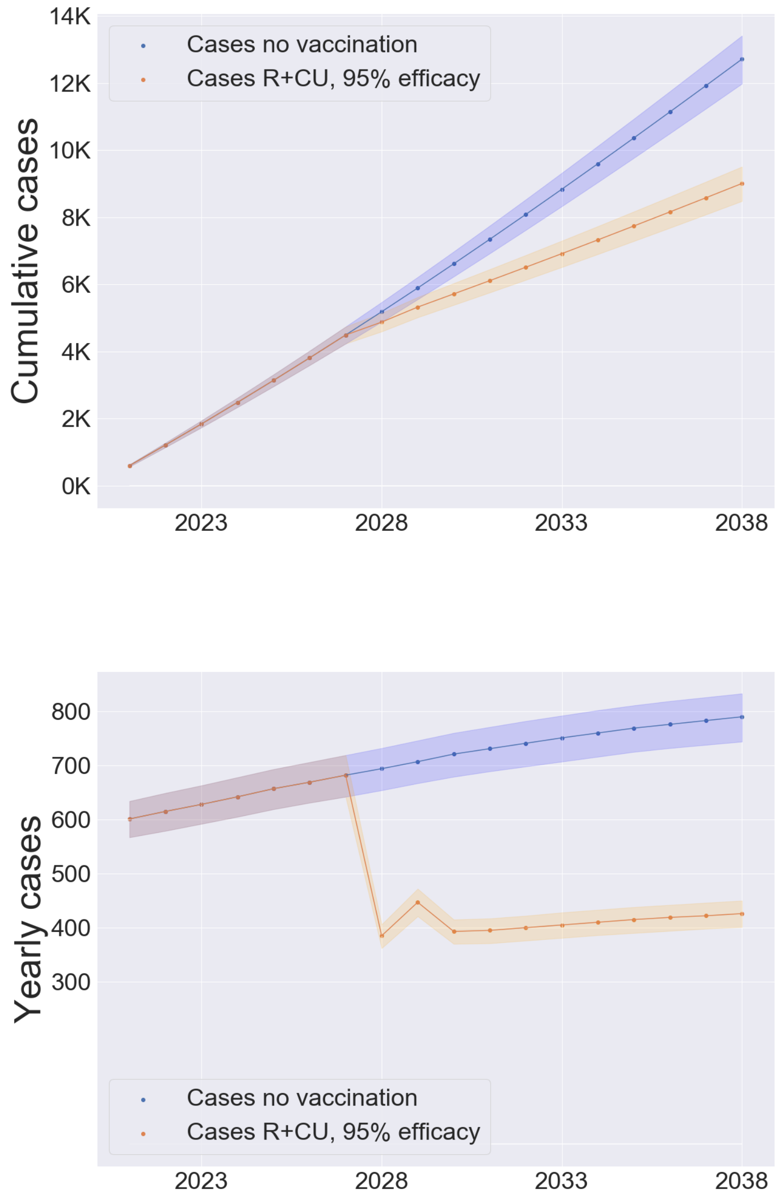

Supplementary Fig. 23 Equatorial Guinea cumulative and yearly iNTS cases: Equatorial Guinea cumulative (top) and yearly (bottom) iNTS cases under the status quo and routine + catch-up vaccination (95% efficacy) scenarios. Shaded areas show the 25th and 75th percentiles, line shows the median over 1000 experiments, samples drawn from uniform distributions over (0.00020,0.00024) for $\beta_{2,n}$ and (0.0080,0.0084) for $\beta_{4,n}$.

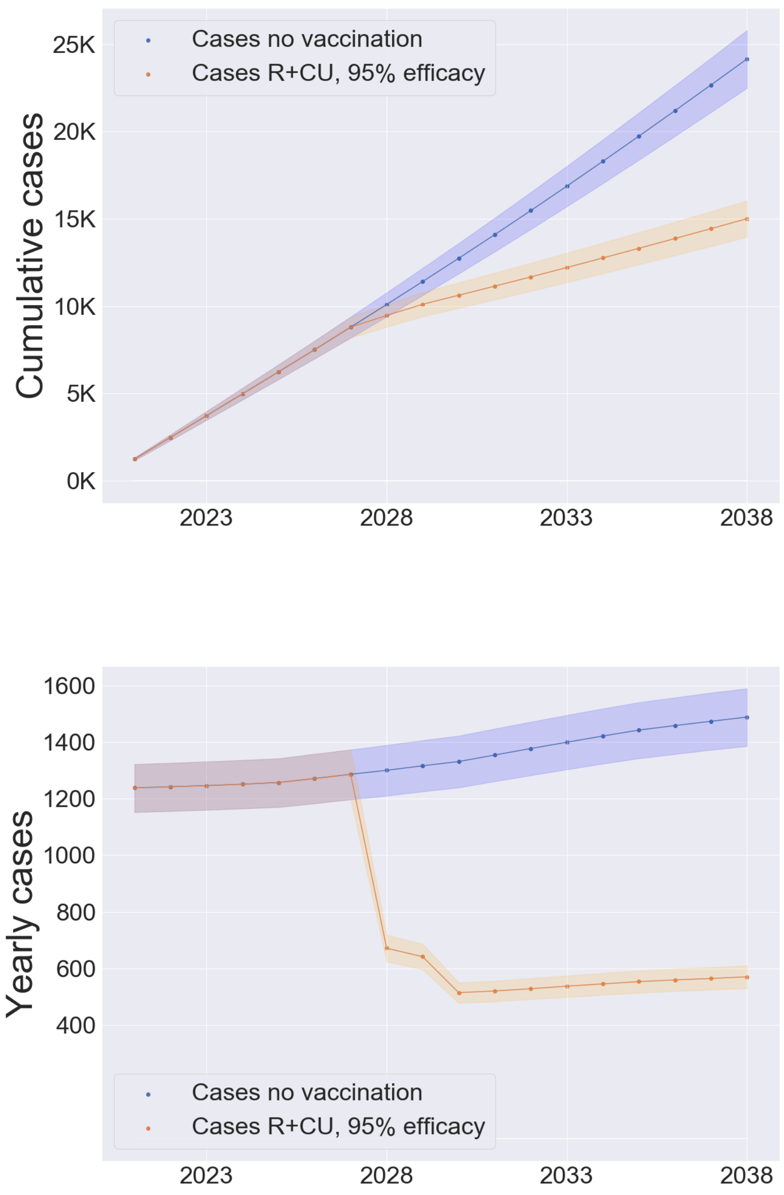

Supplementary Fig. 24 Eritrea cumulative and yearly iNTS cases: Eritrea cumulative (top) and yearly (bottom) iNTS cases under the status quo and routine + catch-up vaccination (95% efficacy) scenarios. Shaded areas show the 25th and 75th percentiles, line shows the median over 1000 experiments, samples drawn from uniform distributions over (0.00020,0.00024) for $\beta_{2,n}$ and (0.0080,0.0084) for $\beta_{4,n}$.

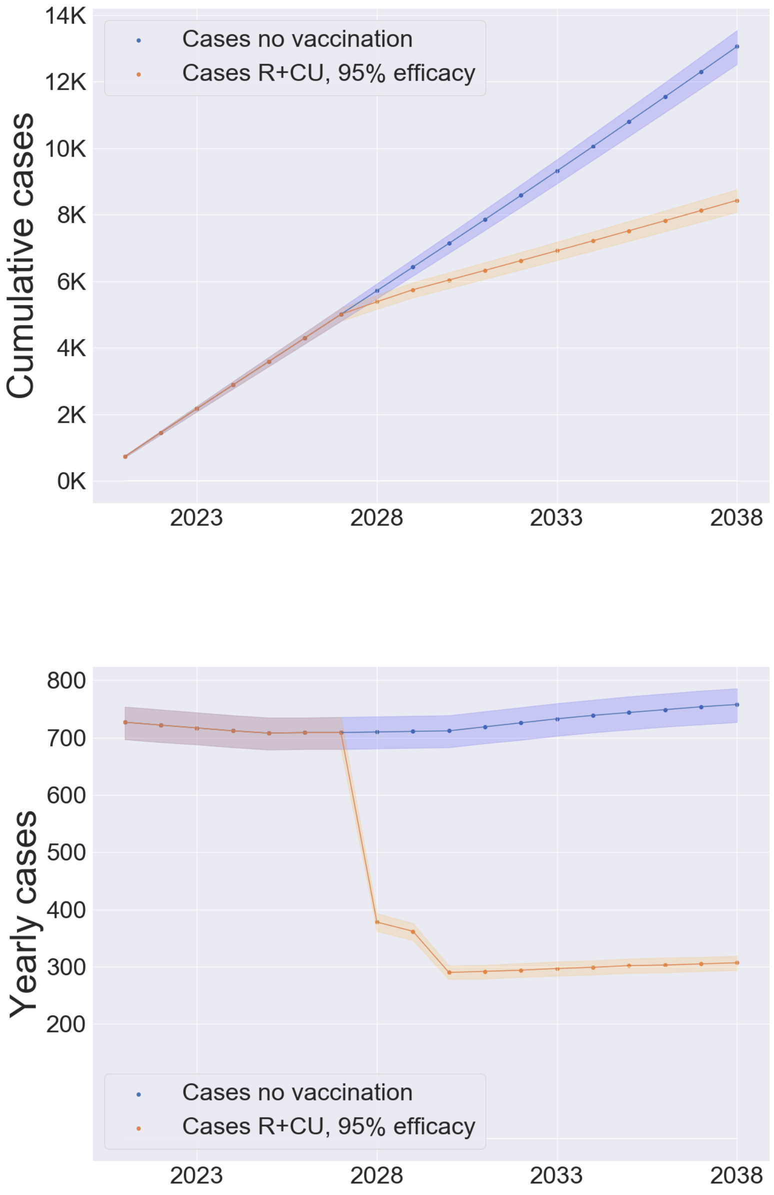

Supplementary Fig. 25 Eswatini cumulative and yearly iNTS cases: Eswatini cumulative (top) and yearly (bottom) iNTS cases under the status quo and routine + catch-up vaccination (95% efficacy) scenarios. Shaded areas show the 25th and 75th percentiles, line shows the median over 1000 experiments, samples drawn from uniform distributions over (0.00020,0.00024) for $\beta_{2,n}$ and (0.0080,0.0084) for $\beta_{4,n}$.

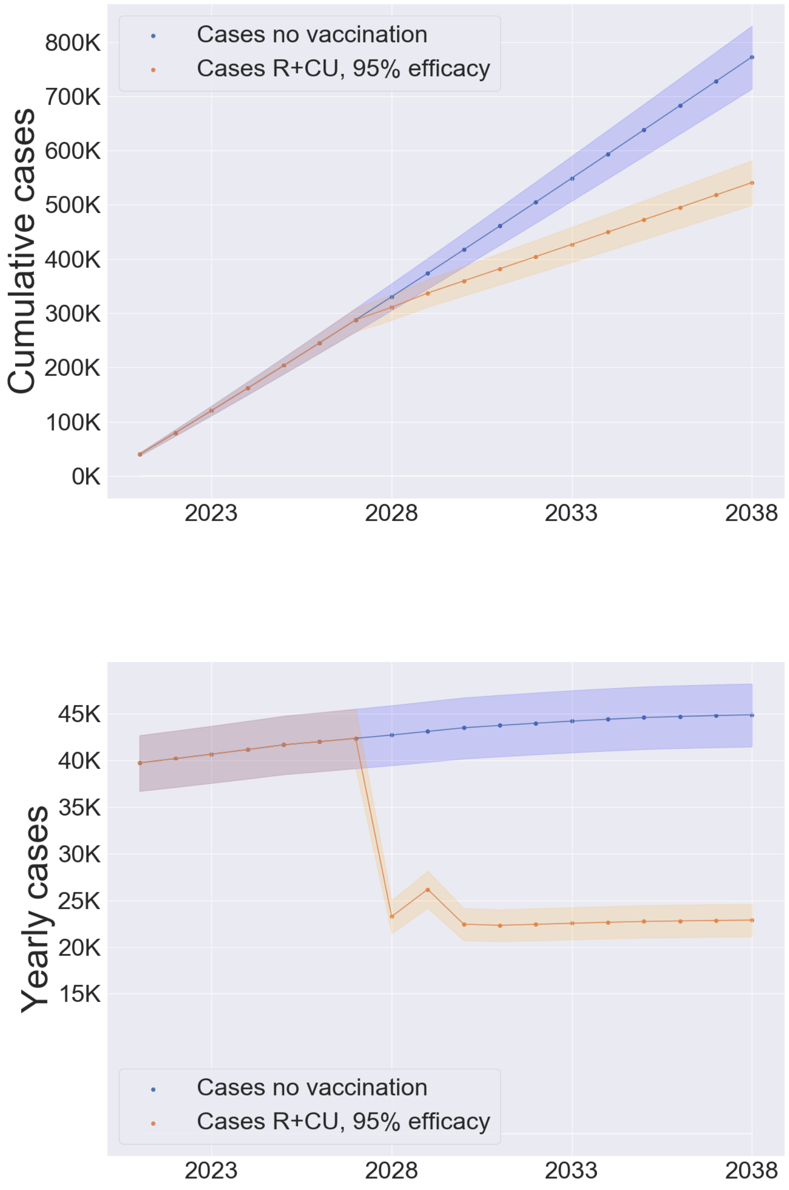

Supplementary Fig. 26 Ethiopia cumulative and yearly iNTS cases: Ethiopia cumulative (top) and yearly (bottom) iNTS cases under the status quo and routine + catch-up vaccination (95% efficacy) scenarios. Shaded areas show the 25th and 75th percentiles, line shows the median over 1000 experiments, samples drawn from uniform distributions over (0.00020,0.00024) for $\beta_{2,n}$ and (0.0080,0.0084) for $\beta_{4,n}$.

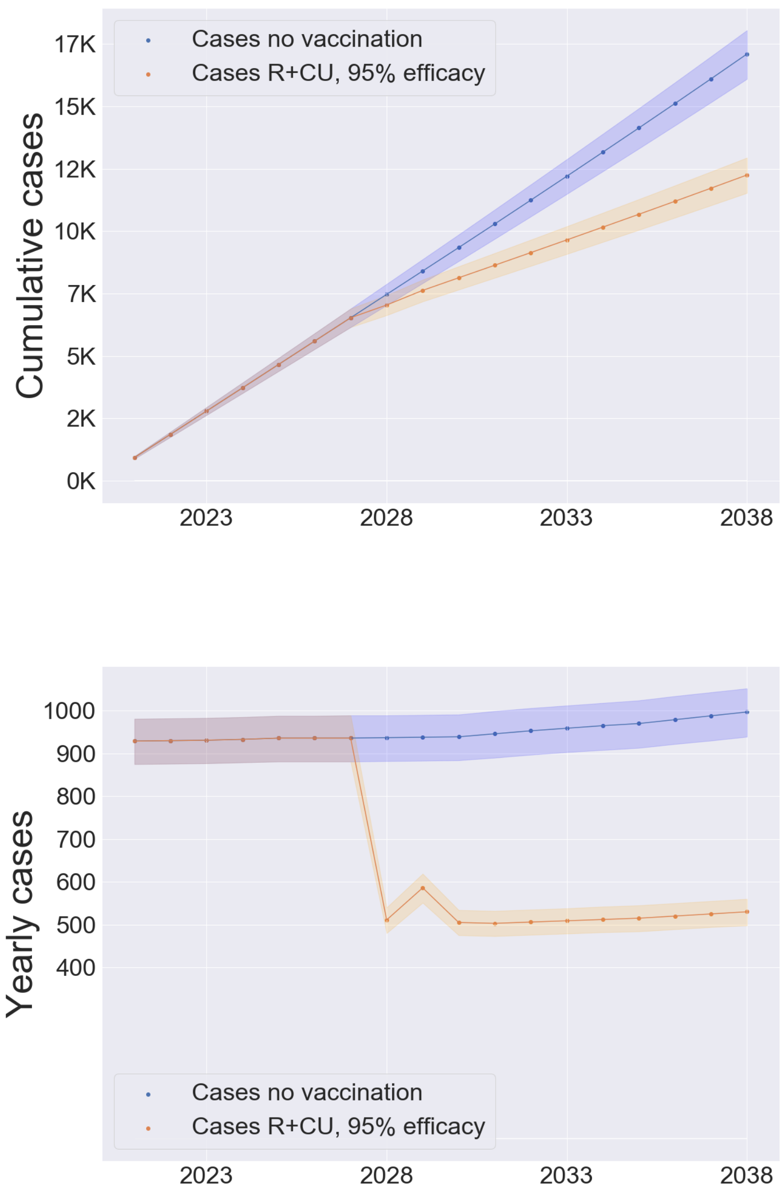

Supplementary Fig. 27 Gabon cumulative and yearly iNTS cases: Gabon cumulative (top) and yearly (bottom) iNTS cases under the status quo and routine + catch-up vaccination (95% efficacy) scenarios. Shaded areas show the 25th and 75th percentiles, line shows the median over 1000 experiments, samples drawn from uniform distributions over (0.00020,0.00024) for $\beta_{2,n}$ and (0.0080,0.0084) for $\beta_{4,n}$.

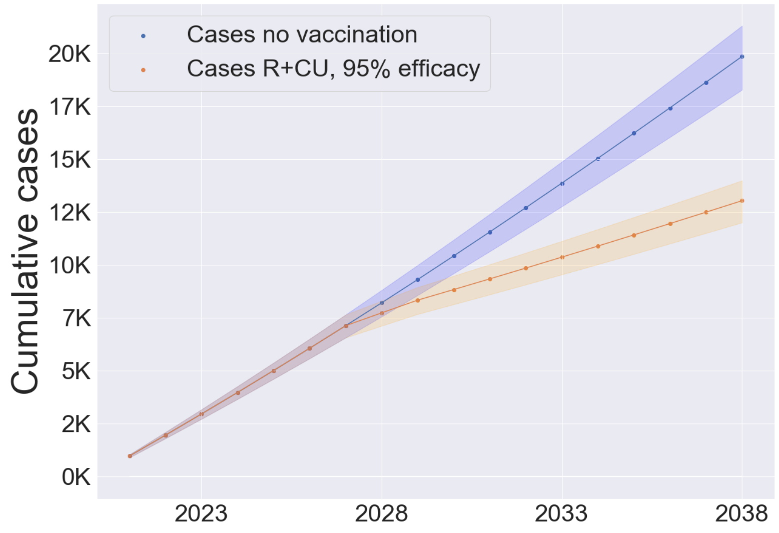

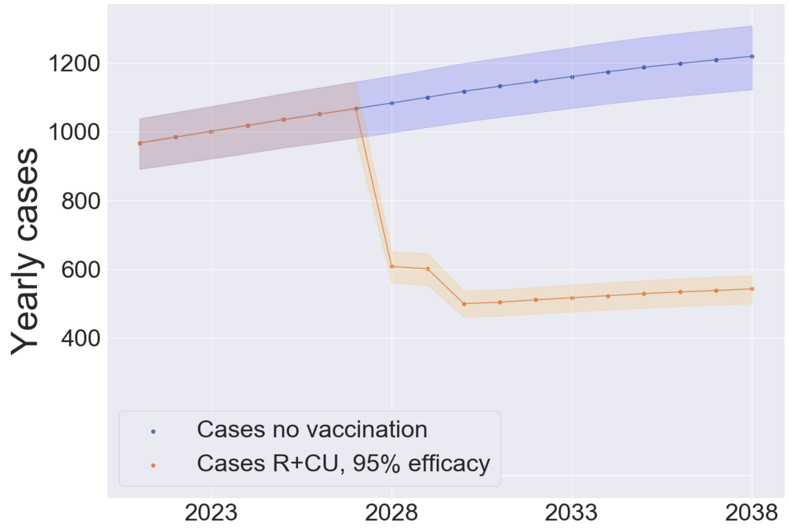

Supplementary Fig. 28 Gambia cumulative and yearly iNTS cases: Gambia cumulative (top) and yearly (bottom) iNTS cases under the status quo and routine + catch-up vaccination (95% efficacy) scenarios. Shaded areas show the 25th and 75th percentiles, line shows the median over 1000 experiments, samples drawn from uniform distributions over (0.00020,0.00024) for $\beta_{2,n}$ and (0.0080,0.0084) for $\beta_{4,n}$.

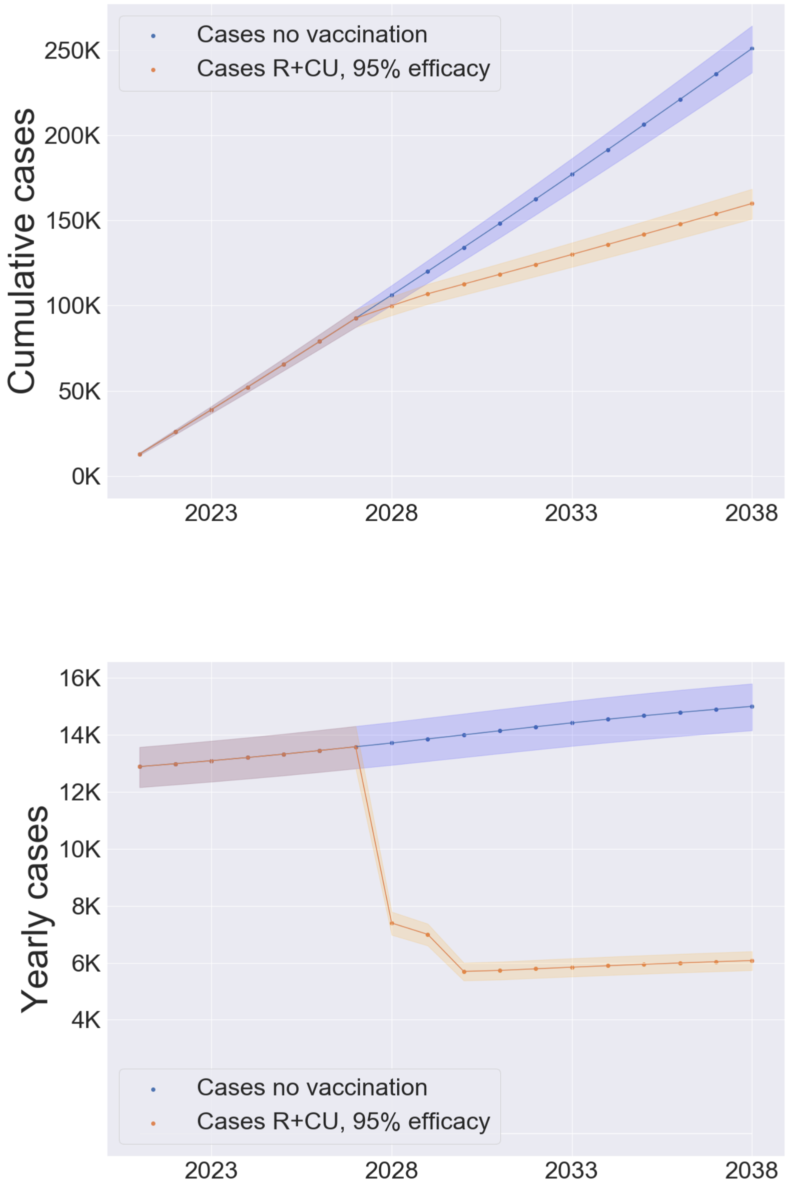

Supplementary Fig. 29 Ghana cumulative and yearly iNTS cases: Ghana cumulative (top) and yearly (bottom) iNTS cases under the status quo and routine + catch-up vaccination (95% efficacy) scenarios. Shaded areas show the 25th and 75th percentiles, line shows the median over 1000 experiments, samples drawn from uniform distributions over (0.00020,0.00024) for $\beta_{2,n}$ and (0.0080,0.0084) for $\beta_{4,n}$.

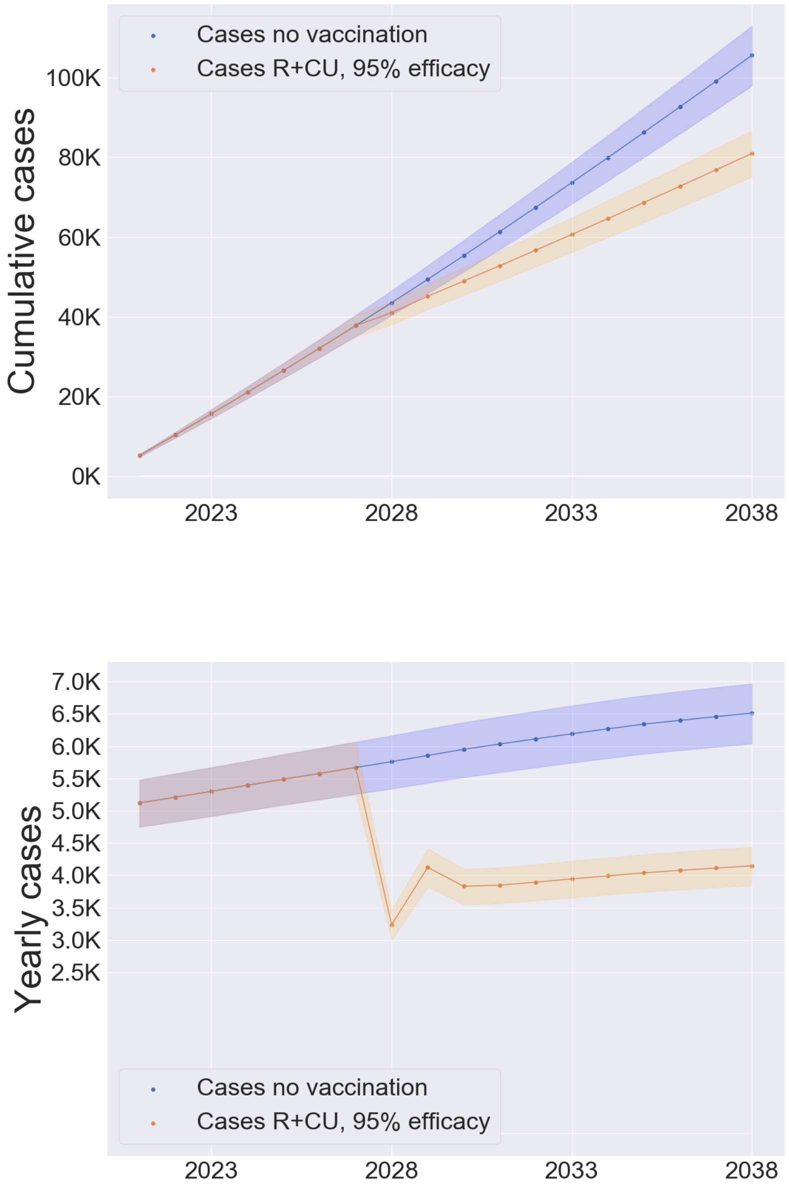

Supplementary Fig. 30 Guinea cumulative and yearly iNTS cases: Guinea cumulative (top) and yearly (bottom) iNTS cases under the status quo and routine + catch-up vaccination (95% efficacy) scenarios. Shaded areas show the 25th and 75th percentiles, line shows the median over 1000 experiments, samples drawn from uniform distributions over (0.00020,0.00024) for $\beta_{2,n}$ and (0.0080,0.0084) for $\beta_{4,n}$.

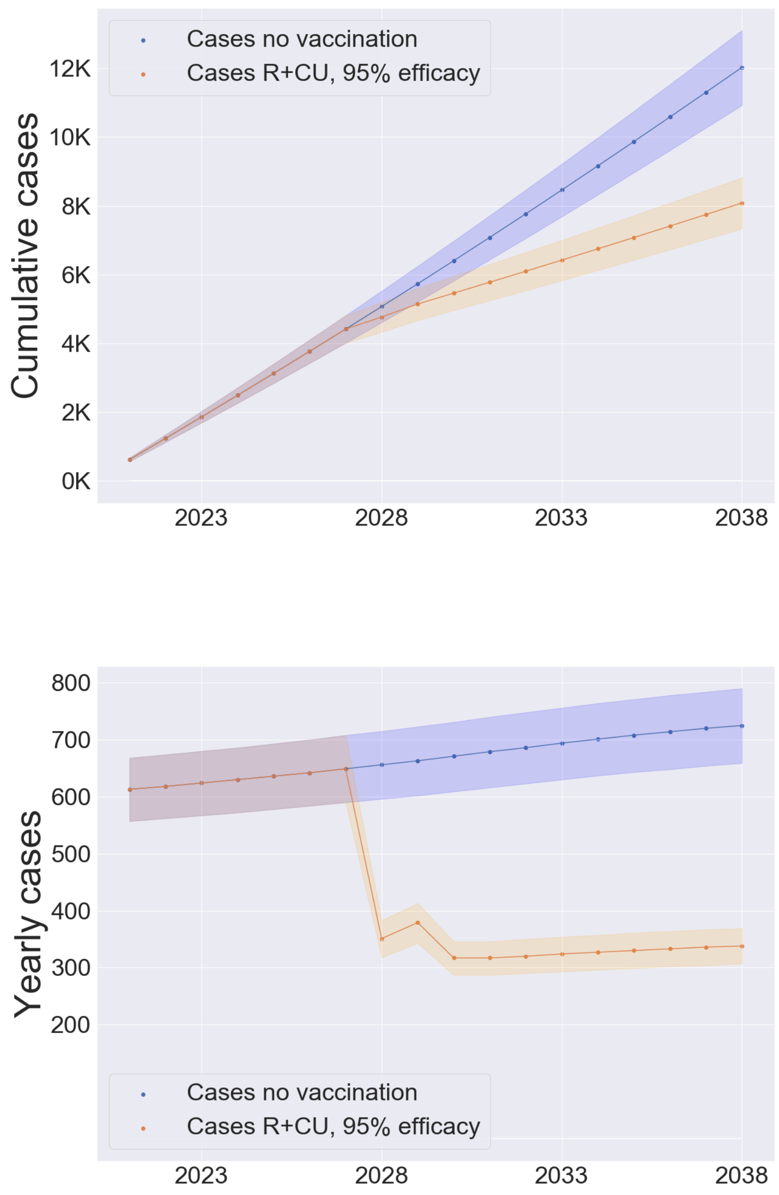

Supplementary Fig. 31 Guinea-Bissau cumulative and yearly iNTS cases: Guinea-Bissau cumulative (top) and yearly (bottom) iNTS cases under the status quo and routine + catch-up vaccination (95% efficacy) scenarios. Shaded areas show the 25th and 75th percentiles, line shows the median over 1000 experiments, samples drawn from uniform distributions over (0.00020,0.00024) for $\beta_{2,n}$ and (0.0080,0.0084) for $\beta_{4,n}$.

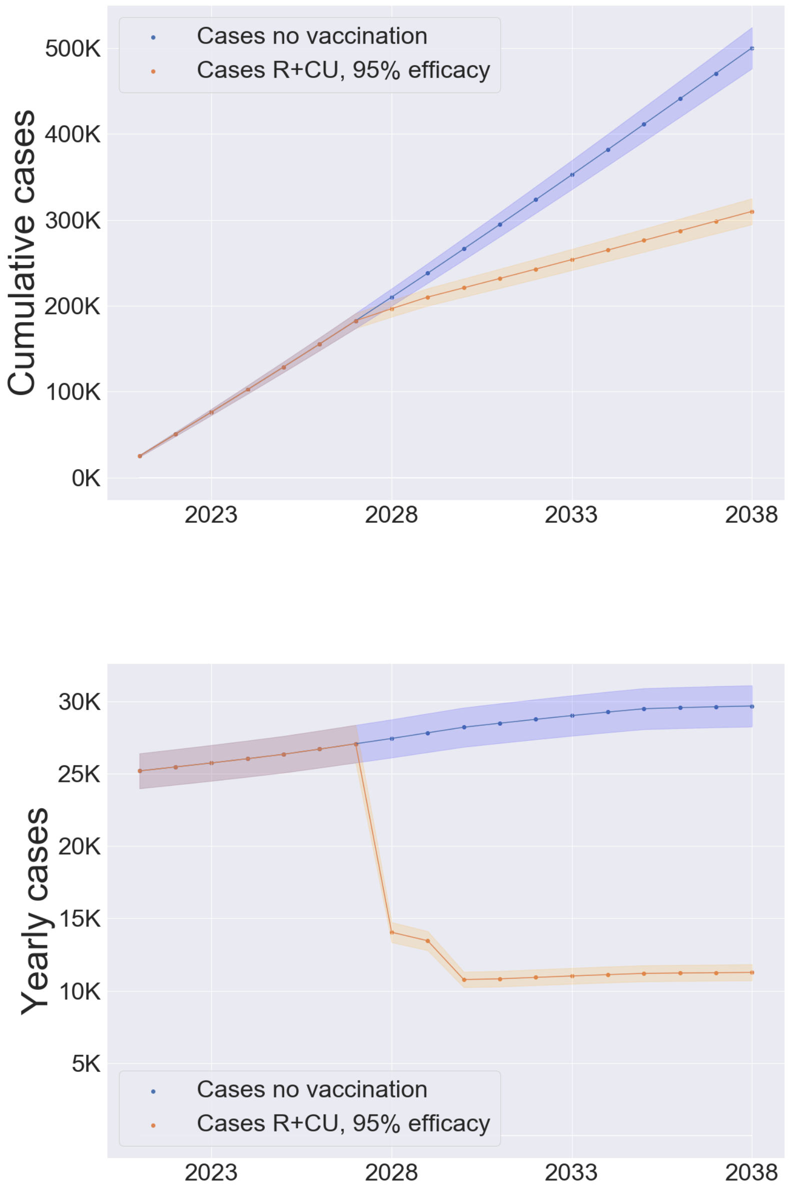

Supplementary Fig. 32 Kenya cumulative and yearly iNTS cases: Kenya cumulative (top) and yearly (bottom) iNTS cases under the status quo and routine + catch-up vaccination (95% efficacy) scenarios. Shaded areas show the 25th and 75th percentiles, line shows the median over 1000 experiments, samples drawn from uniform distributions over (0.00020,0.00024) for $\beta_{2,n}$ and (0.0080,0.0084) for $\beta_{4,n}$.

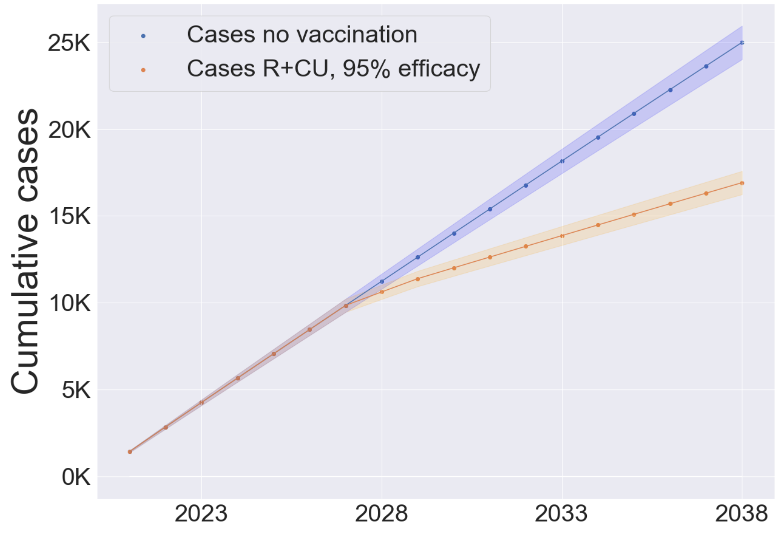

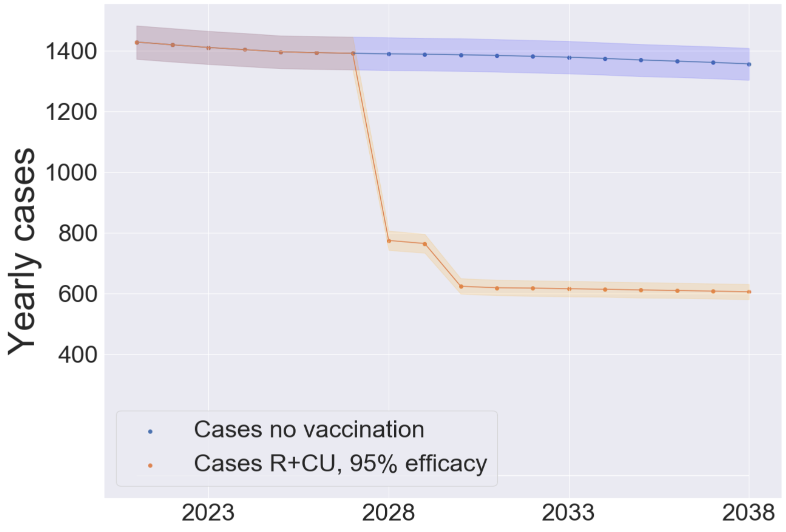

Supplementary Fig. 33 Lesotho cumulative and yearly iNTS cases: Lesotho cumulative (top) and yearly (bottom) iNTS cases under the status quo and routine + catch-up vaccination (95% efficacy) scenarios. Shaded areas show the 25th and 75th percentiles, line shows the median over 1000 experiments, samples drawn from uniform distributions over (0.00020,0.00024) for $\beta_{2,n}$ and (0.0080,0.0084) for $\beta_{4,n}$.

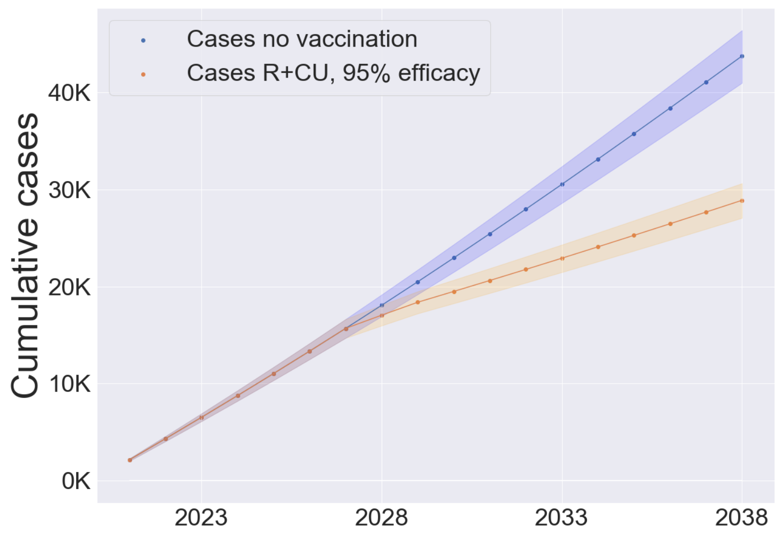

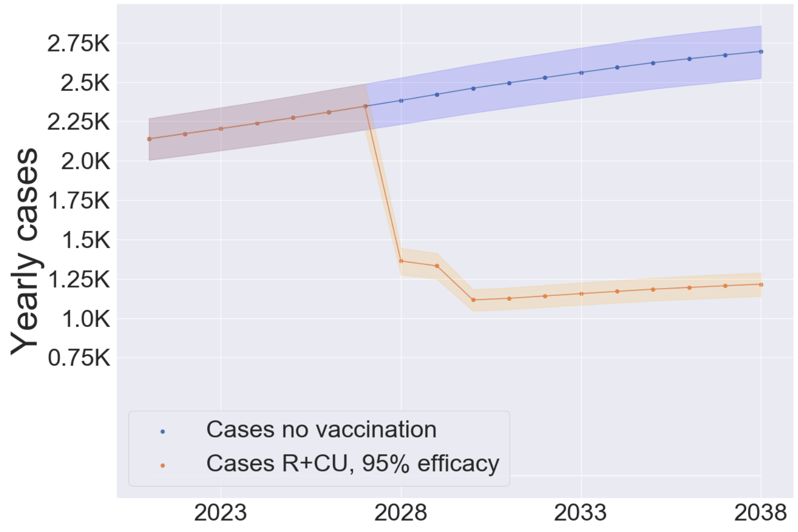

Supplementary Fig. 34 Liberia cumulative and yearly iNTS cases: Liberia cumulative (top) and yearly (bottom) iNTS cases under the status quo and routine + catch-up vaccination (95% efficacy) scenarios. Shaded areas show the 25th and 75th percentiles, line shows the median over 1000 experiments, samples drawn from uniform distributions over (0.00020,0.00024) for $\beta_{2,n}$ and (0.0080,0.0084) for $\beta_{4,n}$.

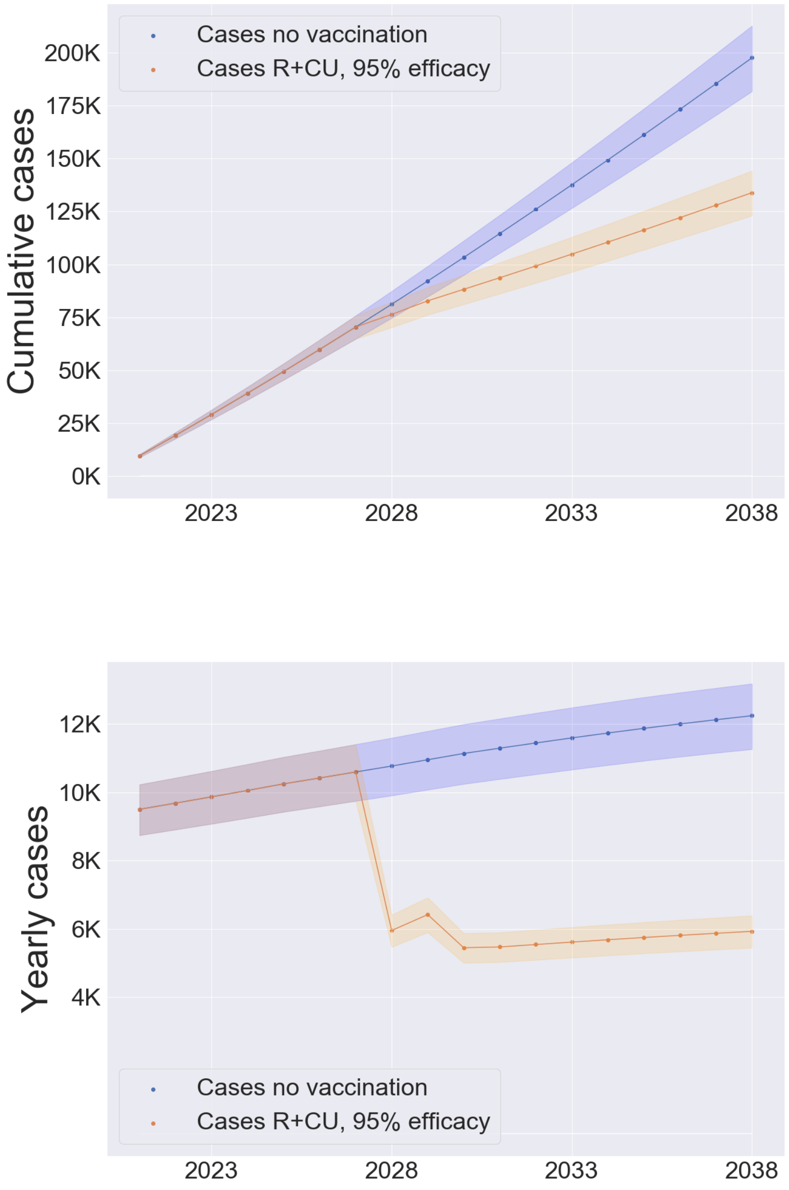

Supplementary Fig. 35 Madagascar cumulative and yearly iNTS cases: Madagascar cumulative (top) and yearly (bottom) iNTS cases under the status quo and routine + catch-up vaccination (95% efficacy) scenarios. Shaded areas show the 25th and 75th percentiles, line shows the median over 1000 experiments, samples drawn from uniform distributions over (0.00020,0.00024) for $\beta_{2,n}$ and (0.0080,0.0084) for $\beta_{4,n}$.

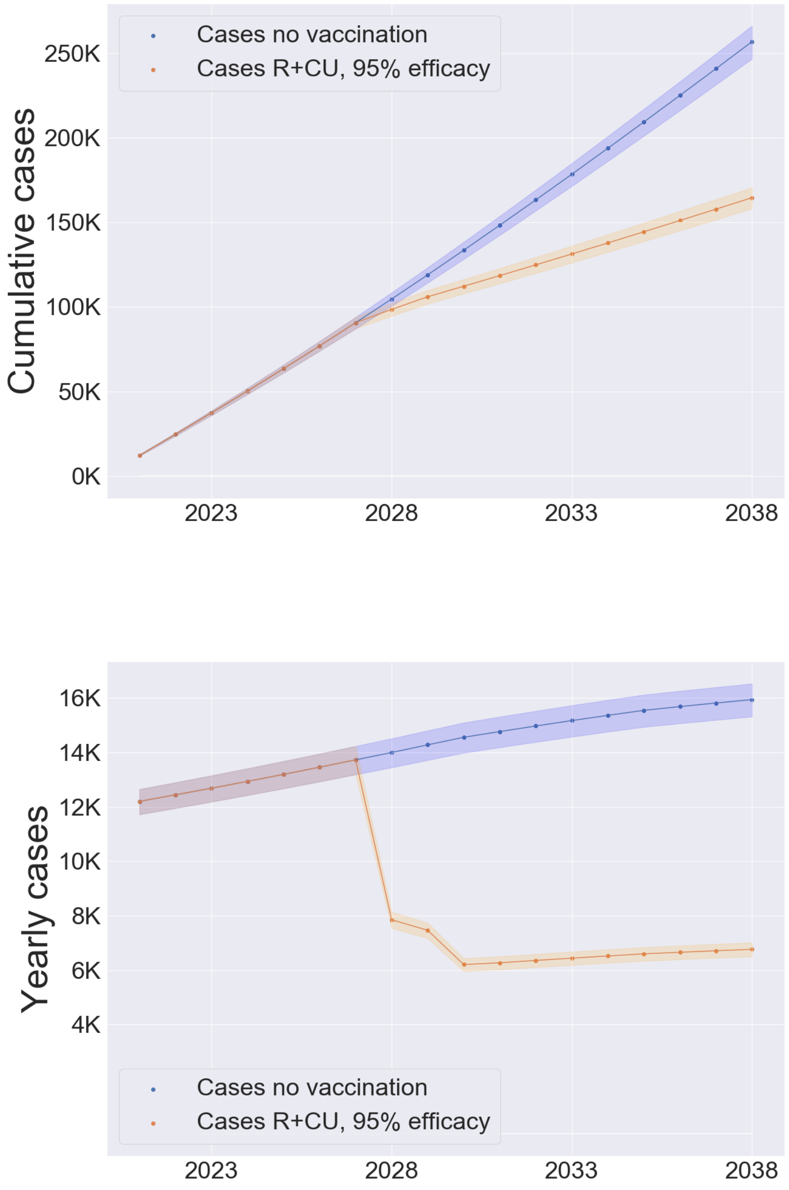

Supplementary Fig. 36 Malawi cumulative and yearly iNTS cases: Malawi cumulative (top) and yearly (bottom) iNTS cases under the status quo and routine + catch-up vaccination (95% efficacy) scenarios. Shaded areas show the 25th and 75th percentiles, line shows the median over 1000 experiments, samples drawn from uniform distributions over (0.00020,0.00024) for $\beta_{2,n}$ and (0.0080,0.0084) for $\beta_{4,n}$.

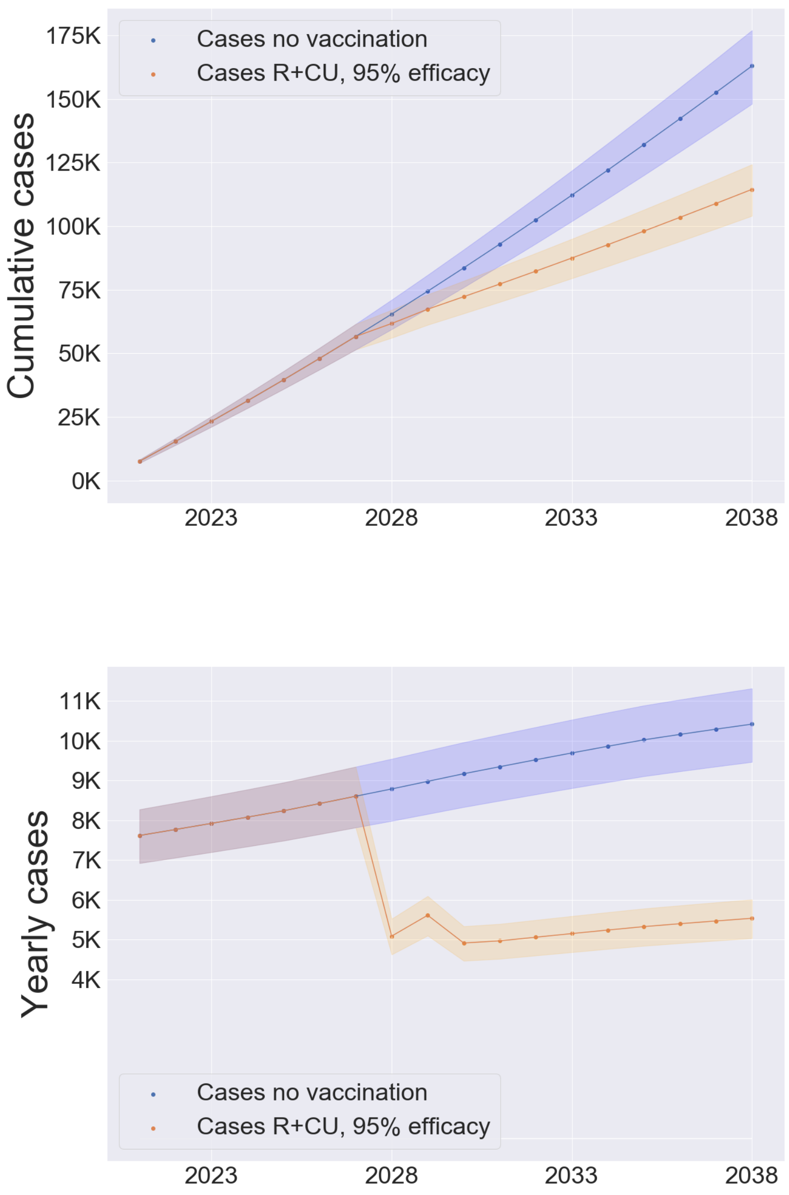

Supplementary Fig. 37 Mali cumulative and yearly iNTS cases: Mali cumulative (top) and yearly (bottom) iNTS cases under the status quo and routine + catch-up vaccination (95% efficacy) scenarios. Shaded areas show the 25th and 75th percentiles, line shows the median over 1000 experiments, samples drawn from uniform distributions over (0.00020,0.00024) for $\beta_{2,n}$ and (0.0080,0.0084) for $\beta_{4,n}$.

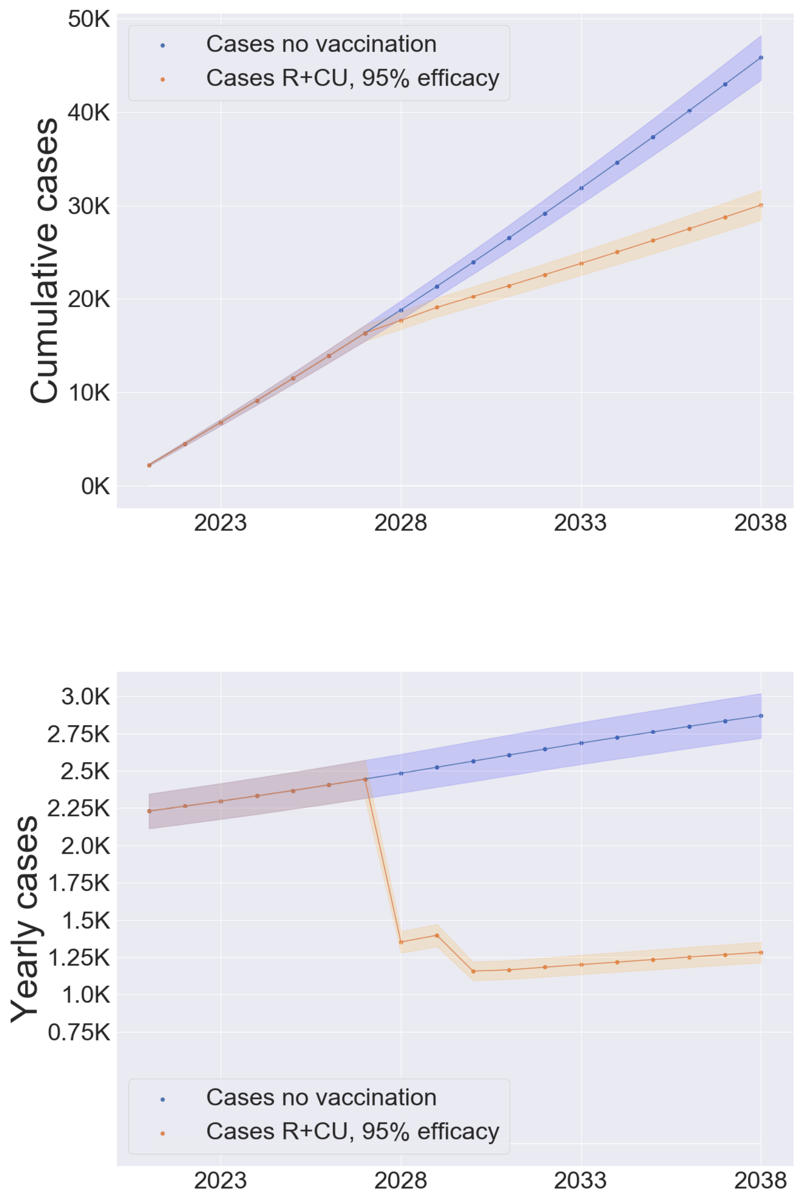

Supplementary Fig. 38 Mauritania cumulative and yearly iNTS cases: Mauritania cumulative (top) and yearly (bottom) iNTS cases under the status quo and routine + catch-up vaccination (95% efficacy) scenarios. Shaded areas show the 25th and 75th percentiles, line shows the median over 1000 experiments, samples drawn from uniform distributions over (0.00020,0.00024) for $\beta_{2,n}$ and (0.0080,0.0084) for $\beta_{4,n}$.

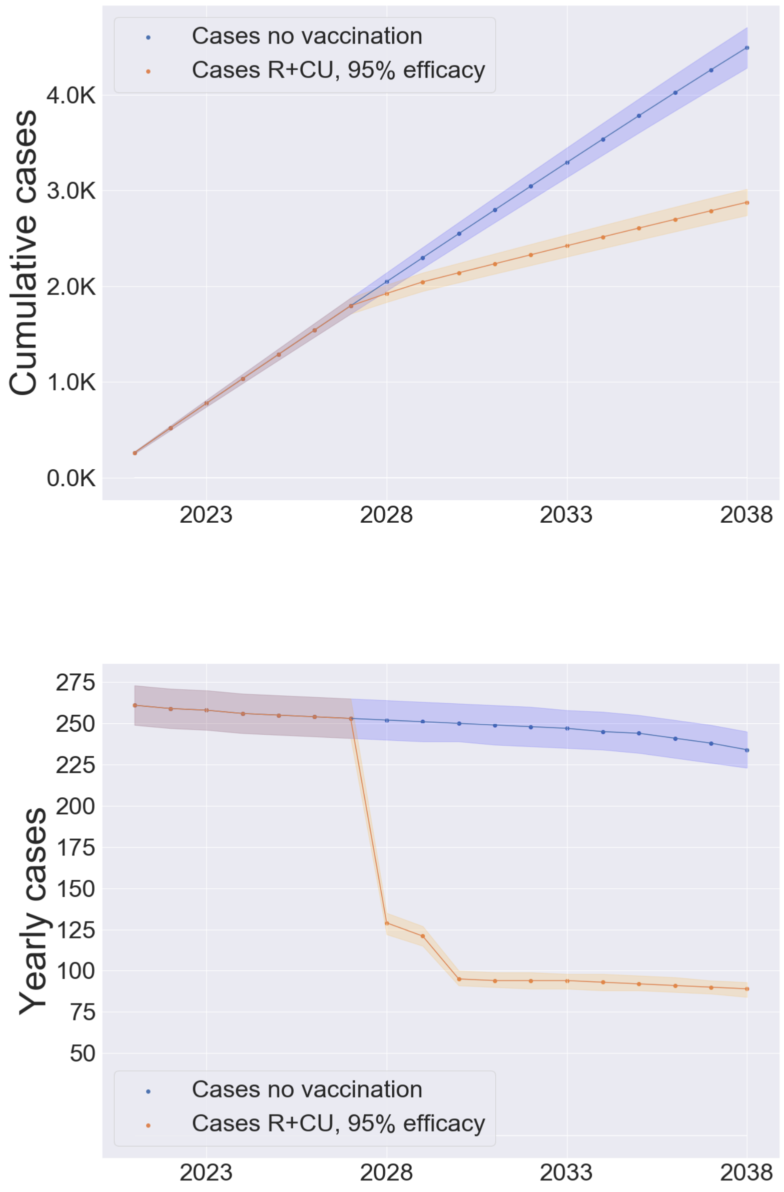

Supplementary Fig. 39 Mauritius cumulative and yearly iNTS cases: Mauritius cumulative (top) and yearly (bottom) iNTS cases under the status quo and routine + catch-up vaccination (95% efficacy) scenarios. Shaded areas show the 25th and 75th percentiles, line shows the median over 1000 experiments, samples drawn from uniform distributions over (0.00020,0.00024) for $\beta_{2,n}$ and (0.0080,0.0084) for $\beta_{4,n}$.

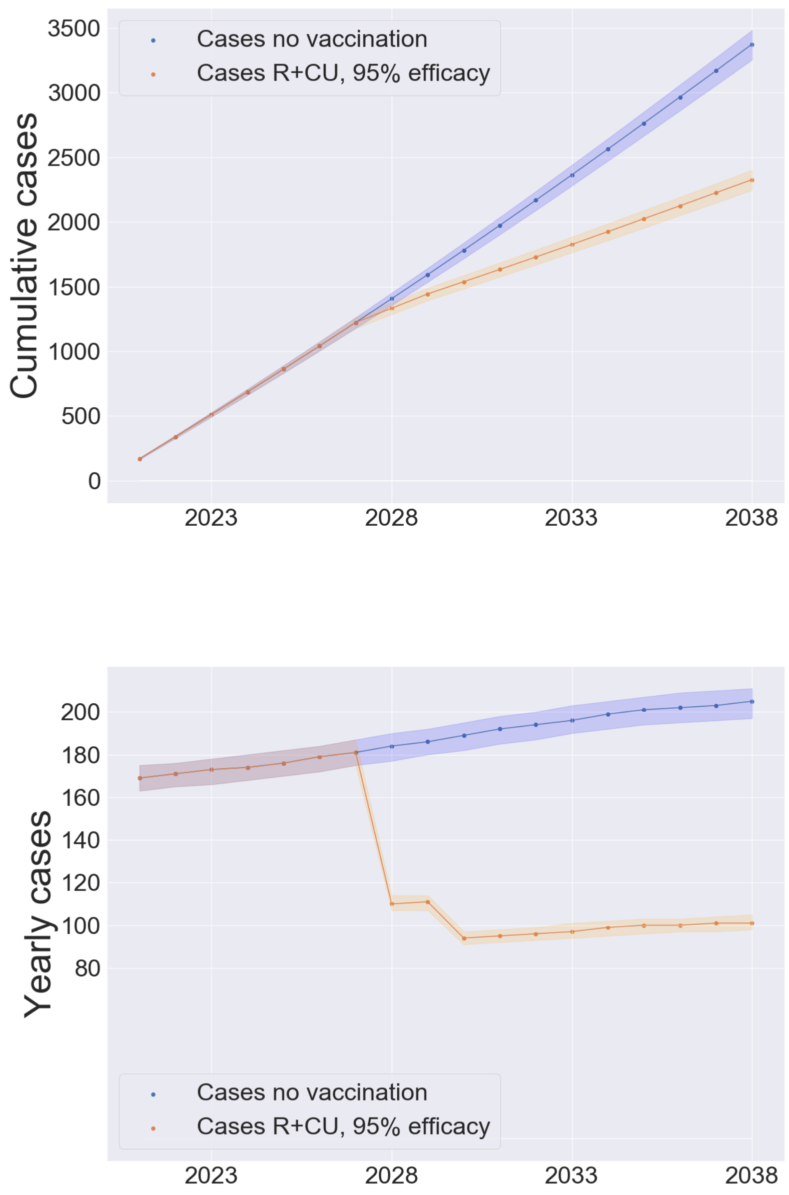

Supplementary Fig. 40 Mayotte cumulative and yearly iNTS cases: Mayotte cumulative (top) and yearly (bottom) iNTS cases under the status quo and routine + catch-up vaccination (95% efficacy) scenarios. Shaded areas show the 25th and 75th percentiles, line shows the median over 1000 experiments, samples drawn from uniform distributions over (0.00020,0.00024) for $\beta_{2,n}$ and (0.0080,0.0084) for $\beta_{4,n}$.

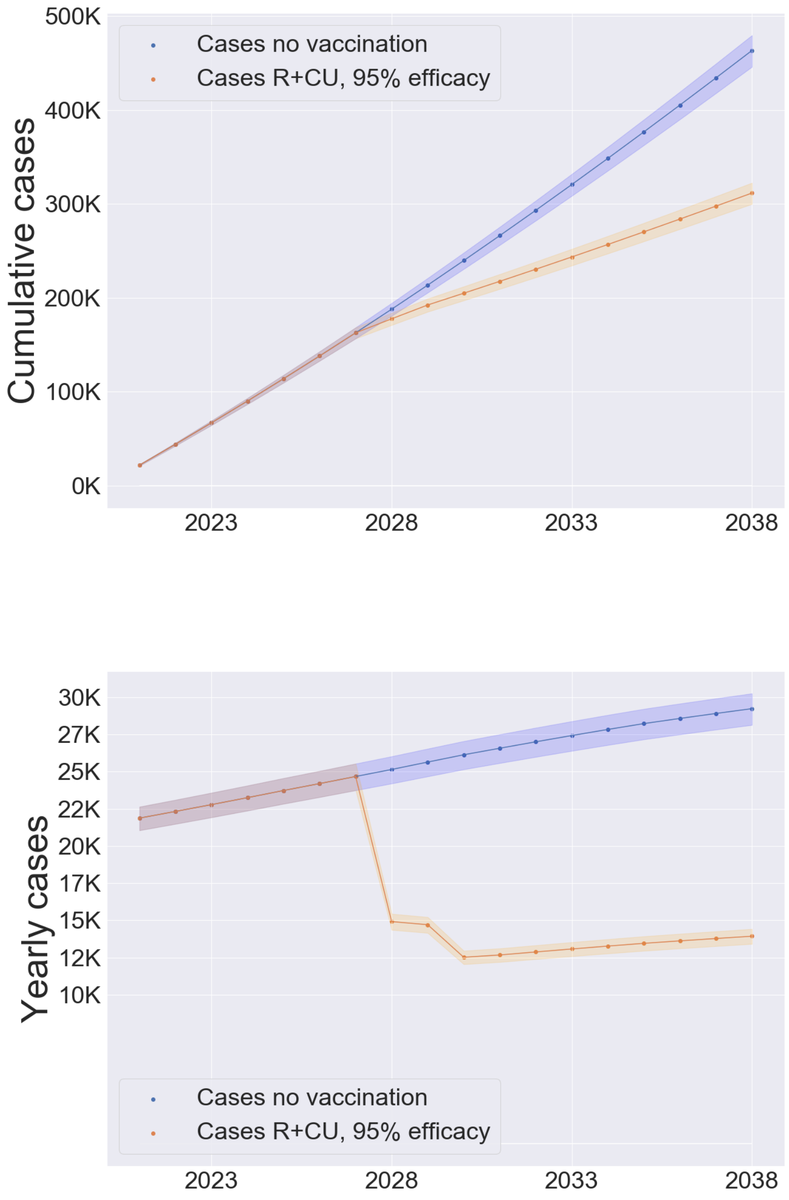

Supplementary Fig. 41 Mozambique cumulative and yearly iNTS cases: Mozambique cumulative (top) and yearly (bottom) iNTS cases under the status quo and routine + catch-up vaccination (95% efficacy) scenarios. Shaded areas show the 25th and 75th percentiles, line shows the median over 1000 experiments, samples drawn from uniform distributions over (0.00020,0.00024) for $\beta_{2,n}$ and (0.0080,0.0084) for $\beta_{4,n}$.

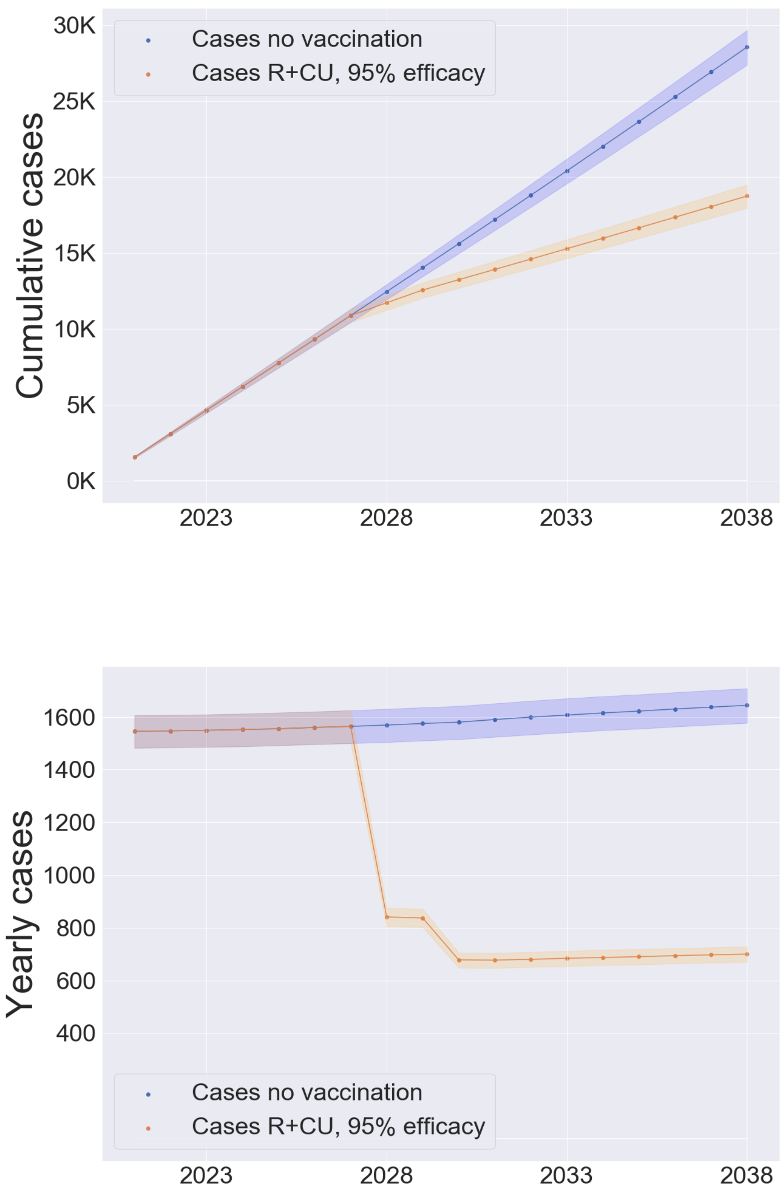

Supplementary Fig. 42 Namibia cumulative and yearly iNTS cases: Namibia cumulative (top) and yearly (bottom) iNTS cases under the status quo and routine + catch-up vaccination (95% efficacy) scenarios. Shaded areas show the 25th and 75th percentiles, line shows the median over 1000 experiments, samples drawn from uniform distributions over (0.00020,0.00024) for $\beta_{2,n}$ and (0.0080,0.0084) for $\beta_{4,n}$.

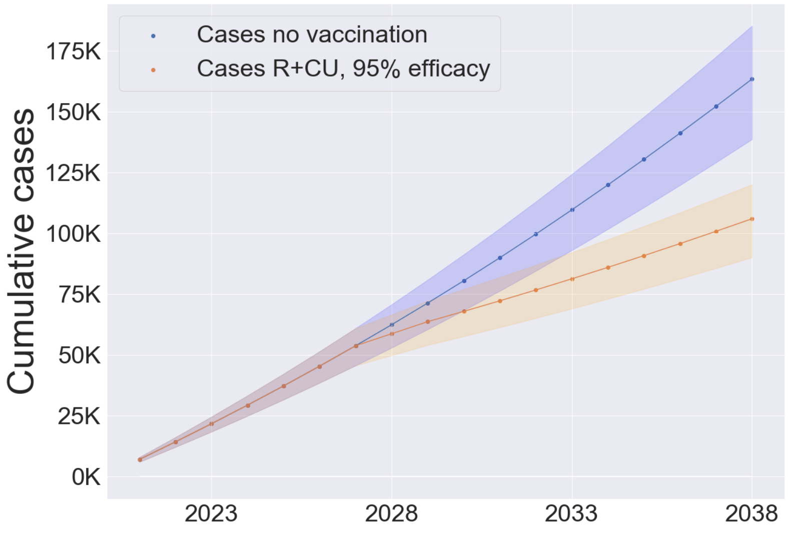

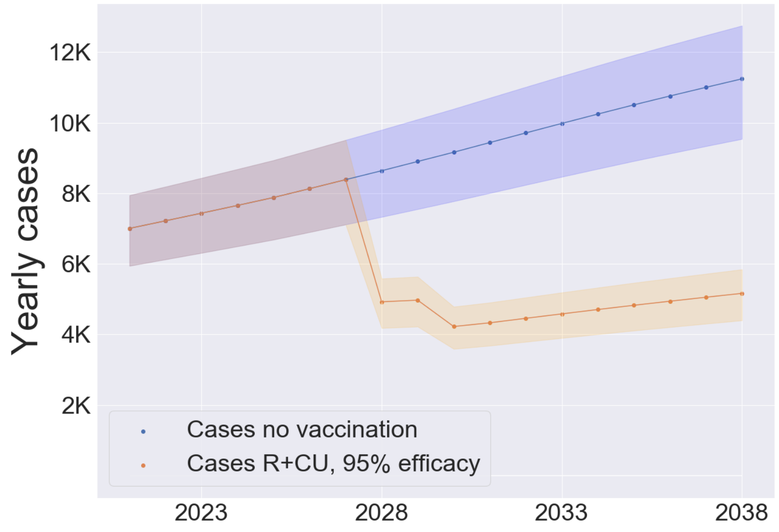

Supplementary Fig. 43 Niger cumulative and yearly iNTS cases: Niger cumulative (top) and yearly (bottom) iNTS cases under the status quo and routine + catch-up vaccination (95% efficacy) scenarios. Shaded areas show the 25th and 75th percentiles, line shows the median over 1000 experiments, samples drawn from uniform distributions over (0.00020,0.00024) for $\beta_{2,n}$ and (0.0080,0.0084) for $\beta_{4,n}$.

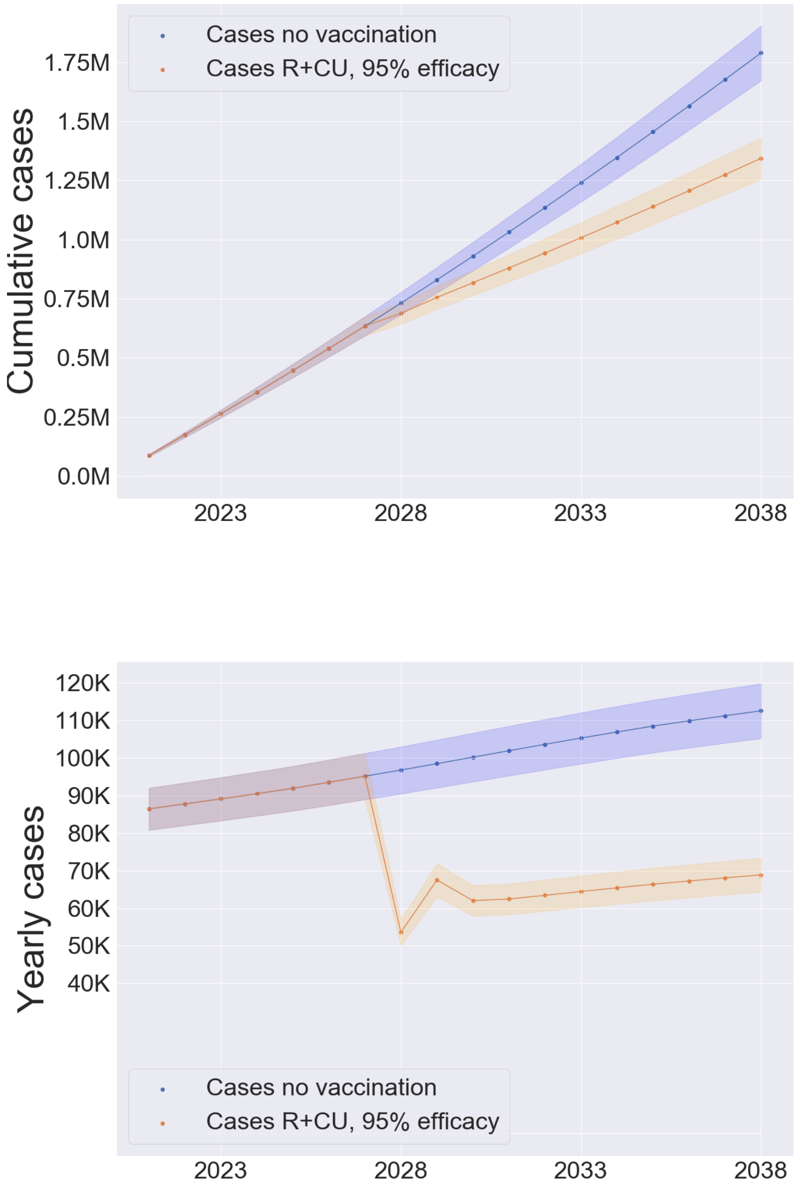

Supplementary Fig. 44 Nigeria cumulative and yearly iNTS cases: Nigeria cumulative (top) and yearly (bottom) iNTS cases under the status quo and routine + catch-up vaccination (95% efficacy) scenarios. Shaded areas show the 25th and 75th percentiles, line shows the median over 1000 experiments, samples drawn from uniform distributions over (0.00020,0.00024) for $\beta_{2,n}$ and (0.0080,0.0084) for $\beta_{4,n}$.

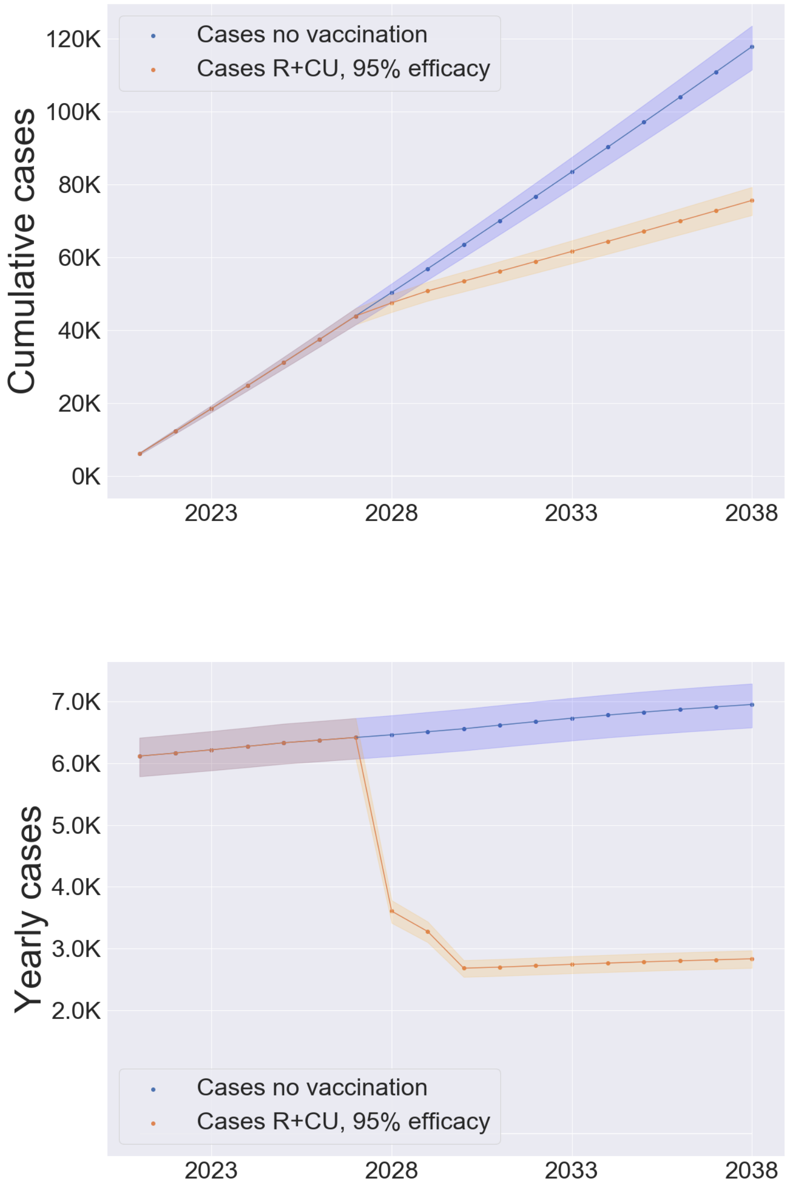

Supplementary Fig. 45 Rwanda cumulative and yearly iNTS cases: Rwanda cumulative (top) and yearly (bottom) iNTS cases under the status quo and routine + catch-up vaccination (95% efficacy) scenarios. Shaded areas show the 25th and 75th percentiles, line shows the median over 1000 experiments, samples drawn from uniform distributions over (0.00020,0.00024) for $\beta_{2,n}$ and (0.0080,0.0084) for $\beta_{4,n}$.

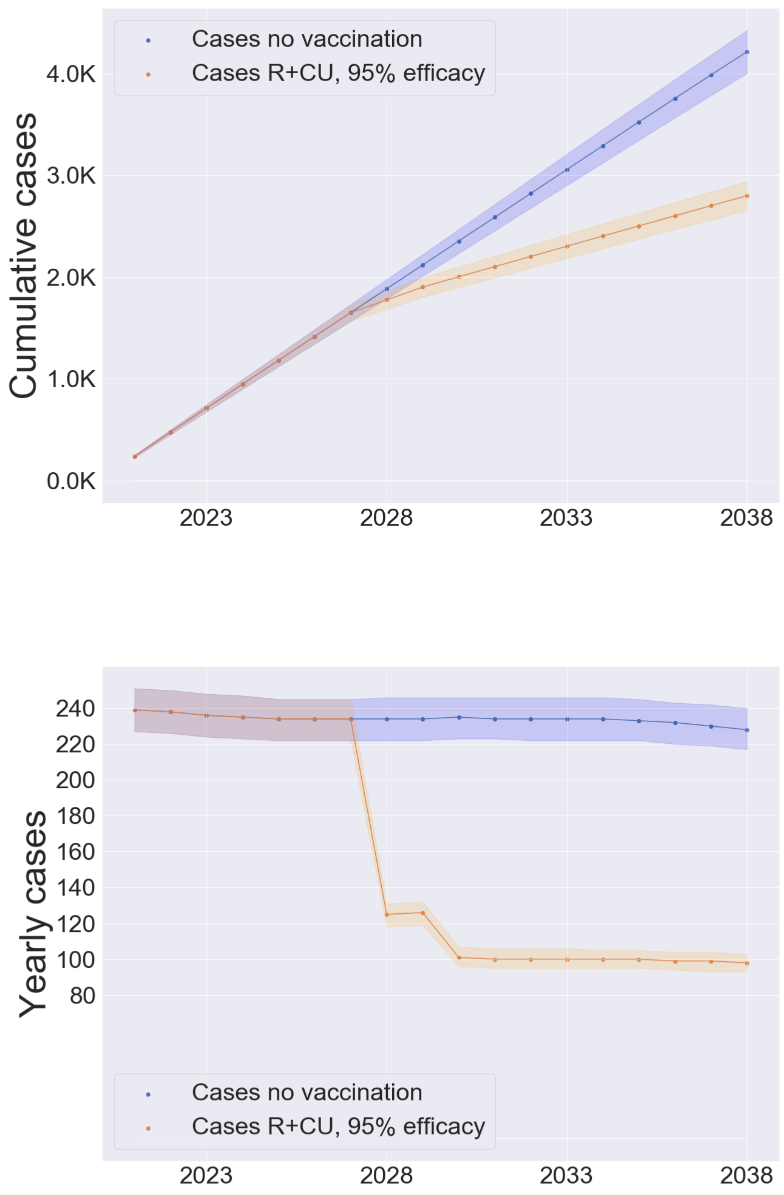

Supplementary Fig. 46 Réunion cumulative and yearly iNTS cases: Réunion cumulative (top) and yearly (bottom) iNTS cases under the status quo and routine + catch-up vaccination (95% efficacy) scenarios. Shaded areas show the 25th and 75th percentiles, line shows the median over 1000 experiments, samples drawn from uniform distributions over (0.00020,0.00024) for $\beta_{2,n}$ and (0.0080,0.0084) for $\beta_{4,n}$.

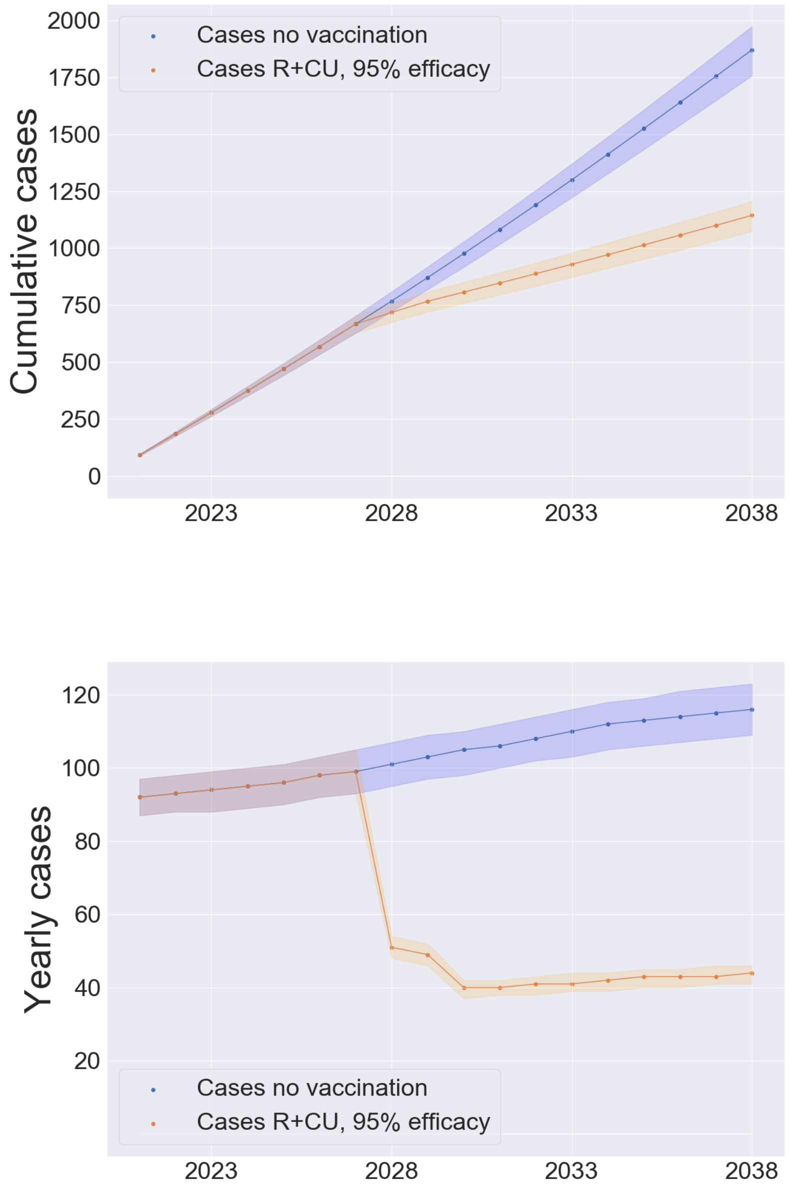

Supplementary Fig. 47 Sao Tome and Principe cumulative and yearly iNTS cases: Sao Tome and Principe cumulative (top) and yearly (bottom) iNTS cases under the status quo and routine + catch-up vaccination (95% efficacy) scenarios. Shaded areas show the 25th and 75th percentiles, line shows the median over 1000 experiments, samples drawn from uniform distributions over (0.00020,0.00024) for $\beta_{2,n}$ and (0.0080,0.0084) for $\beta_{4,n}$.

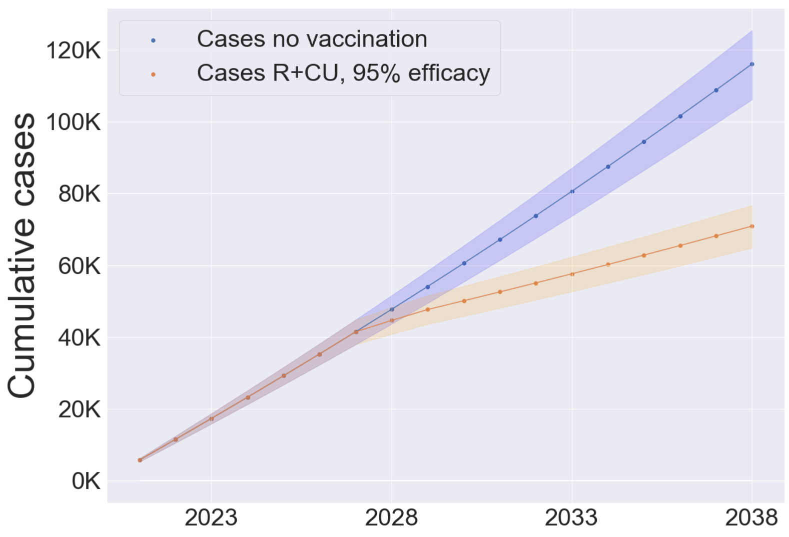

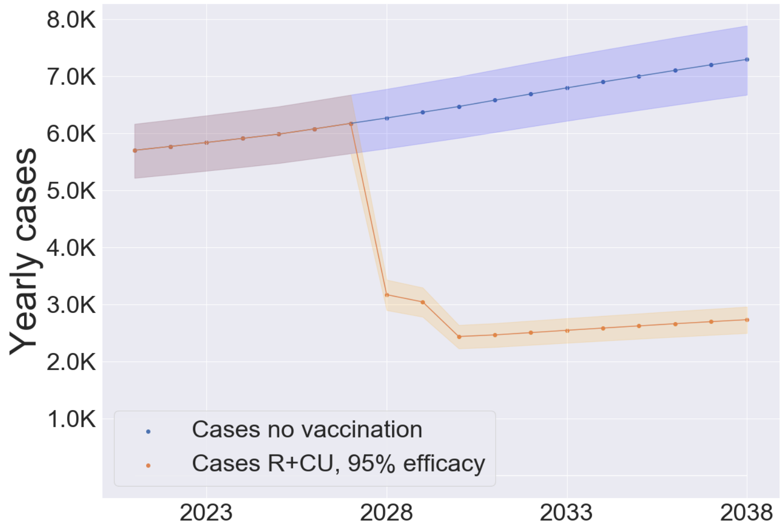

Supplementary Fig. 48 Senegal cumulative and yearly iNTS cases: Senegal cumulative (top) and yearly (bottom) iNTS cases under the status quo and routine + catch-up vaccination (95% efficacy) scenarios. Shaded areas show the 25th and 75th percentiles, line shows the median over 1000 experiments, samples drawn from uniform distributions over (0.00020,0.00024) for $\beta_{2,n}$ and (0.0080,0.0084) for $\beta_{4,n}$.

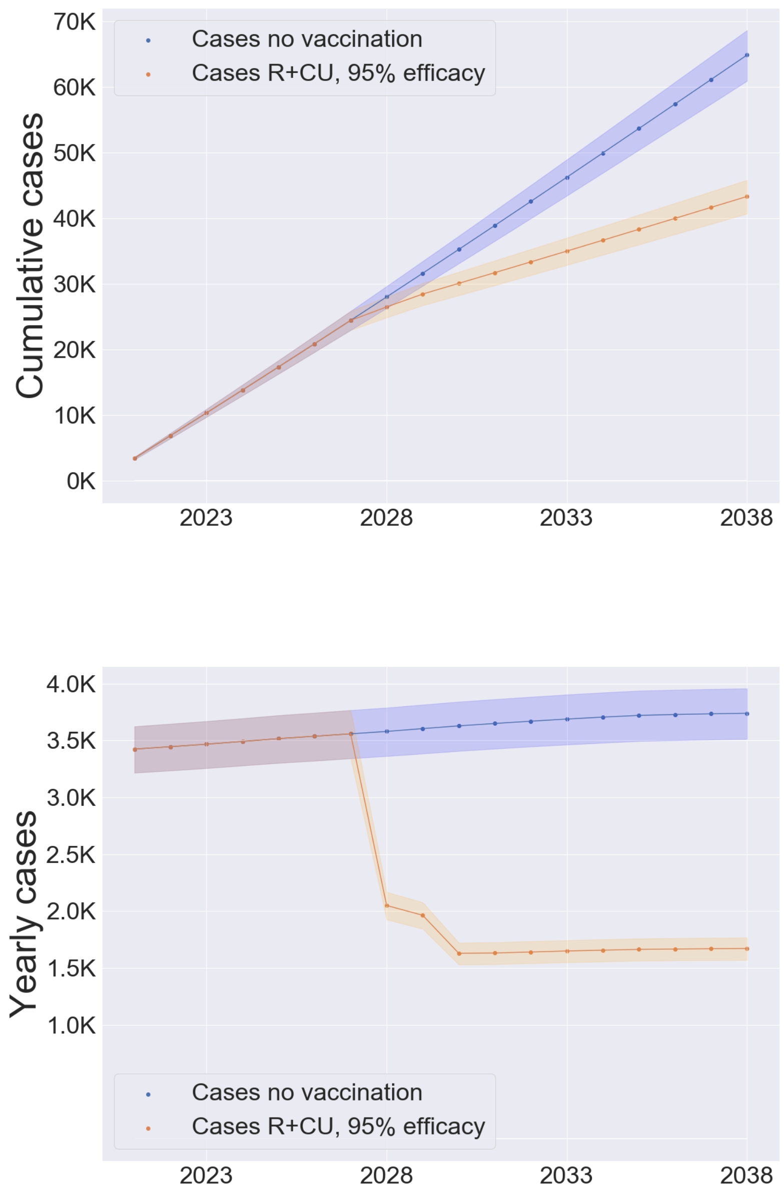

Supplementary Fig. 49 Sierra Leone cumulative and yearly iNTS cases: Sierra Leone cumulative (top) and yearly (bottom) iNTS cases under the status quo and routine + catch-up vaccination (95% efficacy) scenarios. Shaded areas show the 25th and 75th percentiles, line shows the median over 1000 experiments, samples drawn from uniform distributions over (0.00020,0.00024) for $\beta_{2,n}$ and (0.0080,0.0084) for $\beta_{4,n}$.

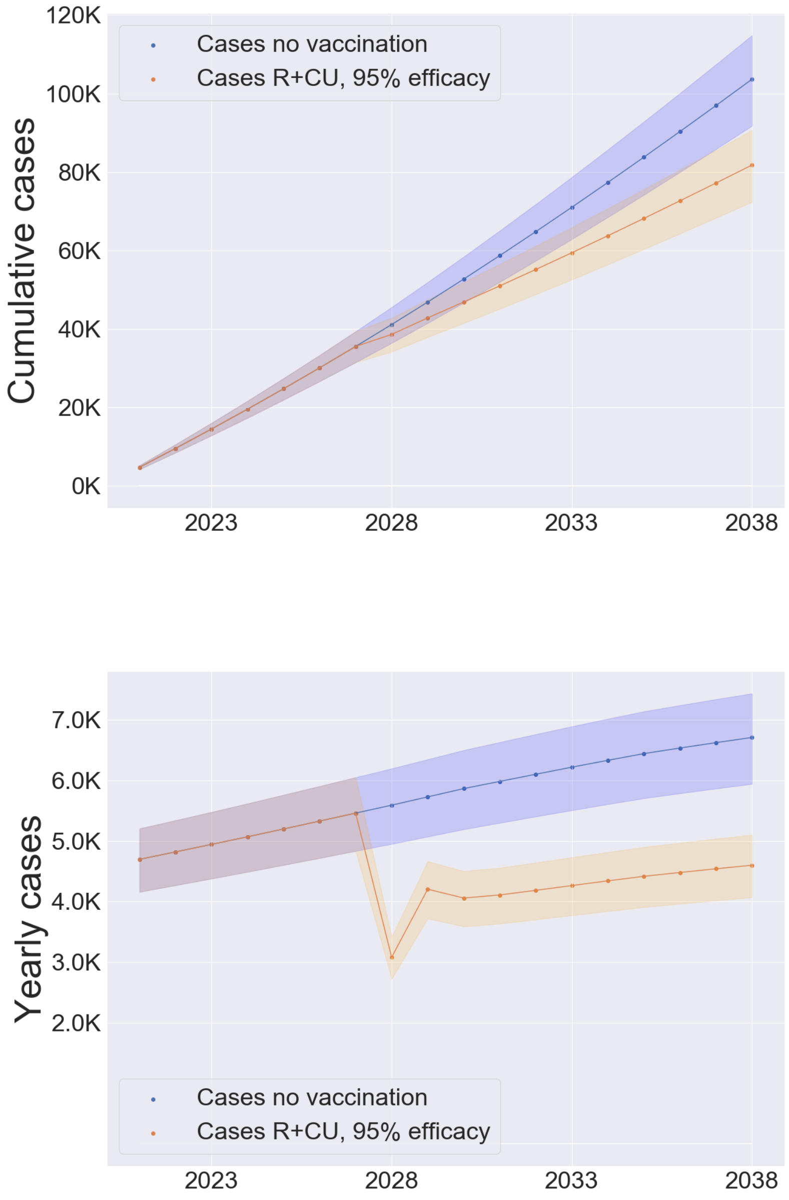

Supplementary Fig. 50 Somalia cumulative and yearly iNTS cases: Somalia cumulative (top) and yearly (bottom) iNTS cases under the status quo and routine + catch-up vaccination (95% efficacy) scenarios. Shaded areas show the 25th and 75th percentiles, line shows the median over 1000 experiments, samples drawn from uniform distributions over (0.00020,0.00024) for $\beta_{2,n}$ and (0.0080,0.0084) for $\beta_{4,n}$.

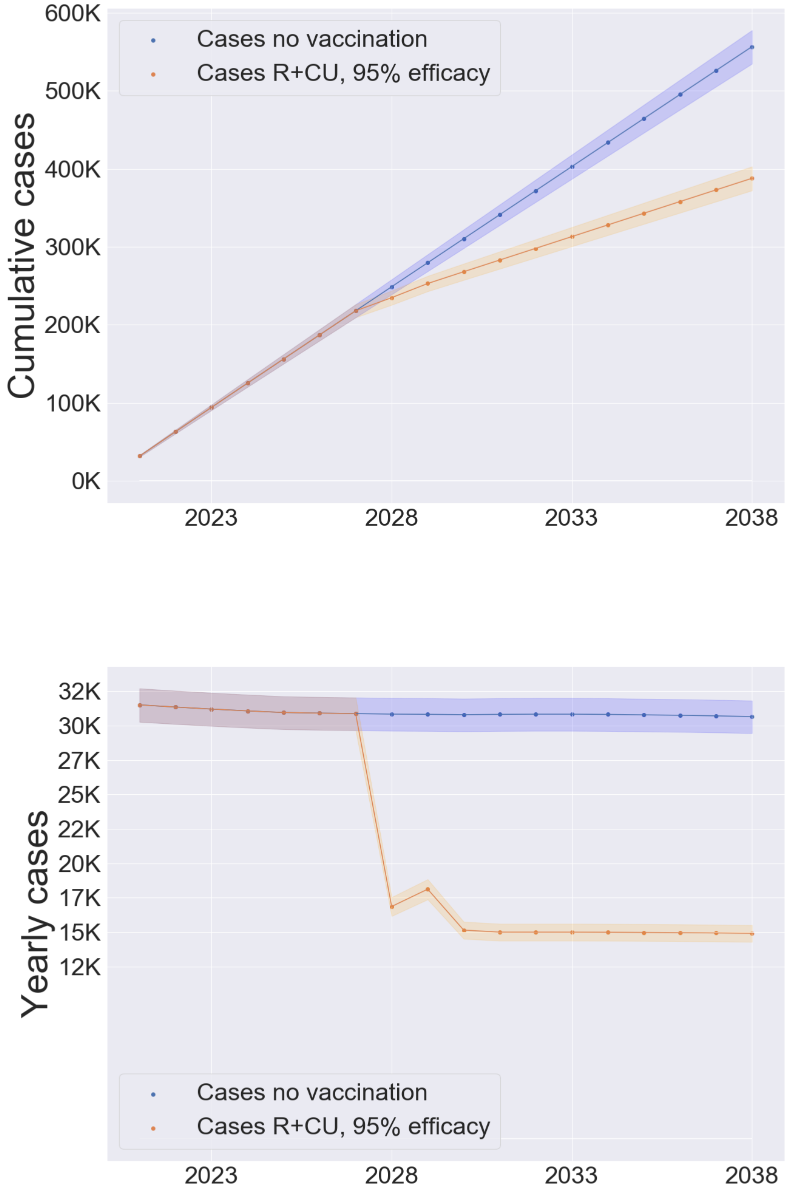

Supplementary Fig. 51 South Africa cumulative and yearly iNTS cases: South Africa cumulative (top) and yearly (bottom) iNTS cases under the status quo and routine + catch-up vaccination (95% efficacy) scenarios. Shaded areas show the 25th and 75th percentiles, line shows the median over 1000 experiments, samples drawn from uniform distributions over (0.00020,0.00024) for $\beta_{2,n}$ and (0.0080,0.0084) for $\beta_{4,n}$.

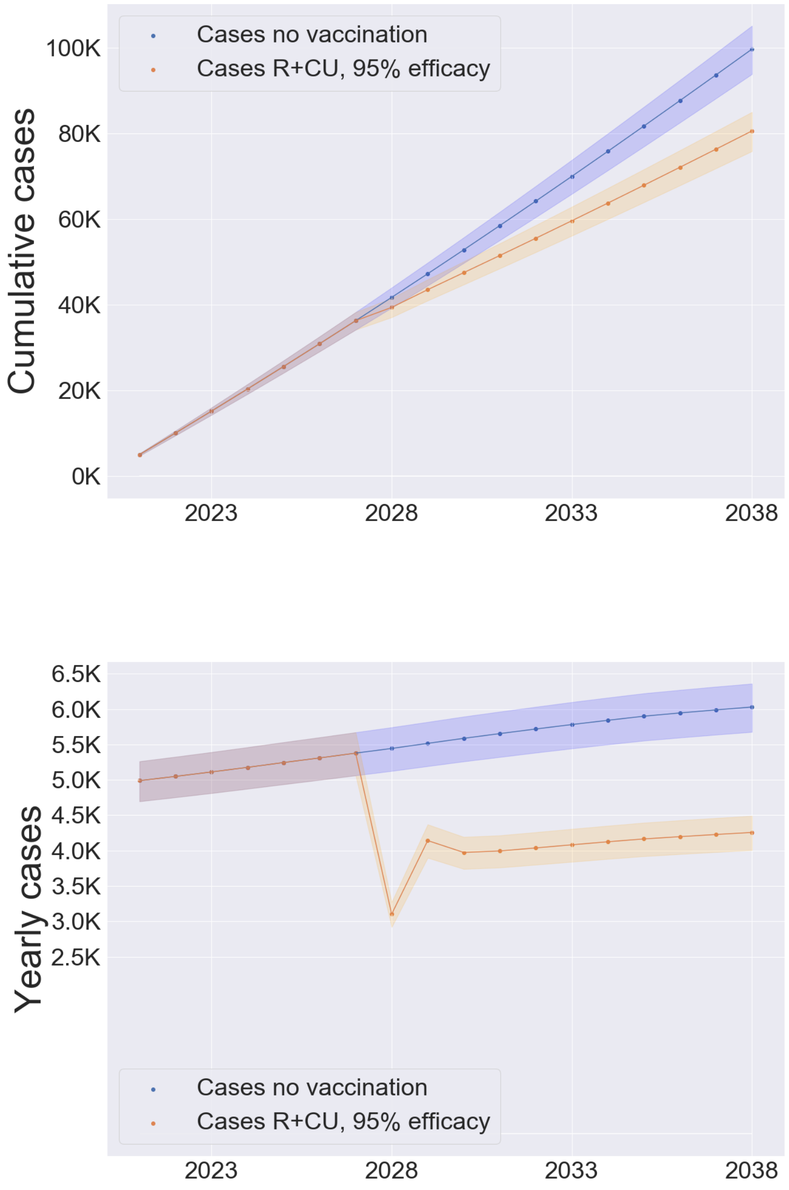

Supplementary Fig. 52 South Sudan cumulative and yearly iNTS cases: South Sudan cumulative (top) and yearly (bottom) iNTS cases under the status quo and routine + catch-up vaccination (95% efficacy) scenarios. Shaded areas show the 25th and 75th percentiles, line shows the median over 1000 experiments, samples drawn from uniform distributions over (0.00020,0.00024) for $\beta_{2,n}$ and (0.0080,0.0084) for $\beta_{4,n}$.

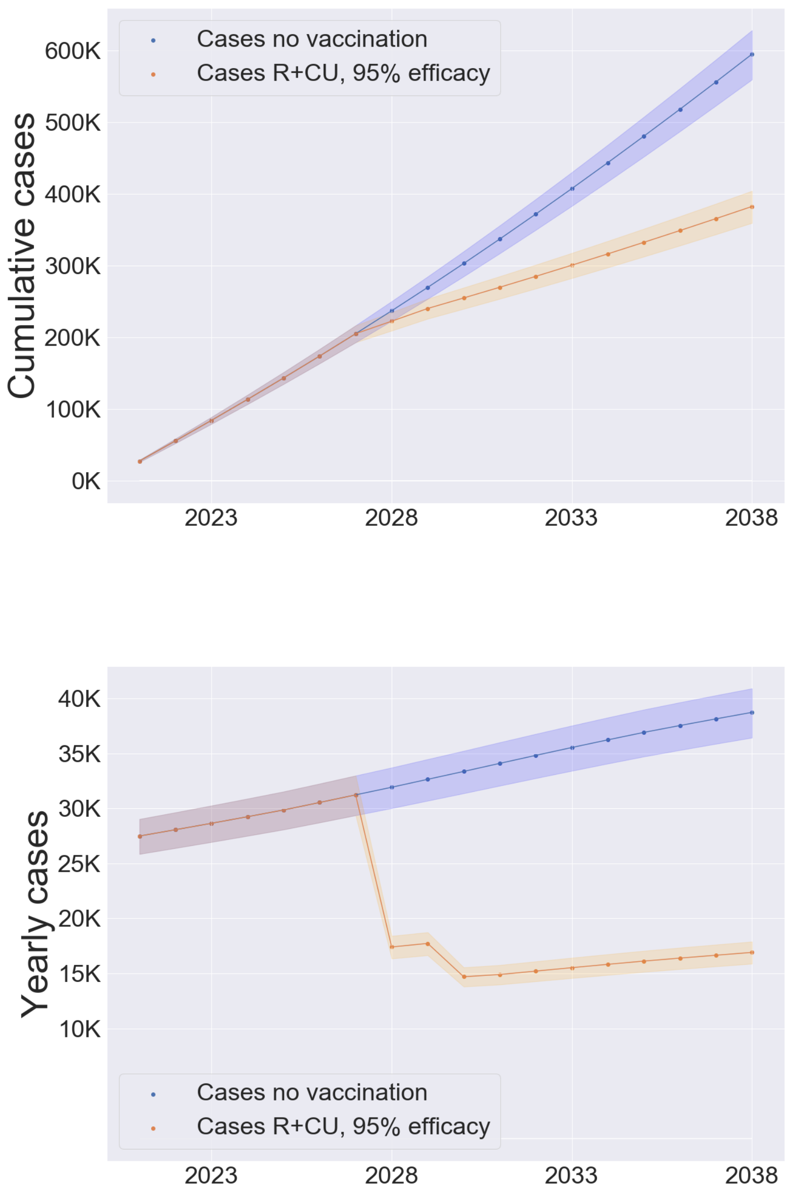

Supplementary Fig. 53 Tanzania cumulative and yearly iNTS cases: Tanzania (United Republic of) cumulative (top) and yearly (bottom) iNTS cases under the status quo and routine + catch-up vaccination (95% efficacy) scenarios. Shaded areas show the 25th and 75th percentiles, line shows the median over 1000 experiments, samples drawn from uniform distributions over (0.00020,0.00024) for $\beta_{2,n}$ and (0.0080,0.0084) for $\beta_{4,n}$.

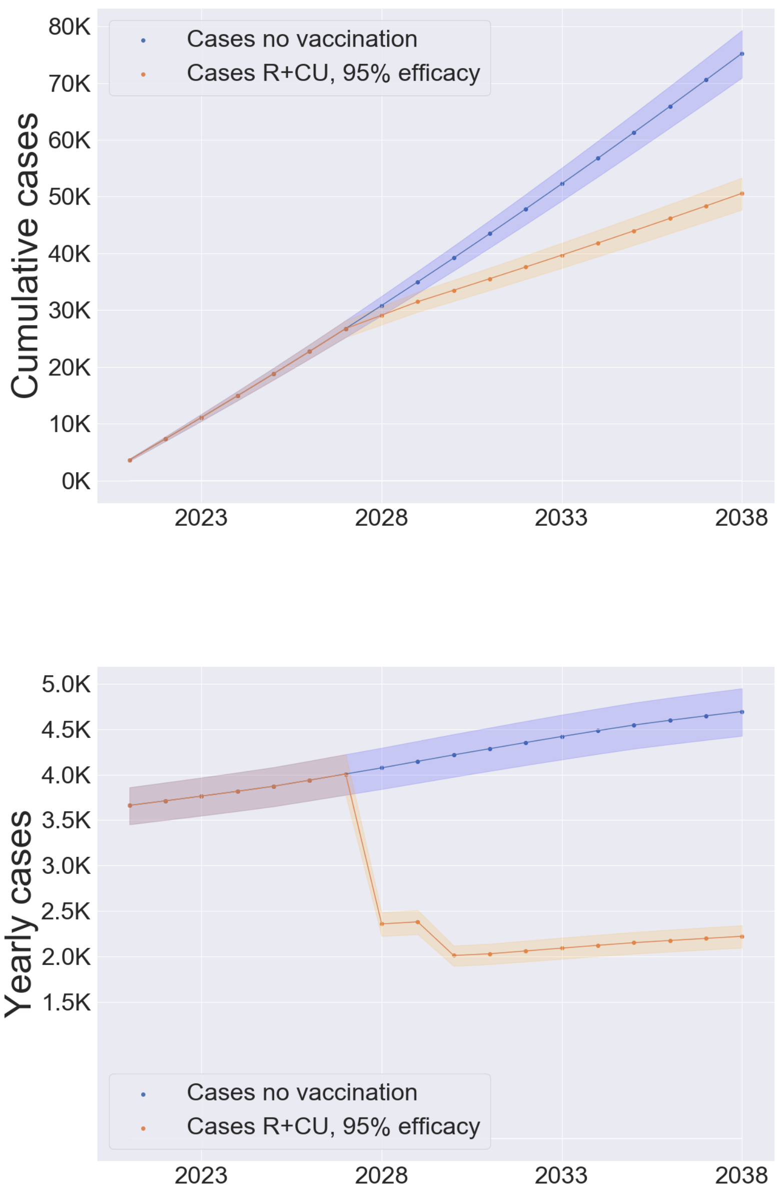

Supplementary Fig. 54 Togo cumulative and yearly iNTS cases: Togo cumulative (top) and yearly (bottom) iNTS cases under the status quo and routine + catch-up vaccination (95% efficacy) scenarios. Shaded areas show the 25th and 75th percentiles, line shows the median over 1000 experiments, samples drawn from uniform distributions over (0.00020,0.00024) for $\beta_{2,n}$ and (0.0080,0.0084) for $\beta_{4,n}$.

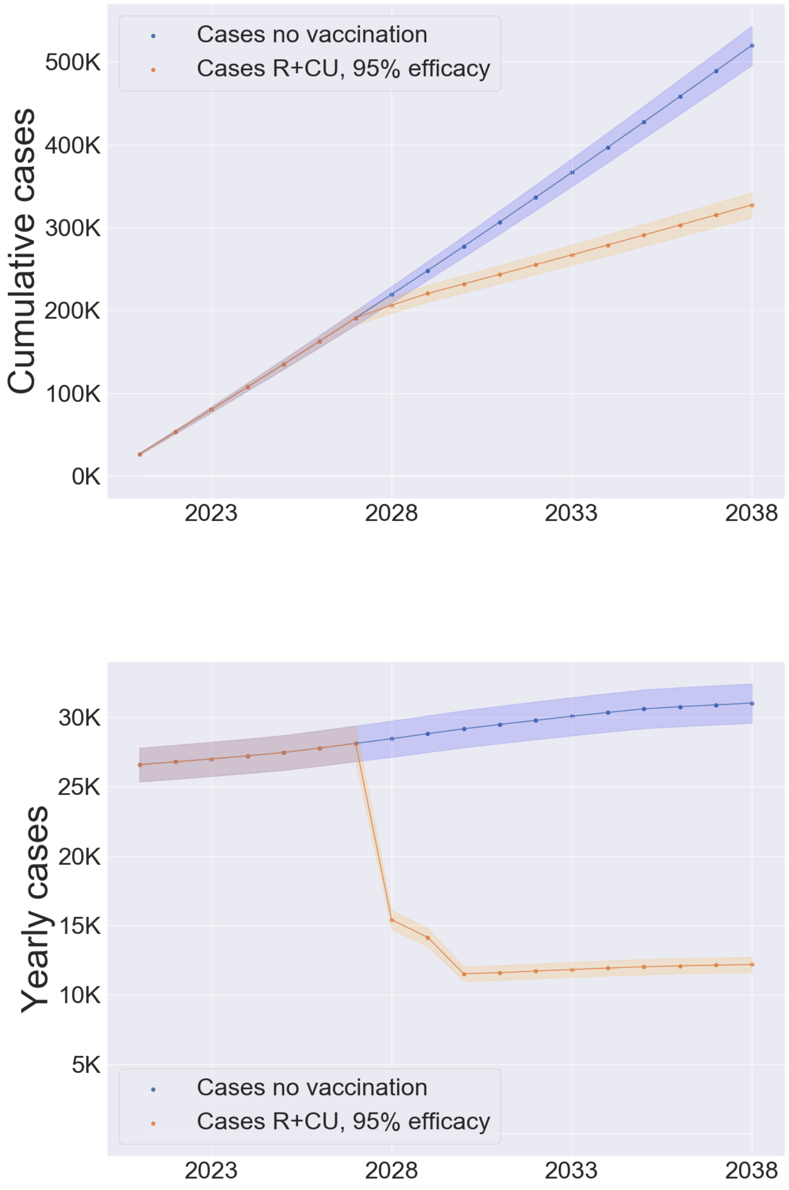

Supplementary Fig. 55 Uganda cumulative and yearly iNTS cases: Uganda cumulative (top) and yearly (bottom) iNTS cases under the status quo and routine + catch-up vaccination (95% efficacy) scenarios. Shaded areas show the 25th and 75th percentiles, line shows the median over 1000 experiments, samples drawn from uniform distributions over (0.00020,0.00024) for $\beta_{2,n}$ and (0.0080,0.0084) for $\beta_{4,n}$.

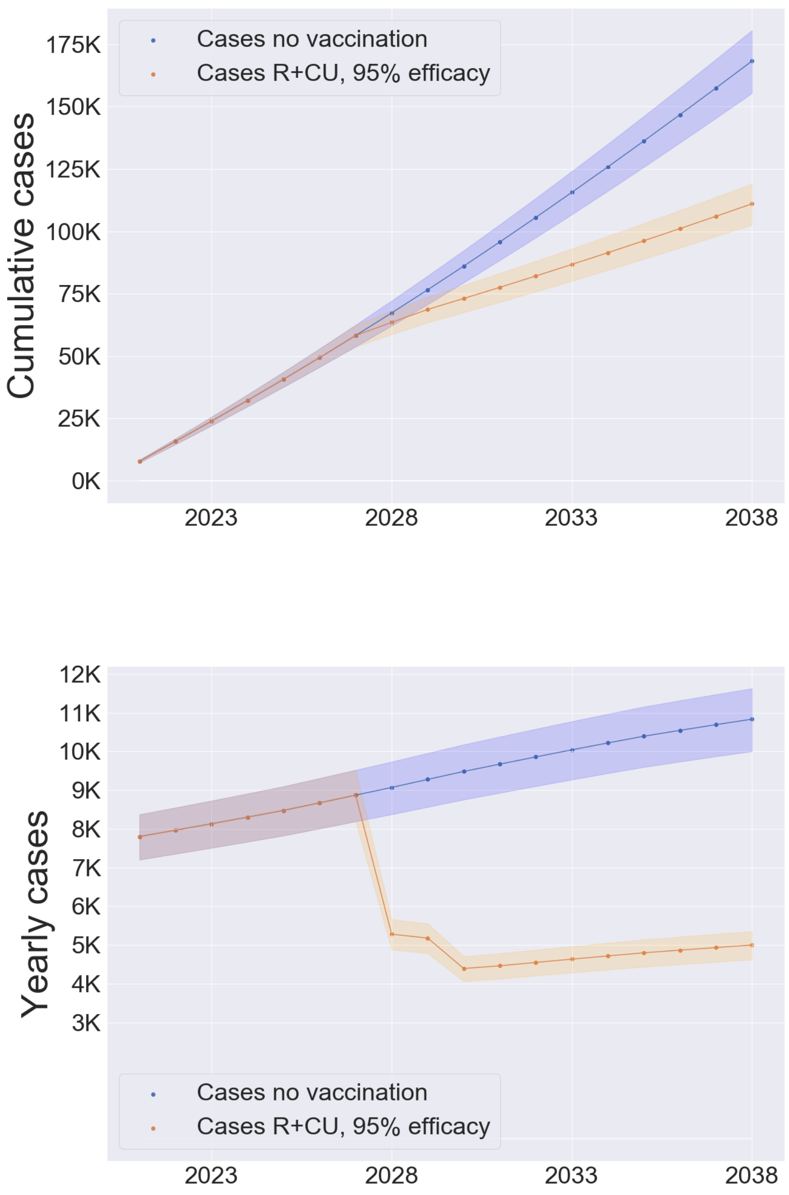

Supplementary Fig. 56 Zambia cumulative and yearly iNTS cases: Zambia cumulative (top) and yearly (bottom) iNTS cases under the status quo and routine + catch-up vaccination (95% efficacy) scenarios. Shaded areas show the 25th and 75th percentiles, line shows the median over 1000 experiments, samples drawn from uniform distributions over (0.00020,0.00024) for $\beta_{2,n}$ and (0.0080,0.0084) for $\beta_{4,n}$.

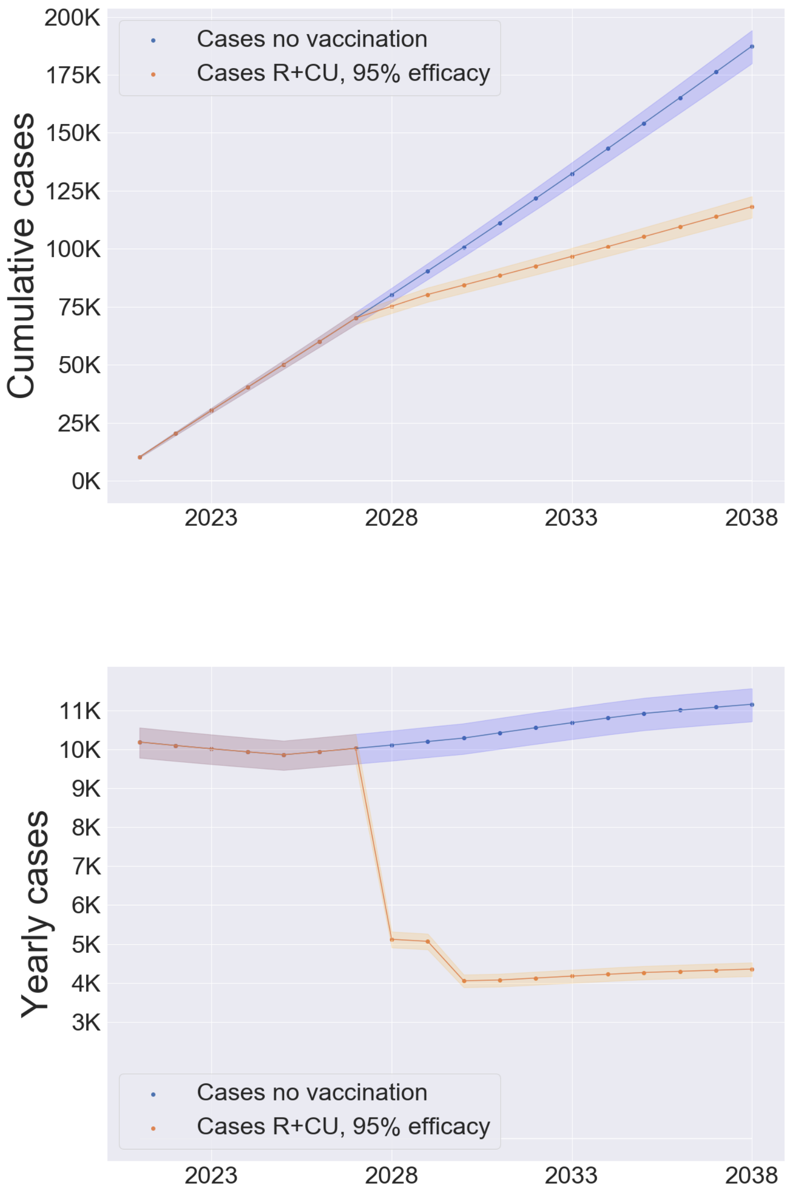

Supplementary Fig. 57 Zimbabwe cumulative and yearly iNTS cases: Zimbabwe cumulative (top) and yearly (bottom) iNTS cases under the status quo and routine + catch-up vaccination (95% efficacy) scenarios. Shaded areas show the 25th and 75th percentiles, line shows the median over 1000 experiments, samples drawn from uniform distributions over (0.00020,0.00024) for $\beta_{2,n}$ and (0.0080,0.0084) for $\beta_{4,n}$.

Table 5 Per-country cumulative DALYs averted (R and CU): All number are in thousands. The lower bound of cases prevented, deaths averted and DALYs averted correspond to 85% vaccine efficacy, the upper bound to 95% vaccine efficacy.

Country	Vaccination - Routine only			Vaccination - Catch-up only		
	Cases Prevented	Deaths averted	DALYs averted	Cases Prevented	Deaths averted	DALYs averted
Angola	(58.605, 65.718)	(8.791, 9.858)	(769, 862)	(7.936, 8.87)	(1.19, 1.331)	(104, 116)
Benin	(24.492, 27.867)	(3.674, 4.18)	(322, 366)	(2.704, 3.023)	(0.406, 0.453)	(36, 40)
Botswana	(8.143, 9.572)	(1.221, 1.436)	(107, 126)	(0.915, 1.023)	(0.137, 0.153)	(12, 14)
Burkina Faso	(52.546, 62.292)	(7.882, 9.344)	(690, 817)	(4.848, 5.419)	(0.727, 0.813)	(64, 71)
Burundi	(31.481, 37.781)	(4.722, 5.667)	(413, 496)	(2.879, 3.218)	(0.432, 0.483)	(38, 42)
Cabo Verde	(0.79, 0.915)	(0.119, 0.137)	(10, 12)	(0.098, 0.11)	(0.015, 0.017)	(1, 1)
Cameroon	(56.388, 63.42)	(8.458, 9.513)	(740, 832)	(7.288, 8.146)	(1.093, 1.222)	(96, 107)
CAR	(9.375, 10.528)	(1.406, 1.579)	(123, 138)	(1.316, 1.471)	(0.197, 0.221)	(17, 19)
Chad	(24.936, 27.922)	(3.74, 4.188)	(327, 367)	(3.663, 4.094)	(0.549, 0.614)	(48, 54)
Comoros	(2.899, 3.423)	(0.435, 0.513)	(38, 45)	(0.294, 0.328)	(0.044, 0.049)	(4, 4)
Congo	(10.998, 12.471)	(1.65, 1.871)	(144, 163)	(1.247, 1.394)	(0.187, 0.209)	(16, 18)
Côte d'Ivoire	(73.894, 87.759)	(11.084, 13.164)	(969, 1151)	(7.352, 8.217)	(1.103, 1.233)	(96, 108)
DRC	(130.873, 146.919)	(19.631, 22.038)	(1717, 1928)	(18.313, 20.469)	(2.747, 3.07)	(240, 268)
Djibouti	(1.433, 1.631)	(0.215, 0.245)	(19, 22)	(0.18, 0.202)	(0.027, 0.03)	(3, 3)
Equatorial Guinea	(2.974, 3.35)	(0.446, 0.502)	(39, 44)	(0.372, 0.416)	(0.056, 0.062)	(5, 5)
Eritrea	(7.235, 8.477)	(1.085, 1.272)	(95, 111)	(0.752, 0.84)	(0.113, 0.126)	(10, 11)
Eswatini	(3.638, 4.256)	(0.546, 0.638)	(47, 55)	(0.391, 0.438)	(0.059, 0.066)	(5, 5)
Ethiopia	(185.759, 209.435)	(27.864, 31.415)	(2438, 2749)	(23.752, 26.547)	(3.563, 3.982)	(312, 349)
Gabon	(3.87, 4.353)	(0.58, 0.653)	(51, 57)	(0.516, 0.577)	(0.077, 0.087)	(7, 8)
Gambia	(5.429, 6.298)	(0.814, 0.945)	(71, 83)	(0.56, 0.626)	(0.084, 0.094)	(7, 8)
Ghana	(71.496, 84.157)	(10.724, 12.624)	(938, 1105)	(7.534, 8.421)	(1.13, 1.263)	(99, 111)
Guinea	(19.365, 21.659)	(2.905, 3.249)	(255, 285)	(3.018, 3.373)	(0.453, 0.506)	(40, 45)
Guinea-Bissau	(3.182, 3.601)	(0.477, 0.54)	(41, 47)	(0.377, 0.421)	(0.057, 0.063)	(5, 5)
Kenya	(150.254, 175.593)	(22.538, 26.339)	(1972, 2304)	(16.388, 18.316)	(2.458, 2.747)	(215, 240)
Lesotho	(6.391, 7.389)	(0.959, 1.108)	(84, 97)	(0.718, 0.802)	(0.108, 0.12)	(9, 10)
Liberia	(11.786, 13.77)	(1.768, 2.065)	(155, 181)	(1.187, 1.326)	(0.178, 0.199)	(16, 18)
Madagascar	(51.345, 58.306)	(7.702, 8.746)	(674, 765)	(5.817, 6.501)	(0.873, 0.975)	(77, 85)
Malawi	(72.155, 85.493)	(10.823, 12.824)	(947, 1122)	(7.118, 7.955)	(1.068, 1.193)	(93, 104)
Mali	(39.083, 44.405)	(5.862, 6.661)	(513, 582)	(4.351, 4.864)	(0.653, 0.73)	(57, 64)
Mauritania	(12.729, 14.523)	(1.909, 2.178)	(167, 191)	(1.38, 1.543)	(0.207, 0.231)	(18, 20)
Mauritius	(1.267, 1.482)	(0.19, 0.222)	(16, 19)	(0.15, 0.167)	(0.022, 0.025)	(2, 2)
Mayotte	(0.833, 0.964)	(0.125, 0.145)	(11, 12)	(0.084, 0.093)	(0.013, 0.014)	(1, 1)
Mozambique	(120.222, 140.61)	(18.033, 21.092)	(1577, 1845)	(11.626, 12.994)	(1.744, 1.949)	(152, 170)
Namibia	(7.819, 8.983)	(1.173, 1.347)	(103, 118)	(0.886, 0.99)	(0.133, 0.149)	(12, 13)
Niger	(45.922, 53.166)	(6.888, 7.975)	(602, 697)	(4.334, 4.844)	(0.65, 0.727)	(57, 63)
Nigeria	(353.392, 395.272)	(53.009, 59.291)	(4638, 5187)	(52.273, 58.424)	(7.841, 8.764)	(686, 767)
Rwanda	(32.549, 39.161)	(4.882, 5.874)	(427, 513)	(3.264, 3.648)	(0.49, 0.547)	(42, 47)
Réunion	(1.134, 1.292)	(0.17, 0.194)	(14, 17)	(0.137, 0.153)	(0.021, 0.023)	(1, 2)
Sao Tome	(0.575, 0.671)	(0.086, 0.101)	(8, 9)	(0.061, 0.068)	(0.009, 0.01)	(1, 1)
Senegal	(35.87, 41.832)	(5.38, 6.275)	(471, 549)	(3.83, 4.28)	(0.574, 0.642)	(50, 56)
Seychelles	(0.133, 0.156)	(0.02, 0.023)	(2, 2)	(0.015, 0.017)	(0.002, 0.003)	(0, 0)
Sierra Leone	(16.957, 19.922)	(2.544, 2.988)	(222, 261)	(1.765, 1.972)	(0.265, 0.296)	(23, 26)
Somalia	(16.886, 18.829)	(2.533, 2.824)	(221, 247)	(3.031, 3.388)	(0.455, 0.508)	(39, 44)
South Africa	(134.766, 152.645)	(20.215, 22.897)	(1769, 2003)	(16.991, 18.99)	(2.549, 2.849)	(223, 249)
South Sudan	(14.599, 16.295)	(2.19, 2.444)	(192, 214)	(2.782, 3.11)	(0.417, 0.466)	(37, 41)
Togo	(19.762, 22.755)	(2.964, 3.413)	(260, 299)	(2.022, 2.26)	(0.303, 0.339)	(27, 30)
Uganda	(149.55, 178.868)	(22.432, 26.83)	(1963, 2347)	(15.278, 17.076)	(2.292, 2.561)	(201, 224)
Tanzania	(170.994, 196.187)	(25.649, 29.428)	(2244, 2574)	(17.539, 19.603)	(2.631, 2.94)	(230, 257)
Zambia	(45.444, 53.224)	(6.817, 7.984)	(596, 698)	(4.375, 4.89)	(0.656, 0.73[illegible])	(57, 64)
Zimbabwe	(55.314, 63.462)	(8.297, 9.519)	(726, 833)	(6.388, 7.14)	(0.958, 1.071)	(84, 94)

References

[1] Gilchrist, J. J., and MacLennan, C. A. Invasive nontyphoidal salmonella disease in africa. *EcoSal Plus 8*, 2 (2019).

[2] Hansen-Wester, I., Stecher, B., and Hensel, M. Type iii secretion of salmonella entericaserovar typhimurium translocated effectors and ssefg. *Infection and Immunity 70*, 3 (2002), 1403–1409.

[3] Herman, J., and Usher, W. SALib: An open-source python library for sensitivity analysis. *The Journal of Open Source Software 2*, 9 (jan 2017).

[4] Karachaliou, A., Conlan, A. J. K., Preziosi, M.-P., and Trotter, C. L. Modeling long-term vaccination strategies with menafrivac in the african meningitis belt. *Clinical Infectious Diseases 61*, suppl5 (11 2015), S594–S600.

[5] Marino, S., Hogue, I. B., Ray, C. J., and Kirschner, D. E. A methodology for performing global uncertainty and sensitivity analysis in systems biology. *Journal of Theoretical Biology 254*, 1 (2008), 178–196.

[6] Moore, B. C. e. a. Survival of salmonella enterica in freshwater and sediments and transmission by the aquatic midge chironomus tentans (chironomidae: Diptera). *Applied and environmental microbiology 69*, 8 (2003).

[7] Okoro, C. K., et al. Intracontinental spread of human invasive salmonella typhimurium pathovariants in sub-saharan africa. *Nature Genetics 44*, 11 (Nov 2012), 1215–1221.

[8] Saltelli, A., et al. *Global Sensitivity Analysis. The Primer.* John Wiley & Sons, Ltd, 2008.

[9] United Nations. World population prospects 2019, 2020. https://population.un.org/wpp/Download/Standard/Population/, Accessed October 2021.
